# Cancer burden and risk in the Chinese population aged 55 years and above: A systematic analysis and comparison with the USA and Western Europe

**DOI:** 10.7189/jogh.14.04014

**Published:** 2024-01-26

**Authors:** Teng-Yu Gao, Yu-Ting Tao, Hao-Yang Li, Xin Liu, Yu-Tong Ma, Hui-Jun Li, Chen-Yang Xian-Yu, Nian-Jia Deng, Wei-Dong Leng, Jie Luo, Chao Zhang

**Affiliations:** 1 Center for Evidence-Based Medicine and Clinical Research, Taihe Hospital, Hubei University of Medicine, Shiyan, China; 2 Department of Stomatology, Taihe Hospital, Hubei University of Medicine, Shiyan, China; 3 Department of Neurosurgery, Taihe Hospital, Hubei University of Medicine, Shiyan, China

## Abstract

**Background:**

We analysed the cancer burden among elderly Chinese people over the age of 55 years and compared them to USA and Western Europe to explore the cancer model in China.

**Methods:**

We retrieved data on 29 cancers with 34 risk factors from the 2019 Global Burden of Disease database to evaluate the cancer burden in Chinese elderly individuals aged 55 years and older. We then used the age-standardised incidence rate (ASIR), age-standardised death rate (ASDR), age-standardised disability-adjusted life year (DALY) rate, and average annual percentage change (AAPC) to compare the characteristics and change trend of cancers among China, USA, and Western Europe.

**Results:**

In 2019, the number of incident cases of 29 cancers among people aged 55 years and above in China increased more than 3-fold compared to 1990, while the number of deaths and DALYs approximately doubled. We also found that the cancer population in China was ageing; meanwhile, the cancer burden became significantly higher for men than for women, and the gap between men and women had widened. Cancers with the highest cancer DALYs were lung cancer (13 444 500; 95% uncertainty interval (UI) = 11 307 100, 15 853 700), stomach cancer (7 303 900; 95% UI = 6 094 600, 8 586 500), oesophageal cancer (4 633 500; 95% UI = 3 642 500, 5 601 200), colon and rectum cancer (4 386 500; 95% UI = 3 769 500, 5 067 200), liver cancer (2 915 100, 95% UI = 2 456 300, 3 463 900), and pancreatic cancer (2 028 400; 95% UI = 1 725 000, 2 354 900). Compared with 1990, the DALY rate and incidence rate of stomach cancer, oesophageal cancer, and liver cancer had markedly decreased. The DALY rate and incidence rate of lung, colon, rectum, and pancreatic cancer had increased significantly, as did the incidence rate of breast cancer in women. Smoking and diet were the top two cancer risk factors, and the impact of ambient particulate matter pollution on cancer increased each year. The overall 29 cancers age-standardised DALY rate and ASDR in China, USA, and Western Europe were similar, and all showed downward trend in the past 30 years. Compared with the USA and Western Europe, the age-standardised DALY rate of liver, nasopharyngeal, oesophageal, stomach, and cervical cancers in China was more prominent. The age-standardised DALY rate of lung cancer and colon and rectum cancer decreased annually in Western Europe and the USA, but increased in China.

**Conclusions:**

Over the past 30 years, China had made progress in controlling stomach, oesophageal, and liver cancer. However, lung, colon, rectum, pancreatic, and breast cancers had become more prevalent, having risen alongside economic development. The risks of smoking and dietary were major issues that need to be addressed urgently. The cancer situation in China remains serious; future cancer prevention efforts need to balance economic development with people's physical health, identify key groups, improve the health environment of residents and guide them to live a healthy life, and expand the scope of cancer screening.

Cancer was one of the leading causes of death in China in 2020, with approximately 3 million cancer deaths and 4.57 million new cases [1]. Projections suggested that the number of cancer deaths will continue to increase in the coming decades due to population growth and ageing; unhealthy lifestyles such as insufficient physical exercise and excessive intake of high-calorie foods; and the impact of rapid economic growth on the environment [2,3]. Research has shown that the cancer spectrum in China has changed significantly because of urbanisation and socioeconomic development, but also by the transition from a developing to a developed country [4]. Although the high incidence and mortality of oesophageal, stomach, and liver cancer caused significant concern in past decades, the rapid rise in the incidence and mortality of lung, colorectal, and breast cancer had also attracted attention [5]. China’s population was likewise ageing. The proportion of Chinese population aged 60 years and above rose from 13.3% in 2010 to 18.7% in 2020 and was expected to reach 35% by 2050 [6]. This also meant a possible increase in the incidence of cancer [6], as it is known to increase with age, and as the elderly are at a high risk of cancer [7]. Thus, there is growing concern that the cancer burden in China will continue to rise as the elderly population grows.

We therefore retrieved cancer data in Chinese population aged 55 years and above from Global Burden of Disease (GBD) to explore the cancer spectrum transformation between 1990 and 2019 in the older Chinese population in terms of age, sex, and risk factors. We also sought to compare the changes and characteristics of cancer in China with those in the USA and Western Europe, in order to find the differences in the characteristics of cancer prevalence or the deficiencies in cancer control between China and high-income countries. These findings could advance our understanding of the development of cancer in the past and could be used to support measures that are more in line with the current situation in the country, thus helping achieve public health goals such as the Healthy China 2030 Plan [8,9].

## METHODS

### Overview

The GBD study provides comprehensive and comparable disease burden estimates, including incident cases, deaths, disability-adjusted life years (DALYs), and prevalence, for 369 causes and 87 risk factors for 204 countries and territories, categorised by age, sex, and year. We used data from the latest GBD 2019 study [10] to assess the cancer burden in the Chinese population aged 55 years and above and to compare the cancer burden differences between China, USA, and Western Europe.

### Data sources

The neoplasm list in the GBD 2019 comprised 30 cancers, which included all codes defining neoplasms in the International Classification of Diseases 9th (140–239) and 10th edition (C00–D49) [11,12]. We excluded ‘Other neoplasms’ because the burden of this type of cancer was too small compared to others. Cancer categories and corresponding ICD codes can be found in Table S1 in the **Online Supplementary Document**. We retrieved the incidence rates, death rates, DALY rates, incident cases, deaths, and DALYs of 29 types of cancer in China, USA, and Western Europe for population aged 55 years and above between 1990 and 2019 using the Global Health Data Exchange query tool [13]. To better understand the cancer burden, we looked at gender and age differences in cancer, dividing the age groups 55–64, 65–74, 75–84, and >85 years.

There were 69 specific risk factors for GBD 2019, 34 of which contributed to the burden of 23 cancers [14]. Based on these risk factors, we compared the burden difference between men and women. The risk factors of diets high in processed meat, high in red meat, high in sodium, low in calcium, low in fibre, low in fruits, low in milk, low in vegetables, and low in whole grains were summed up as dietary risk. Occupational exposure to arsenic, asbestos, benzene, beryllium, cadmium, chromium, diesel engine exhaust, formaldehyde, nickel, polycyclic aromatic hydrocarbons, silica, sulfuric acid, and trichloroethylene were considered occupational carcinogens.

In the GBD 2019 study, Western Europe comprised Switzerland, Finland, San Marino, UK, Germany, France, Ireland, Greece, Luxembourg, Iceland, Monaco, Andorra, Belgium, Israel, Austria, Italy, Denmark, Malta, Cyprus, Netherlands, Spain, Portugal, Norway, and Sweden.

#### Statistical analyses

DALYs are the sum of years of life lost (YLLs) and years lived with disability (YLDs), which incorporate both fatal and nonfatal burdens [15]. Aside from the DALYs, we also calculated the age-standardised rates (ASRs) and their average annual percentage change (AAPC) in China, USA, and Western Europe to compare the different characteristics of cancer changes over the past 30 years. When comparing the cancer control circumstances in different regions, the differences in susceptibility of their populations to the disease is biased due to the differences in their age structures. Therefore, the direct comparison of incidence rate and death rate between different regions cannot accurately reflect which one has better medical and health conditions.

Age-standardised rates are commonly used to eliminate the effects of different age structures in different regions on incidence rate and death rate, among others [16]. We calculated the ASRs of incidence, death, and DALY based on GBD 2019 standard populations as follows:



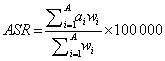


where a_i_ is the rate in the i_th_ age group and w_i_ is the GBD standard population number of the i_th_ age group [12,17]. GBD standard populations were previously described in other study [18].

Based on the ASRs, we calculated AAPCs using the Joinpoint Regression Programme, version 4.9.1.0. (National Cancer Institute, National Institute of Health, Maryland, USA) In the past, annual percentage change (APC) was often used to estimate the change of ASR over time; however, it requires this change to be linear, making its research scope limited. Obtaining AAPCs by a weighted average of APCs over different time intervals, weighted according to their time span, allows for an estimation of the change in ASR over the relevant time interval, even though ASR experiences distinct upward and downward trends over that period [19,20]. The obtained AAPC and its CI can represent the overall change trend of ASR in the selected time interval. The ASR is considered to have an increasing trend when the 95% CI of AAPC is >0; a decreasing trend when the 95% CI is <0; and a stable trend when the 95% CI contains 0 [21]. Here we reported both crude rates and ASRs per 100 000. If not specifically mentioned, the population described in the results is the Chinese population aged 55 years and above. We prepared all calculations and graphics using R, version 4.3.1 (R Core Team, Vienna, Austria).

#### Uncertainty analysis

The data derived from GBD is presented with 95% uncertainty intervals (UIs), quantified by using the 2.5th and 97.5th percentiles of the 1000 ordered values in 1000 draws from posterior distribution of each stage in the GBD estimation process.

## RESULTS

### Trends of 29 types of cancer in Chinese population aged 55 years and above

We obtained data on 29 recorded cancers from the 2019 GBD database. In China, the number of overall incident cases (3 458 900; 95% UI = 2 840 800, 4 127 200) among people aged 55 years and above in 2019 was three times that of 1990, while deaths (2 243 510; 95% UI = 1 849 780, 2 649 880) and DALYs (46 082 900; 95% UI = 37 934 300, 54 763 000) were nearly 2-fold higher (**Table 1**; Table S2–3 in the **Online Supplementary Document**).

**Table 1:** The DALYs and age-standardised DALY rate of 29 cancers among China, USA and Western Europe in 1990 and 2019, and the AAPC of age-standardised DALY rate from 1990 to 2019*

	DALYs ×1000 in 1990 (95% UI)			DALYs ×1000 in 2019 (95% UI)			Age-standardised DALY rate in 1990 (95% UI)			Age-standardised DALY rate in 2019 (95% UI)			AAPC (95% CI)		
**Cause**	**China**	**USA**	**Western Europe**	**China**	**USA**	**Western Europe**	**China**	**USA**	**Western Europe**	**China**	**USA**	**Western Europe**	**China**	**USA**	**Western Europe**
All malignant neoplasms	23 319.6 (19 210.4, 27 461.5)	9338.2 (8695.6, 9753.3)	16 478.1 (15 493.1, 17 248)	46 082.9 (37 934.3, 54 763)	13 396.7 (12 390.9, 14 571.3)	20 345.3 (18 460.7, 22 069.8)	16 189.7 (13 203.7, 19 220.6)	17 234.6 (15 906.6, 18 149.5)	16 541.3 (15 377, 17 531.5)	13 273.4 (10 812.8, 15 838.3)	13 696.2 (12 459.4, 15 147.8)	13 304.3 (11 868.2, 14 764.1)	−0.72 (–0.88, –0.56)	−0.80 (−0.92, –0.67)	–0.78 (–0.87, –0.69)
Bladder cancer	289.1 (252.7, 326.1)	200.8 (191.9, 207.7)	631 (608.6, 651.1)	671 (569.4, 792.8)	353.7 (329.3, 375.6)	752.7 (686.5, 813.4)	229.1 (196.7, 262.3)	356.9 (336.7, 373.5)	616.6 (584.7, 645)	204.3 (171.3, 241.5)	354.8 (324.3, 382.9)	457.3 (408, 504.9)	–0.42 (–0.63, –0.22)	–0.02 (–0.1, 0.06)	–1.08 (–1.15, –1)
Brain and central nervous system cancer	380.3 (306.7, 506.1)	185.4 (159, 221.8)	323.6 (284, 407.5)	909.3 (675.6, 1110.6)	337.2 (254.4, 362.8)	493.3 (306.1, 555.5)	255 (199.7, 344.3)	353.7 (297.1, 423.3)	337.5 (289.1, 424.9)	256.9 (187.2, 318.6)	350.2 (262.6, 392.5)	349.6 (218.5, 403)	0.02 (–0.1, 0.14)	–0.05 (–0.17, 0.08)	0.12 (0.05, 0.19)
Breast cancer	592.9 (501.1, 695.8)	831.2 (790.3, 873.6)	1466 (1398.5, 1520.8)	1629.3 (1343.7, 1959)	1006.5 (933.8, 1078.6)	1581.5 (1451.2, 1696.2)	400.7 (335.4, 474.2)	1566.1 (1474.3, 1651)	1497.6 (1415.2, 1569.2)	461.3 (377.7, 556.1)	1034.1 (940, 1123.8)	1048.1 (944.9, 1147.2)	0.45 (0.02, 0.88)	–1.45 (–1.66, –1.24)	–1.23 (–1.3, –1.16)
Cervical cancer	384.6 (303.3, 616.5)	83.1 (75, 87)	195.5 (179.7, 204.2)	804.4 (461.3, 1026.7)	122.8 (101.6, 131.1)	169.7 (149.8, 184.8)	261.6 (205.5, 418.2)	159.4 (139.3, 169.7)	199.6 (179.2, 215.2)	225.7 (130.6, 289.4)	128.2 (105.5, 140)	115.1 (100.7, 129.3)	–0.54 (–0.95, –0.13)	–0.74 (–0.9, –0.58)	–1.86 (–2.01, –1.72)
Colon and rectum cancer	1271.8 (1113.1, 1426)	1119.3 (1073.5, 1153.7)	2172 (2086.8, 2225)	4386.5 (3769.5, 5067.2)	1408.4 (1333.9, 1466.7)	2621.8 (2434.3, 2754.6)	923.1 (804.6, 1048.5)	2031.6 (1934.7, 2106.1)	2148.1 (2050.1, 2224.7)	1284.6 (1095.7, 1487.2)	1435.3 (1340.9, 1514.7)	1653.7 (1515.6, 1765.7)	1.12 (0.89, 1.35)	–1.19 (–1.37, –1)	–0.92 (–1.01, –0.83)
Esophageal cancer	3353.9 (2118.3, 3922.9)	208.4 (202.3, 213.8)	454.3 (443, 462.9)	4633.5 (3642.5, 5601.2)	395.5 (378.1, 411.3)	591.1 (556.5, 621)	2330.7 (1471.3, 2755.6)	393.8 (378.1, 408.6)	465.5 (447.1, 482.2)	1333.5 (1009.8, 1611.7)	408.1 (382.3, 432.5)	404.1 (370.1, 439)	–1.98 (–2.1, –1.85)	0.11 (0.01, 0.2)	–0.49 (–0.54, –0.45)
Gallbladder and biliary tract cancer	207 (169.4, 349.6)	73.4 (62.1, 78.1)	292.5 (229.1, 306.5)	586.9 (432.9, 703.3)	88.8 (81.6, 106.2)	254.3 (213.9, 290.2)	152 (123.4, 251.9)	132.3 (110.1, 144.9)	288.2 (223.2, 309.7)	171.9 (124.4, 206.7)	90.3 (81.2, 109.8)	160.6 (135.6, 187.9)	0.42 (0.27, 0.57)	–1.32 (–1.38, –1.25)	–2.01 (–2.11, –1.91)
Hodgkin lymphoma	57.8 (30.4, 75)	19.8 (16.3, 21.8)	42.9 (35.1, 47)	42.2 (30.7, 51.1)	23.5 (20.8, 29.5)	36 (31.1, 43.4)	39.4 (20.4, 52.1)	37.1 (29.5, 41.7)	43.5 (34.9, 48.6)	12 (8.5,14.9)	24.2 (20.7, 31.1)	23.9 (20.3, 29.6)	–4.08 (–4.38, –3.78)	–1.46 (–1.59, –1.33)	–2.05 (–2.19, –1.91)
Kidney cancer	83.8 (72.1, 96.6)	204 (196.6, 211.2)	355.4 (342.8, 365.7)	386.6 (320.6, 457.7)	355.5 (334.2, 374.9)	549.2 (512.5, 578.7)	59.1 (50.2, 68.8)	381 (362.1, 398.3)	359.5 (338.1, 381.1)	111.8 (92.4, 133.6)	364.9 (333, 394.7)	359.4 (323, 396)	2.20 (1.98, 2.41)	–0.16 (–0.3, –0.03)	–0.02 (–0.1, 0.06)
Larynx cancer	209.4 (178.2, 241.5)	82.9 (80.1, 85.3)	249 (242.4, 256)	380.6 (315.6, 454.4)	99.8 (94.9, 104.7)	180.2 (169.3, 190.2)	144.6 (122, 168.1)	157.7 (150.3, 164.4)	258.6 (246.6, 270.9)	108.4 (89, 130.1)	103.2 (96.4, 110.2)	125.9 (114.4, 137.7)	–1.00 (–1.15, –0.84)	–1.46 (–1.6, –1.32)	–2.51 (–2.56, –2.46)
Leukemia	405.9 (345.2, 481.7)	316.9 (303.3, 326.2)	465.1 (446.7, 477.6)	759.4 (630.1, 897)	483.5 (452.8, 506.7)	665.8 (602.2, 704.5)	276.6 (228.7, 338.5)	575.6 (545.4, 599)	462.2 (439, 481.3)	215.6 (176.2, 258.3)	489.1 (450.3, 523.1)	416.7 (373.1, 453.3)	–0.89 (–1.02, –0.76)	–0.55 (–0.66, –0.45)	–0.36 (–0.47, –0.24)
Lip and oral cavity cancer	115.8 (101, 130.9)	109.3 (105.5, 112.7)	215.2 (208.4, 221.3)	387.2 (323.7, 464.9)	144.7 (137.4, 151.1)	247 (232, 259.6)	82.6 (71.1, 94.2)	206.8 (197, 215.6)	224.4 (213.3, 235.1)	111.3 (91.8, 133.6)	149 (138.3, 158.8)	172.1 (157.8, 186.2)	0.96 (0.71, 1.21)	–1.1 (–1.27, –0.93)	–0.92 (–1.06, –0.78)
Liver cancer	3396.3 (2884.8, 4096.2)	109.3 (105.8, 112)	360.1 (348.8, 369.7)	2915.1 (2456.3, 3463.9)	450.7 (401.2, 494.5)	668.3 (622.7, 710.1)	2274.9 (1903.1, 2755.2)	202.8 (193.6, 210.8)	363.1 (345.4, 380.5)	821.9 (687.2, 975.8)	468.7 (408.4, 522.1)	447.2 (405.5, 488.3)	–3.43 (–3.79, –3.07)	2.93 (2.76, 3.1)	0.71 (0.63, 0.8)
Malignant skin melanoma	34.6 (24.4, 52.6)	106.4 (78.3, 137.6)	136 (110.4, 191)	75.4 (48.4, 94.2)	212 (148, 265.4)	257.2 (151.2, 291.7)	24.8 (17.5, 37.1)	201.6 (144.7, 257.4)	138.9 (110.2, 194.5)	22.1 (13.9, 28)	218.1 (152.9, 282.1)	172.5 (102.7, 201.8)	–0.38 (–0.49, –0.26)	0.29 (0.1, 0.48)	0.75 (0.63, 0.87)
Mesothelioma	16.2 (12.5, 22.7)	39.6 (37.2, 42.3)	118 (108.1, 129.3)	41.2 (33.9, 49.3)	53.4 (49.7, 57.3)	174.6 (161.9, 184.1)	11.3 (8.5, 16.3)	71.6 (65.2, 78.4)	118.9 (106.1, 133.3)	11.8 (9.4, 14.4)	54.2 (49.5, 59.2)	114.8 (104.5, 124.7)	0.05 (–0.18,0.29)	–0.93 (–1.07, –0.8)	–0.12 (–0.22, –0.02)
Multiple myeloma	97.9 (79, 136.8)	182.1 (150.7, 189.8)	240 (220.5, 273.1)	242.5 (183.4, 298.5)	311.6 (287.3, 376.4)	416.8 (356.1, 445.8)	67 (53.4, 94)	331.5 (268.7, 349.7)	237.9 (211.9, 269.3)	68.5 (50.8, 85.3)	317.2 (285.2, 389.5)	265.2 (221.9, 298.9)	0.04 (–0.11, 0.19)	–0.15 (–0.21, –0.09)	0.38 (0.29, 0.46)
Nasopharynx cancer	360.2 (309.8, 412.9)	16.7 (16.1, 17.2)	40.7 (39.1, 42.4)	456 (380.4, 538.2)	20.7 (19.6, 21.9)	35.7 (33, 38.4)	240.6 (201.7, 284.5)	32.8 (30.9, 34.6)	43.1 (40, 46.5)	127.6 (105.3, 152.4)	21.8 (20.1, 23.7)	25.8 (22.7, 29.3)	–2.27 (–2.52, –2.03)	–1.41 (–1.51, –1.3)	–1.76 (–1.87, –1.66)
Non-Hodgkin lymphoma	202.8 (175.8, 230.7)	353.1 (338.2, 363.9)	423.2 (408.1, 435.7)	728.4 (616.1, 852.3)	495.8 (466.6, 520.5)	611.2 (559.8, 653.7)	140.9 (121.6, 161.8)	645.1 (615.2, 669.6)	423.4 (403.7, 440.7)	206.7 (173.4, 243.3)	502.8 (466.4, 534.9)	386.9 (350.7, 419.7)	1.30 (1.13, 1.46)	–0.86 (–1.03, –0.68)	–0.35 (–0.44, –0.26)
Non-melanoman skin cancer	79.5 (69.2, 89.5)	69.2 (56.6, 86.4)	50.4 (47.1, 52)	244.7 (206, 282.9)	132.3 (108.8, 161.5)	79 (69.7, 83.9)	60.5 (52.1, 69.5)	125.4 (99.6, 162.4)	50.3 (46.2, 53.1)	74.2 (61.5, 86.6)	132.8 (107.2, 166.2)	46.1 (40.8, 50)	0.68 (0.24, 1.13)	0.18 (0.02, 0.35)	–0.28 (–0.39, –0.18)
Other malignant neoplasms	527.3 (457.3, 610.2)	190.7 (174.1, 207)	490.5 (421.3, 513.6)	1140.7 (914.5, 1348.7)	331.9 (296.8, 354.8)	592.1 (511, 640.5)	371.7 (317, 436.6)	352.1 (313.5, 387.7)	495.3 (413.8, 526.5)	330 (262.5, 390.2)	339.9 (296.6, 376)	383.8 (324.9, 424.1)	–0.44 (–0.59, –0.29)	–0.12 (–0.18, –0.07)	–0.87 (–0.99, –0.75)
Other pharynx cancer	46.9 (40.4, 53.6)	38.7 (37.3, 40.2)	116.8 (113, 121)	96.8 (78, 117.1)	63.7 (60.3, 67)	180 (166.9, 194.4)	32.5 (27.6, 37.9)	74.9 (71, 79.1)	124.6 (116.8, 132.9)	27.4 (22, 33.4)	66.6 (61.4, 72.1)	133.4 (116.1, 153.3)	–0.59 (–0.72, –0.46)	–0.39 (–0.61, –0.17)	0.24 (0.1, 0.37)
Ovarian cancer	113 (85.9, 176.7)	247.5 (223.9, 257.4)	454.5 (411.1, 470.2)	490.1 (338.3, 620)	337.6 (312.1, 364.1)	495.6 (447.7, 536.4)	76.7 (58, 121.7)	461.7 (415.4, 485.9)	462.2 (413.2, 487.4)	136.9 (93.8, 175)	346.9 (313.3, 386.5)	332.8 (293.7, 373.6)	1.99 (1.81, 2.17)	–0.97 (–1.13, –0.8)	–1.13 (–1.29, –0.97)
Pancreatic cancer	474.5 (413.4, 541.5)	490.4 (471.8, 501.9)	839.7 (810.6, 856.5)	2028.4 (1725, 2354.9)	991.2 (939.2, 1032.7)	1456.2 (1355.3, 1538.3)	330 (283.6, 378.9)	897.7 (856.9, 928.1)	837.9 (801.1, 866.7)	581.5 (490.6, 679.5)	1014.4 (945.7, 1077)	955.4 (869.9, 1036.2)	1.95 (1.71, 2.18)	0.47 (0.25, 0.69)	0.44 (0.31, 0.56)
Prostate cancer	369.4 (282.8, 445.2)	689.1 (475, 767.1)	1038.5 (785.1, 1248.9)	948.9 (753.9, 1248)	894.8 (772.2, 1316.6)	1467.2 (1234.7, 2062.3)	297.6 (220.9, 375.4)	1199.2 (807.3, 1362)	987.3 (734.4, 1207)	292.1 (227.3, 388.3)	896.5 (749.9, 1335.6)	867.9 (719.8, 1259.2)	–0.10 (–0.27, 0.08)	–1.00 (–1.09, –0.92)	–0.45 (–0.53, –0.37)
Stomach cancer	5404.4 (4717.8, 6131.4)	304.4 (292.7, 311.8)	1306.1 (1258.9, 1334)	7303.9 (6094.6, 8586.5)	303.2 (288, 315.4)	921 (851.5, 968.9)	3768.6 (3261.2, 4295.2)	551.3 (524.8, 570.7)	1291.7 (1234.7, 1333.9)	2107.9 (1752.4, 2490.9)	309.2 (287.5, 328)	585.6 (535.4, 628.5)	–2.02 (–2.27, –1.77)	–1.98 (–2.15, –1.81)	–2.70 (–2.84, –2.57)
Testicular cancer	4.7 (3.9, 5.7)	2.5 (2.3, 2.7)	7.1 (6.7, 7.5)	14.2 (11.4, 17.2)	4.6 (4.2, 5.1)	8 (7.2, 8.9)	3.4 (2.7, 4.2)	4.8 (4.3, 5.4)	7.3 (6.5, 8.1)	4.2 (3.3, 5.1)	4.9 (4.2, 5.6)	5.5 (4.5, 6.6)	0.74 (0.47, 1.01)	0.03 (–0.04, 0.1)	–0.95 (–1.07, –0.83)
Thyroid cancer	50 (42.8, 64.9)	23.3 (21.7, 24.7)	67 (62.6, 69.7)	117.9 (99, 137.4)	48.3 (43.9, 52)	71 (63.4, 76.5)	36.5 (30.9, 48.1)	42.9 (39, 46.4)	66.7 (60.7, 71)	34.9 (28.6, 41.5)	49.5 (43.7, 54.6)	46.4 (40.5, 51.7)	–0.19 (–0.42, 0.05)	0.51 (0.45, 0.56)	–1.26 (–1.43, –1.08)
Tracheal, bronchus, and lung cancer	4619.8 (3987.2, 5313.1)	2931 (2854.5, 2983.6)	3757.9 (3676.2, 3811.3)	13 444.5 (11 307.1, 15 853.7)	3709.5 (3539.7, 3836.6)	4520.8 (4298.6, 4677)	3182.7 (2727.4, 3681.4)	5487.8 (5317.7, 5612.3)	3822.5 (3715.9, 3911.5)	3867.3 (3228.7, 4577.8)	3800.8 (3593.5, 3977.9)	3086.1 (2889.6, 3256.8)	0.63 (0.4, 0.85)	–1.25 (–1.39, –1.12)	–0.74 (–0.82, –0.67)
Uterine cancer	169.8 (132, 209.5)	109.9 (103.8, 115.1)	169.1 (160.7, 176.6)	217.2 (172.2, 314)	215.3 (200.5, 230.4)	247.9 (224.5, 266.8)	115.5 (87.8, 145.9)	199.4 (184.2, 212.9)	168.6 (156.1, 180.6)	61.2 (47.5, 89.1)	220.7 (198.2, 242.8)	162.5 (142.8, 181.6)	–2.21 (–2.63, –1.79)	0.33 (0.19, 0.46)	–0.12 (–0.22, –0.01)

AAPC – average annual percentage change, CI – confidence interval, DALY – disability-adjusted life-year, UI – uncertainty interval

*Estimates are for populations aged 55 y and above, both sexes combined. All malignant cancers are the sum of the following 29 cancers. Rates are reported per 100 000 person-years. Cancer types are listed alphabetically. Other malignant neoplasms are cancers without a detailed GBD cause separately listed.

**Figure 1:**
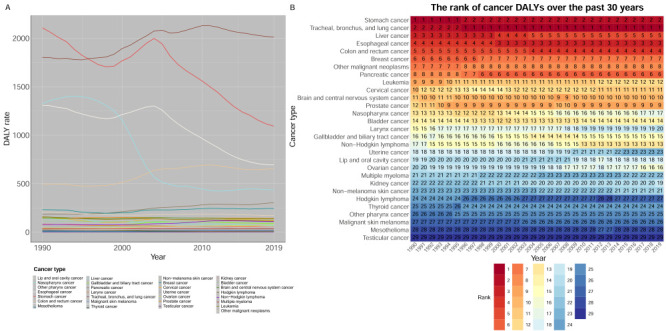
The changing trend of DALY rate and the rank of DALYs for 29 cancers in China from 1990 to 2019. **Panel A.** DALY rate. **Panel B.** The rank of DALYs. Estimates are for populations aged 55 years and above, both sexes in China. Rates are reported per 100 000 person-years. DALY – disability-adjusted life-year.

In 2019, the top six cancers with the highest DALYs were lung cancer (comprising tracheal, bronchus, and lung cancer) (13 444 500; 95% UI = 11 307 100, 15 853 700), stomach cancer (7 303 900; 95% UI = 6 094 600, 8 586 500), oesophageal cancer (4 633 500; 95% UI = 3 642 500, 5 601 200), colon, and rectum cancer (4 386 500, 95% UI = 3 769 500, 5 067 200), liver cancer (2 915 100; 95% UI = 2 456 300, 3 463 900), and pancreatic cancer (2 028 400; 95% UI = 1 725 000, 2 354 900). The DALYs of the remaining 23 cancers all had <2 000 000 DALYs. The DALY rate of lung cancer increased over 30 years, and its DALYs far exceeded that of stomach cancer, making it the most burdensome cancer in 2019. The DALY rate of stomach cancer and oesophageal cancer decreased, but DALYs remained high. Both the DALYs and DALY rate of liver cancer decreased significantly, while the DALYs and DALY rate of colon and rectum cancer and pancreatic cancer rapidly increased (**Figure 1**, **Table 1**).

In 2019, we observed the highest number of cases for lung cancer (721 900; 95% UI = 608 600, 847 300), stomach cancer (496 400; 95% UI = 418 000, 586 000), colon and rectum cancer (464 300; 95% UI = 399 900, 538 600), oesophageal cancer (242 500; 95% UI = 183 200, 288 500), non-melanoma skin cancer (212 700; 95% UI = 178 800, 251 900), and breast cancer (208 500; 95% UI = 168 100, 257 100). The biggest increase in the percentage changes in incident cases over the last 30 years occurred in testicular cancer (875.0%), non-melanoma skin cancer (797.5%), non-Hodgkin lymphoma (624.7%), and kidney cancer (604.0%). These cancers also had the highest AAPCs of all 29 cancers (Figure S1 and Table S2 in the **Online Supplementary Document**).

In 1990, stomach, lung, oesophageal, and liver cancers were the deadliest cancers for the Chinese population aged 55 years and above. However, lung cancer was the leading cancer of death in 2019, with the number of related deaths (668 890; 95% UI = 565 160, 782 430) being almost twice that of deaths due to stomach cancer (362 800; 95% UI = 305 290422 540). Moreover, the number of people dying from colon and rectal cancers had increased rapidly, surpassing liver cancer as the fourth most common cause of cancer-related death (Table S3 in the **Online Supplementary Document**).

#### Age and sex analyses

**Figure 2:**
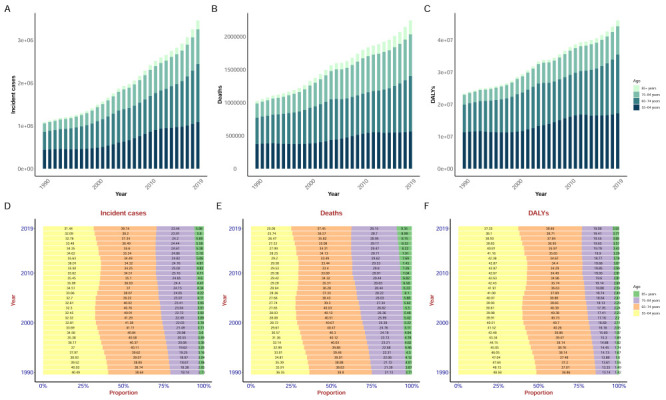
Incident cases, deaths and DALYs of the total 29 cancers within 4 age groups (55–64 years, 65–74 years, 75–84 years, >85 years) and the proportional incident cases, deaths, DALYs in the four age groups in China from 1990 to 2019. **Panel A.** Incident cases. **Panel B.** Deaths. **Panel C.** DALYs. **Panel D.** The proportional incident cases. **Panel E.** The proportional deaths. **Panel F.** The proportional DALYs. Estimates are for populations aged 55 years and above, both sexes in China. DALY – disability-adjusted life-year.

The number of incident cases, deaths, and DALYs increased from 1990 to 2019 overall and in the four age groups separately (55–64, 65–74, 75–84, and >85 years). Over time, the proportion of cases, deaths, and DALYs in older adults gradually increased. In the sub-analyses, incident cases, deaths, and DALYs were the highest in the 65–74 age group, while lung, stomach, oesophageal, colorectal, liver, and pancreatic cancers consistently caused the highest burden in the all four age groups (55–64, 65–74, 75–84, and >85 years) (**Figure 2**, Panels A–F; Figure S2 in the **Online Supplementary Document**).

**Figure 3:**
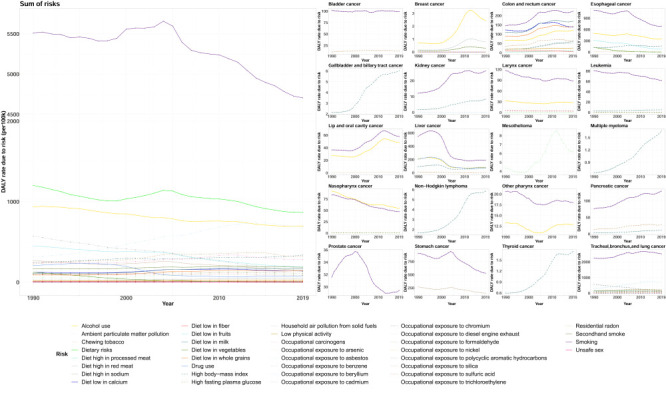
The total 20 cancers DALY rate due to risks and respective 20 cancers DALY rate due to risks of in men. Estimates are for populations aged 55 years and above among men in China. Rates are reported per 100 000 person-years. DALY – disability-adjusted life-year.

**Figure 4:**
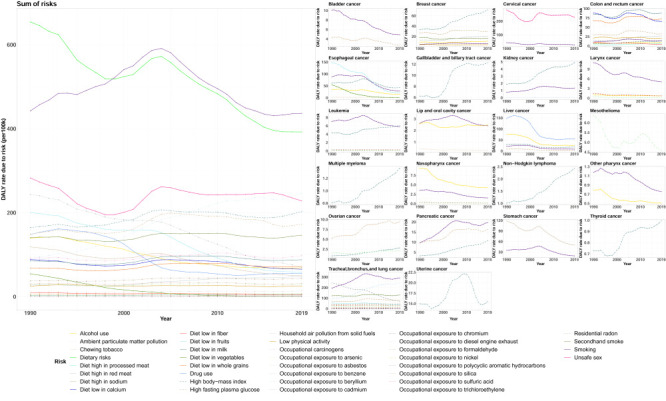
The total 22 cancers DALY rate due to risks and respective 22 cancers DALY rate due to risks of in women. Estimates are for populations aged 55 years and above among women in China. Rates are reported per 100 000 person-years. DALY – disability-adjusted life-year.

Analysing China's population aged 55 years and above by sex, we found that the incident cases and rates; DALYs and DALY rates; and deaths and death rates in men were generally higher than those in women. We observed a clear converse trend only in the incidence rate and DALY rate of breast cancer and incidence rate of thyroid cancer in women. Smoking was a clear risk factor for several cancers (larynx cancer, other pharynx cancer, bladder cancer, lip and oral cavity cancer, oesophageal cancer, nasopharynx cancer, and stomach cancer) for which higher DALY rates and incidence rates were observed among men. Likewise, the DALY rate and incidence rate of most cancers showed higher increases annually in men compared to women. We likewise noted that the percentage changes of incident cases, deaths, DALYs in most cancers increased more in men than women (**Figure 3**, **Figure 4**; Figures S3–4 and Tables S4–6 in the **Online Supplementary Document**).

Breast cancer was particularly prominent in women aged 55 years and above, with an increase in incidence rate from 50.6 (95% UI = 42.4, 59.4) in 1990 to 112.4 (95% UI = 90.1, 139.7) in 2019. In this population, the number of breast cancer incident cases in the population in 2019 at 203 300 (95% UI = 163 000, 252 600), making it the second highest of all cancers (after lung cancer), while its DALYs were the fourth highest at 1 575 000 (95% UI = 1 294 300, 1 904 200) (Figure S3 and Tables S4–6 in the **Online Supplementary Document**).

#### Risk analysis by sex

The most prominent risk factors in both men and women were smoking and dietary risk. The risk due to smoking was particularly acute among men and was also the highest risk factor for many cancers, such as lung cancer, stomach cancer, oesophageal cancers, liver cancer, and colon and rectum cancer, among others. In China, the DALY rate caused by dietary risks decreased each year in both sexes, the most prominent among them being diet low in fruits, diet low in milk, diet low in whole grains, diet low in calcium, and diet high in sodium. Ambient particulate matter pollution was the fourth highest risk factor for men and the third highest risk factor for women; it was also one of the two highest risk factors for lung cancer in both sexes, alongside smoking. Alcohol use in men was the third largest risk factor, and its overall harm was decreasing year by year. Unsafe sex was the fourth leading risk factor for women, mainly derived by cervical cancer. High fasting plasma glucose and high body-mass index were also important risk factors, lower only than alcohol use in men and unsafe sex in women. In both men and women, occupational carcinogens contributed less to cancer than smoking, dietary risks, ambient particulate matter pollution, alcohol use, high body-mass index, and high fasting plasma glucose, and their main effect occurred in lung cancer (**Figure 3**, **Figure 4**; Table S7–8 in the **Online Supplementary Document**).

#### Comparative analysis of China, the USA, and Western Europe

In the past 30 years, the total DALYs and deaths of the 29 cancers in China were far higher than those in the USA and Western Europe. However, the total age-standardised DALY rates and ASDRs were similar between the three regions, while the AAPCs of the age-standardised DALY rates and ASDRs showed similar downward trend from 1990 to 2019. The USA had the highest incident cases and ASIR of all 29 cancers among these three regions; however, this was mainly driven by non-melanoma skin cancer. The AAPC of ASIRs indicated that the growth rate of ASIR in China was between that of the USA and Western Europe (**Table 1**; Figure S3 and S5, Table S2–3 in the **Online Supplementary Document**).

**Figure 5:**
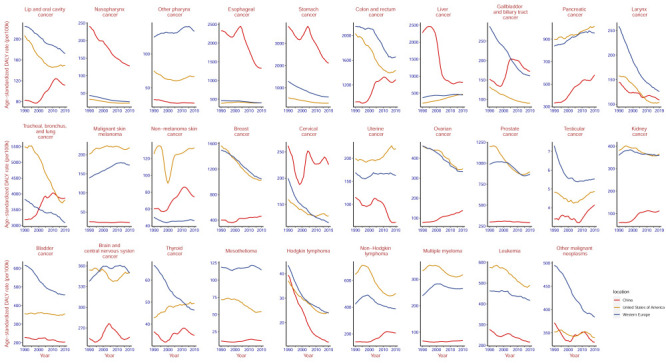
The change trend of age-standardised DALY rate in 29 cancers among China, United States, Western Europe from 1990 to 2019. Estimates are for populations aged 55 years and above. Rates are reported per 100 000 person-years. DALY – disability-adjusted life-year.

Compared to the USA and Western Europe, while the age-standardised DALY rates, ASDRs, and ASIRs of liver, nasopharyngeal, oesophageal, stomach, and cervical cancers in China were high in 2019, they had declined dramatically from 1990, except for the rates for cervical cancer (**Figure 5**; Figure S6–7 in the **Online Supplementary Document**).

Notably, the age-standardised DALY rates, ASDRs, and ASIRs of lung cancer and colon and rectum cancer in China increased in 1990–2020, which was in stark contrast to the USA and Western Europe. However, lung and colorectal cancer were also the important problem in USA and Western Europe. In 2019, the age-standardised DALY rates of lung cancer and of colon and rectum cancer were 3800.8 (95% UI = 3593.5–3977.9) and 1435.3 (95% UI = 1340.9–1514.7) in USA, while they were 3086.1 (95% UI = 2889.6–3256.8) and 1653.7 (95% UI = 1515.6–1765.7) in Western Europe, respectively (**Figure 5**, **Table 1**; Figure S6–7 in the **Online Supplementary Document**).

## DISCUSSION

The incident cases of 29 cancers in Chinese people above 55 years of age in 2019 increased 3-fold compared to 1990, while the number of DALYs and deaths almost doubled. This increase in the cancer burden undoubtedly brought more challenges to China's health care system. With the gradual change of China's ageing population, the proportion of elderly people is gradually increasing, and the number of cancer cases is likely to follow [6]. Our findings that the cancer burden in China is focussed primarily in the elderly is evidence of this trend.

In 2019, lung, stomach, oesophageal, colon and rectum, liver, and pancreatic cancer were the top six cancers with the highest burden among people aged 55 years and above in China.

We observed a significant, rapid growth of lung cancer in the elderly in China, with a strong increasing trend in the incidence rate; smoking and ambient particulate matter pollution acted as leading risk factors and resulted in a high burden.

Although the Chinese government introduced its first tobacco control law in the early 1990s and signed the World Health Organization Framework Convention on Tobacco Control in 2005 [22], these efforts had little success in the Chinese population. The prevalence of smoking among men in China was almost 50% in 2013 and was difficult to bring down. Noteworthy, the smoking prevalence of adolescent aged 15–25 years in China increased from 8.3% in 2003 to 12.5% in 2013 [23]. If the prevalence of smoking remains high, the prevalence of lung cancer will not be effectively curbed.

The Chinese economy achieved great progress over the past decades. However, the significant use of coal, the production of industrial waste, the increase in the use of motor vehicles, and the rapid development of cities all added to the ambient air pollution in China, making it one of the countries with the highest air pollution in the world [24,25]. To deal with this problem, the State Council of China issued the Air Pollution Prevention and Control Action Plan in 2013 [26]. Measures such as pollution end treatment, energy structure transformation, energy and climate policies, and economic structure changes had proved to be effective in achieving a downward turning point in the risk of air pollution-related deaths in China [27]. Between 2013 and 2017, the average annual concentration of particulate matter 2.5 decreased by 33.3%; however, the particulate matter 2.5 concentration in areas where most people (81%) lived still fell short of World Health Organization (WHO) least stringent interim air quality target (35 μg/m^3^) [28].

Like lung cancer, the incidence rate and DALY rate of colon and rectum cancer and pancreatic cancer in 2019 significantly increased compared to 1990. Colon and rectum cancer was thought to be closely associated with socioeconomic shifts, as it is closely related to life habits [29]. The high intake of animal origin foods, obesity, smoking, and alcohol use in high-income areas were associated with colon and rectum cancer, as well as pancreatic cancer [30]. Studies have shown that the mortality and incidence of colorectal cancer were on the rise in low-income countries; meanwhile, morbidity increased and mortality were decreasing in middle-income countries, while only a few high-income countries showed decreases of both mortality and morbidity. However, high-income countries tend to have higher mortality and morbidity than others [31,32]. This consistent with our findings that, although the burden of colorectal cancer in USA and Western Europe were higher than China, it had been declining over a 30-year period, while the burden of colorectal cancer in China was gradually rising.

Encouragingly, we found that the incidence and DALY rates of stomach, liver and oesophageal cancers had decreased significantly over the 30-year period. The control effect of liver cancer was the most prominent. In the past, hepatitis B and hepatitis C virus infection were the most important risk factor for liver cancer in China; for example, 80% of liver cancer in China was caused by hepatitis B virus infection [33]. To control the epidemic of hepatitis B, the Chinese government had made great efforts to vaccinate newborns and infants. Timely birth vaccination coverage increased from 22.2% in 1992 to 95.6% in 2015, and coverage of three doses of hepatitis B for infants increased from 30.0% to 99.6%, China had changed from highly endemic of hepatitis B virus infection to intermediate endemic area [34]. Moreover, hematological examination and imaging examination of liver cancer also played important roles in reducing the burden of liver cancer [33]. If reasonable comprehensive interventions could be taken, study predict that by 2050, liver cancer due to hepatitis B virus infection in China will be reduced by half [35].

Stomach cancer and esophageal cancer are both upper gastrointestinal cancers. Early diagnosis of upper gastrointestinal cancer was crucial for treatment, the survival rate of early diagnosis was 85%, and less than 10% of late diagnosis [36]. China launched the Upper Gastrointestinal Cancer Early Detection program in 2012 [37]; afterwards, a multicentre cohort study showed the 23% and 57% reduction in upper gastrointestinal cancer incidence and mortality, respectively, in the screening group compared to the control group [36]. However, compared with the national screening in Japan and South Korea, China's upper gastrointestinal cancer screening started late and was limited to key areas due to economic costs, doctor operation, and equipment [37]. While expanding screening coverage, focussing on reducing risk factors in life, such as *Helicobacter pylori* infection, smoking, alcohol consumption, and low fruit intake [38] will further reduce the incidence and mortality of upper gastrointestinal cancers.

We found that the development of breast cancer in women should be taken seriously. The incidence of breast cancer was highest in economically developed regions. For example, risk factors associated with rapid economic growth, such as obesity caused by a high-fat, high-calorie diet, less physical activity, a decline in breastfeeding, and a later age of first birth, led to a breast cancer epidemic in China. We found the highest incidence of breast cancer in China among the population aged 55–60 years; however, this high incidence was slowly shifting towards older populations, approaching the in high-income regions, where the highest incidence was observed in the >70-year-old old population [39–42]. Moreover, the stage I detection rate of breast cancer in China was 13.5%, much lower than the 50.5% in the USA [43], resulting in poor prognosis and post-cancer survival.

The burden of cancer was higher among men than women, a gap which was gradually increasing. Smoking was the most significant risk factor among men for the cancers where we observed the largest disparity in burden between men and women. The prevalence of smoking among men (about 50%) in China far exceeded that of smoking among women (about 3%) [23]. Reducing the smoking rate among Chinese residents, especially among men, is an urgent need and could help reduce the cancer burden in China in the future. Besides, reducing alcohol consumption also needs to be considered, as it was the third largest risk factor for cancer among Chinese men aged 55 years and above. However, the cancer gap between men and women may have more to do with inherent biological differences than with life factors. Physiologically, progesterone and oestrogen are believed to reduce the risk of certain cancers, while higher testosterone levels were thought to be linked to the development of certain cancers. In terms of immunity, women have a stronger immune response to hepatitis B, human papillomavirus, and also have tumour suppressor genes on the X chromosome [44].

Dietary risk management is also important in the prevention of cancer. We found that diet low in fruits, milk, whole grains and calcium, and diet high in sodium were major dietary risks. The modern diet of China’s population had been affected by various factors; for example, the rapid economic development led to the adoption of unhealthy diets as the general population consumed foods high in calories and fat [42]. Likewise, residents of China's rural or relatively poor areas did not intake sufficient fruit and protein, while some negative eating habits, such as the consumption of smoked meat and pickled food, led to high salt and sodium nitrite intake [45]. However, the dietary structure of Chinese residents was becoming more balanced, and the impact of dietary risk on cancer was decreasing each year. National health authorities need to provide dietary guidelines to the public to help the make reasonable choices about healthy diets.

We also found that, due to its huge population base, the number of DALYs caused by cancers in China was much higher than that of Western Europe or the USA. However, the age-standardised DALY rates in the three regions were similar. The cancers (liver, nasopharyngeal, oesophageal, stomach, and cervical cancers) for which we observed a higher burden in China than in the USA and Western Europe were all included in China's national cancer screening programmes [46]. We can see the ASRs of DALY, incidence, and death for all cancers except for cervical cancer declined significantly. Since 2005, the Chinese government launched four major public health programmes with the financial support of the central government, to gradually promote cancer screening from areas with high incidence to non-high-incidence areas. Eight common cancers were included in the cancer screening program – lung, stomach, colorectal, oesophageal, liver, breast, cervical, and nasopharyngeal cancers [46].

The rapid decline from 1990 to 1999 and rapid increase after 1999 for the incidence, mortality, and DALY rates of cervical cancer were unexpected. This may be due to the limited level of medical treatment in the past resulting in cervical cancer cases not being screened, leading to underestimations of the true disease burden of cervical cancer and bias in the data. A 2010 study even showed that only about 21% of Chinese women had received the Papanicolaou test for cervical cancer screening in their lifetime [47]. In 2019, screening for cervical cancer and breast cancer in China was expanded nationwide; by 2030, the Chinese government aims to achieve a 70% cervical cancer screening rate [48,49].

The changes of cancer in China in the past decades had been influenced by many factors. These include environmental pollution brought by economic development, the change of residents' living habits, population ageing, the improvement of medical standards, and the improvement of people's medical availability, among others. The significant increase in the burden of lung cancer, colon and rectum cancer, and breast cancer demonstrate the negative impact of economic development on people's health in China. Meanwhile, the decrease in the incidence and DALY rates of more typical cancers such as liver cancer, stomach cancer, and oesophageal cancer suggest that a series of measures taken by the Chinese government in the past to target key cancers had achieved results. China has been focussing more on screening of key cancers, leading to the inclusion of eight key cancers in the national cancer screening programmes. In the future, more free cancer screening in more non-key areas will further curb the development of cancer; however, this requires comprehensive consideration of economic cost, medical system, and other aspects. Expanding vaccination against cancer-associated viruses is also key to controlling cancer. The popularisation of health education for the public will also play an important role in prevention efforts, guiding the public to adhering to healthy lifestyles, such as discouraging people from smoking or drinking, encouraging them towards moderate physical activity to avoid obesity, and guiding them to adopt a reasonable diet structure. We also noted that testicular cancer, non-melanoma skin cancer, non-Hodgkin lymphoma, and kidney cancer increased rapidly, necessitating more attention towards these cancers. In general, despite successes in China's cancer control in the past, the current circumstances of cancer in China are still concerning, suggesting a need to advence the country's cancer prevention and control efforts. Government agencies should formulate and adjust public health cancer prevention policies according to the evolving trend of the current cancer spectrum, and should implement of cancer-related policies as soon as possible.

### Limitations

This study had several limitations. First, the GBD database includes relevant data for each country and territory as a unit; consequently, provincial and urban and rural data were not available. China is a large country with diverse terrain and there are significant differences in cancer characteristics between different provinces. Meanwhile, as a developing country, medical availability and cancer prevalence characteristics between urban and rural areas differ in China. Obtaining provincial, urban, and rural data could help us better understand the characteristics of cancer in China. Second, the early data in the study may be limited by the level of screening available at that point in time and may not accurately estimate the true level of cancer. This means that the comparisons between the USA, Western Europa, and China, may be biased due to the different cancer screening levels or screening standards in the three regions. These may affect the quality of some of the data and analysis. However the large number of cancers included in the study, the large time span, and the variety of analysis angles mean that our data are still useful for informing public health decisions.

## CONCLUSIONS

In 2019, the number of cancer cases among people aged 55 years and above in China increased 3-fold compared to 1990, while the number of DALYs and deaths almost doubled. The burden of cancer was also gradually shifting towards the older population. It was also significantly higher for men than for women – a gap which was widening over time. The incidence and DALY rates of stomach, oesophageal, and liver had decreased significantly from 1990. However, due to the negative impact of the country's economic development, lung cancer, colon and rectum cancer, breast cancer, and pancreatic cancer were becoming increasingly prevalent in the population; we therefore call for a balance between economic development and people's health. Smoking was the most significant risk factor for cancer in people aged 55 years and above in China, especially in men, and effective control of smoking should be taken for cancer prevention and control in the future. Since dietary risk was the second largest contributor to the cancer burden, it is equally urgent to guide people to adopt a healthy diet and abandon bad eating habits. Similar to the USA and Western Europe, the overall ASIR of cancer in China had increased while the age-standardised DALY rate had decreased, reflecting population ageing and the improvement of medical standards. Overall, the cancer burden in China remains serious, necessitating more efforts such as improving the health environment, improving the accessibility of medical care to the masses, expanding the scope of cancer screening, identifying key populations, assisting people in adopting a healthy lifestyle, and monitoring the prevalence of cancer.

## Additional material


Online Supplementary Document
