# Supplementary material for: Cancer burden and risk in the Chinese population aged 55 years and above: A systematic analysis and comparison with the USA and Western Europe
Source: J Glob Health. 2024 Jan 26;14:04014. doi: 10.7189/jogh.14.04014 (PMC10810324; doi:10.7189/jogh.14.04014)
Supplement: Online Supplementary Document [file jogh-14-04014-s001.pdf]

Legends of Online Supplementary Document

Table S1 List of International Classification of Diseases (ICD) codes mapped to the Global Burden of Disease cause list for neoplasms

Note: ICD= International Classification of Diseases.

Table S2 The incident cases and ASIR of 29 cancers among China, United States and Western Europe in 1990 and 2019, and the AAPC of ASIR from 1990 to 2019

Note: Estimates are for populations aged 55 years and above, both sexes combined. All malignant cancers are the sum of the following 29 cancers. Rates are reported per 100000 person-years. Cancer types are listed alphabetically. Other malignant neoplasms are cancers without a detailed GBD cause separately listed. UI=uncertainty interval. ASIR=age-standardized incidence rate. AAPC=average annual percentage change. CI = confidence interval. UI = uncertainty intervals.

Table S3 The death cases and ASDR of 29 cancers among China, United States and Western Europe in 1990 and 2019, and the AAPC of ASDR from 1990 to 2019

Note: Estimates are for populations aged 55 years and above, both sexes combined. All malignant cancers are the sum of the following 29 cancers. Rates are reported per 100000 person-years. Cancer types are listed alphabetically. Other malignant neoplasms are cancers without a detailed GBD cause separately listed. UI=uncertainty interval. ASDR=age-standardized death rate. AAPC=average annual percentage change. CI = confidence interval. UI = uncertainty intervals.

Table S4 The DALYs and DALY rate of 29 cancers among men and women over 55+ years old in China in 1990 and 2019, and percentage change in DALYs, annual rate of change in DALY rate from 1990 to 2019

Note: Estimates are for populations aged 55 years and above among men and women in China. Rates are reported per 100000 person-years. Cancer types are listed alphabetically. Other malignant neoplasms are cancers without a detailed GBD cause separately listed. DALYs=disability-adjusted life-years. UI=uncertainty interval.

Table S5 The death cases and death rate of 29 cancers among men and women over 55+ years old in China in 1990 and 2019, and percentage change in death cases, annual rate of change in death rate from 1990 to 2019

Note: Estimates are for populations aged 55 years and above among men and women in China. Rates are reported per 100000 person-years. Cancer types are listed alphabetically. Other malignant neoplasms are cancers without a detailed GBD cause separately listed. UI=uncertainty interval.

Table S6 The incident cases and incident rate of 29 cancers among men and women over 55+ years old in China in 1990 and 2019, and percentage change in incident cases, annual rate of change in incidence rate from 1990 to 2019

Note: Estimates are for populations aged 55 years and above among men and women in China. Rates are reported per 100000 person-years. Cancer types are listed alphabetically. Other malignant neoplasms are cancers without a detailed GBD cause separately listed. UI=uncertainty interval.

Table S7 The DALYs and DALY rate in the risks for 20 cancers among men in 1990 and 2019, and percentage change in DALYs, annual rate of change in DALY rate from 1990 to 2019

Note: Estimates are for populations aged 55 years and above among men in China. Rates are reported per 100000 person-years. Cancer types and risks are listed alphabetically. DALY=disability-adjusted life-year, UI=uncertainty interval.

Table S8 The DALYs and DALY rate in the risks for 22 cancers among women in 1990 and 2019, and percentage change in DALYs, annual rate of change in DALY rate from 1990 to 2019

Note: Estimates are for populations aged 55 years and above among women in China. Rates are reported per 100000 person-years. Cancer types and risks are listed alphabetically. DALY=disability-adjusted life-year, UI=uncertainty interval.

Figure S1 The changing trend of incidence rate (A) and the rank of incident cases (B) for 29 cancers in China from 1990 to 2019

Note: Estimates are for populations aged 55 years and above, both sexes in China. Rates are reported per 100000 person-years.

Figure S2 Incident cases, deaths and DALYs (A, B, C) of 29 cancers in 4 age groups (55−64 years, 65−74 years, 75−84 years, 85+ years) and the proportional incident cases, deaths, DALYs (D, E, F) in the four age groups in China in 2019

Note: Estimates are for populations aged 55 years and above, both sexes in China. DALY = disability-adjusted life-year.

Figure S3 Incident cases, DALYs (A, B) and the proportional incident cases, DALYs (C, D) of 29 cancers among men and women in China in 2019

Note: Estimates are for populations aged 55 years and above among men and women in China. DALY=disability-adjusted life-year.

Figure S4 Ratio of men to women incidence rate (A) and ratio of men to women DALY rate (B) of 24 cancers (the cancers can affect both men and women) in China from 1990 to 2019

Note: Estimates are for populations aged 55 years and above. DALY=disability-adjusted life-year.

Figure S5 Incident cases (A), death cases (C), DALYs (E) and the proportional incident cases (B), proportional deaths (D), proportional DALYs (F) of 29 cancers among China, United States, Western Europe in 2019

Note: Estimates are for populations aged 55 years and above. DALY=disability-adjusted life-year.

Figure S6 The change trend of ASIR in 29 cancers among China, United States, Western Europe from 1990 to 2019

Note: Estimates are for populations aged 55 years and above. Rates are reported per 100000 person-years. ASIR=age-standardized incidence rate.

Figure S7 The change trend of ASDR in 29 cancers among China, United States, Western Europe from 1990 to 2019

Note: Estimates are for populations aged 55 years and above. Rates are reported per 100000 person-years. ASDR=age-standardized death rate.

Table S1 List of International Classification of Diseases (ICD) codes mapped to the Global Burden of Disease cause list for neoplasms

Cancer type	ICD10	ICD9
**Lip and oral cavity cancer**	C00-C07, C08-C08.9, Z85.81-Z85.810	140-145.9, V76.42
**Nasopharynx cancer**	C11-C11.9	147-147.9
**Other pharynx cancer**	C09-C10.9, C12-C13.9	146-146.9, 148-148.9
**Esophageal cancer**	C15-C15.9, Z85.01	150-150.9
**Stomach cancer**	C16-C16.9, Z12.0, Z85.02-Z85.028	151-151.9, 209.23, V10.04
**Colon and rectum cancer**	C18-C19.0, C20, C21-C21.8, Z12.1-Z12.13, Z85.03-Z85.048, Z86.010	153-154.9, 209.1-209.17, V10.05-V10.06, V76.41, V76.5-V76.52
**Liver cancer**	C22-C22.4, C22.7-C22.9, Z85.05	155-155.9, V10.07
**Gallbladder and biliary tract cancer**	C23, C24-C24.9	156-156.9
**Pancreatic cancer**	C25-C25.9, Z85.07	157-157.9
**Larynx cancer**	C32-C32.9, Z85.21	161-161.9, V10.21
**Tracheal, bronchus, and lung cancer**	C33, C34-C34.92, Z12.2, Z80.1-Z80.2, Z85.1-Z85.20	162-162.9, 209.21, V10.1-V10.20, V16.1-V16.2, V16.4-V16.40
**Malignant skin melanoma**	C43-C43.9, Z85.82-Z85.828	172-172.9
**Non-melanoma skin cancer**	C44.01-C44.99	173-173.99
**Breast cancer**	C50-C50.629, C50.8-C50.929, Z12.3-Z12.39, Z80.3, Z85.3, Z86.000	174-175.9, V10.3, V16.3
**Cervical cancer**	C53-C53.9, Z12.4, Z85.41	180-180.9, V10.41, V72.32
**Uterine cancer**	C54-C54.3, C54.8-C54.9, Z85.42, Z86.001	182-182.9
**Ovarian cancer**	C56-C56.2, C56.9, Z80.41, Z85.43	183-183.0, 183.8-183.9, V10.43, V16.41
**Prostate cancer**	C61-C61.9, Z12.5, Z80.42, Z85.46	185-185.9, V10.46, V16.42, V76.44
**Testicular cancer**	C62-C62.92, Z80.43, Z85.47-Z85.48	186-186.9, V10.47-V10.48, V16.43
**Kidney cancer**	C64-C64.2, C64.9-C65.9, Z80.51, Z85.52-Z85.54	189-189.1, 189.5-189.6, 209.24

**Bladder cancer**	C67-C67.9, Z12.6-Z12.79, Z80.52, Z85.51	188-188.9, V10.51, V16.52, V76.3
**Brain and central nervous system cancer**	C70-C70.1, C70.9-C72.9, Z85.841-Z85.848, Z86.011	191-191.9
**Thyroid cancer**	C73, Z85.850	193-193.9
**Mesothelioma**	C45-C45.2, C45.7, C45.9	/
**Hodgkin lymphoma**	C81-C81.49, C81.7-C81.79, C81.9-C81.99, Z85.71-Z85.72	201-201.98, V10.72
**Non-Hodgkin lymphoma**	C82-C85.29, C85.7-C86.6, C96-C96.9	200-200.9, 202-202.98
**Multiple myeloma**	C88-C90.32	203-203.9
**Leukemia**	C91-C93.7, C93.9-C95.2, C95.7-C95.92, Z80.6, Z85.6	204-208.92, V10.59-V10.69, V16.6
**Other malignant neoplasms**	C75.90-C75.92, D00-D24.9, D26.0-D39.9, D4-D49.9, E34.0, K51.4-K51.419, K62.0-K62.3, K63.5, N60-N60.99, N84.0- N84.1, N87-N87.9	209.4-209.57, 209.61, 209.63-209.67, 210.0-217.8, 219-237.6, 237.70-237.72, 237.9-239.9, 569.0, 610- 610.9, 622.1-622.2, 622.7

Note: ICD= International Classification of Diseases.

Table S2 The incident cases and ASIR of 29 cancers among China, United States and Western Europe in 1990 and 2019, and the AAPC of ASIR from 1990 to 2019

Cause	Incident cases*1000 in 1990 (95% UI)		
	China	United States	Western Europe
All malignant cancers	1086.7 (899.9-1275.9)	2018.9 (1740.8-2300.7)	1508.8 (1390.6-1613.5)
Bladder cancer	19.3 (16.9-22)	24.3 (23.1-25)	80.6 (77.8-82.8)
Brain and central nervous system cancer	15.6 (12.8-20.9)	9.5 (8.2-11.4)	17 (15-21.8)
Breast cancer	37.5 (31.5-44)	131.8 (125.6-135.9)	154.7 (147.4-159.5)
Cervical cancer	18.3 (14.6-29.9)	6.5 (5.9-6.8)	14.5 (13.3-15.3)
Colon and rectum cancer	72.5 (64.3-81.6)	135.7 (129.3-139.5)	204.7 (196.2-209.8)
Esophageal cancer	143.5 (92.4-166.6)	10.3 (9.9-10.6)	22.6 (21.9-23.1)
Gallbladder and biliary tract cancer	9.7 (8-16.1)	6.4 (5.3-6.8)	20.8 (16.4-22)
Hodgkin lymphoma	2.6 (1.4-3.4)	2.5 (2-2.7)	3.8 (3.1-4.2)
Kidney cancer	5 (4.3-5.8)	24.1 (23.2-24.9)	30.7 (29.5-31.6)
Larynx cancer	10.6 (9.1-12.2)	9.8 (9.5-10)	20.6 (20-21.2)
Leukemia	18.5 (15.7-22.3)	26 (24.5-26.9)	38.6 (36.7-39.8)
Lip and oral cavity cancer	7.5 (6.7-8.5)	16.7 (16.1-17.2)	24.1 (23.3-25)
Liver cancer	137.2 (115.9-165.4)	5.6 (5.4-5.7)	17.8 (17.2-18.3)
Malignant skin melanoma	1.7 (1.3-2.5)	19.5 (14.6-25.3)	19.2 (15.8-26.3)
Mesothelioma	0.7 (0.6-1)	2.5 (2.3-2.7)	7 (6.4-7.7)
Multiple myeloma	4.6 (3.8-6.4)	12.4 (10.5-12.9)	17.1 (15.7-19.4)
Nasopharynx cancer	15.1 (12.9-17.3)	1.1 (1.1-1.2)	3.1 (2.9-3.2)
Non-Hodgkin lymphoma	8.5 (7.3-9.7)	32.2 (29.8-34.6)	37.4 (34.7-40.2)
Non-melanoma skin cancer	23.7 (19.2-29.2)	1074.2 (895.2-1305.6)	200.2 (177.9-227)
Other malignant neoplasms	26.6 (22.9-30.5)	23.9 (22.2-25.7)	41.3 (35.8-43.5)
Other pharynx cancer	2.1 (1.8-2.4)	5.2 (5-5.4)	7.6 (7.4-7.9)
Ovarian cancer	5.2 (4-8.2)	15.1 (13.6-15.7)	27 (24.5-28)

**Pancreatic cancer**	20 (17.4-22.4)	26 (24.6-26.8)	44 (42.1-45.1)
**Prostate cancer**	24.9 (19-30)	175.3 (120-193.7)	136.8 (103.5-166.3)
**Stomach cancer**	241 (211.8-272)	23.6 (22.4-24.3)	83.7 (79.8-85.8)
**Testicular cancer**	0.4 (0.4-0.5)	0.7 (0.6-0.8)	1.5 (1.1-1.8)
**Thyroid cancer**	3.8 (3.2-4.9)	6.6 (6.3-6.9)	10.5 (9.8-11)
**Tracheal, bronchus, and lung cancer**	197.7 (170.7-224.7)	163.4 (157.8-166.9)	188.8 (183.7-191.9)
**Uterine cancer**	12.6 (9.9-15.4)	27.9 (26.7-28.8)	33.2 (31.8-34.4)

**Cause**	**Incident cases*1000 in 2019 (95% UI)**		
	**China**	**United States**	**Western Europe**
**All malignant cancers**	3458.9 (2840.8-4127.2)	5398.4 (4772.4-6138.4)	2585.2 (2181-3072.2)
**Bladder cancer**	81.5 (68-96.7)	45.7 (39.1-53.6)	128.9 (110.9-148.2)
**Brain and central nervous system cancer**	46.8 (34.8-56.5)	18.8 (13.5-22.5)	32.2 (19.6-39.9)
**Breast cancer**	208.5 (168.1-257.1)	193.5 (161.2-232)	248.6 (214.9-283.2)
**Cervical cancer**	48.2 (26.7-61.1)	9.7 (7.7-11.8)	15 (12.6-17.3)
**Colon and rectum cancer**	464.3 (399.9-538.6)	196.3 (170.6-225.6)	348.1 (302.3-393)
**Esophageal cancer**	242.5 (183.2-288.5)	20.8 (17.7-24.5)	36 (31.4-40.8)
**Gallbladder and biliary tract cancer**	33.2 (23.4-39.8)	8.8 (7.4-10.7)	22.1 (18.1-25.9)
**Hodgkin lymphoma**	4.4 (3.1-5.3)	4.1 (3.4-5.2)	6.1 (5.1-7.3)
**Kidney cancer**	35.2 (29.4-41.8)	50.6 (43.2-58.7)	63.5 (55.9-71.5)
**Larynx cancer**	37.1 (30.2-44.9)	14.4 (12-17.2)	20.9 (18-24.2)
**Leukemia**	62.6 (51.3-73.7)	45.4 (38.7-53)	88.9 (77-101.4)
**Lip and oral cavity cancer**	33.6 (28.1-39.9)	25.8 (22.2-30.1)	32.8 (28.3-37.7)
**Liver cancer**	139.8 (117.3-165)	24.1 (19.7-29)	41 (35.7-47.1)
**Malignant skin melanoma**	8.9 (5.6-11.3)	58.6 (40-74.3)	65.7 (39.2-78.4)
**Mesothelioma**	1.8 (1.5-2.2)	3.6 (3-4.2)	11.4 (9.9-12.8)
**Multiple myeloma**	14.7 (10.9-18.3)	25 (21.2-30.3)	36.2 (29.1-41.4)

**Nasopharynx cancer**	47.4 (38.8-57.2)	1.8 (1.5-2.1)	5.3 (4.5-6.1)
**Non-Hodgkin lymphoma**	61.6 (51.3-73.1)	54.2 (46.4-63)	78.7 (66.4-90.8)
**Non-melanoma skin cancer**	212.7 (178.8-251.9)	3813 (3455.5-4179.2)	332.7 (276.1-404)
**Other malignant neoplasms**	126 (101.7-150.4)	52.7 (44.7-61.2)	78.1 (64.8-90)
**Other pharynx cancer**	7.1 (5.9-8.5)	11.2 (9.3-13.4)	18.4 (15.7-21.5)
**Ovarian cancer**	25.9 (17.7-32.5)	21.1 (17.1-25.7)	32.9 (27.9-38.1)
**Pancreatic cancer**	94.6 (80.9-109.3)	53.9 (46.4-61.9)	86.3 (74.5-97.8)
**Prostate cancer**	145.5 (112.5-193.1)	293.6 (228.9-439.5)	314.7 (258.6-455)
**Stomach cancer**	496.4 (418-586)	28.1 (24.2-32.5)	78.2 (67.8-88)
**Testicular cancer**	3.9 (2.9-4.9)	1.5 (1.1-2)	2.3 (1.8-2.9)
**Thyroid cancer**	17.6 (14.6-21)	17.2 (14.6-19.9)	16.1 (14-18.4)
**Tracheal, bronchus, and lung cancer**	721.9 (608.6-847.3)	238 (206.9-274.7)	274.6 (240.9-310.3)
**Uterine cancer**	35.3 (27.4-51.3)	67.2 (55.4-80.7)	69.5 (60-79.2)
**Cause**	**ASIR in 1990 (95% UI)**		
	**China**	**United States**	**Western Europe**
**All malignant cancers**	802.9 (658.7-953.8)	3606.2 (2965.6-4261)	1490.6 (1342.9-1631.6)
**Bladder cancer**	16.2 (13.9-18.6)	43.1 (40.6-45)	78.8 (74.5-82.6)
**Brain and central nervous system cancer**	11 (8.8-15.1)	17.5 (14.7-21.1)	17.3 (14.8-22)
**Breast cancer**	26.2 (21.9-31)	242.8 (226.9-255.8)	155.9 (145.5-165)
**Cervical cancer**	13 (10.2-21.3)	12.3 (10.7-13.2)	14.8 (13.2-16.1)
**Colon and rectum cancer**	55.9 (49.2-63.5)	240.6 (227.2-250.2)	199.6 (188.5-208)
**Esophageal cancer**	106 (68.4-124.4)	18.8 (17.9-19.6)	22.6 (21.6-23.5)
**Gallbladder and biliary tract cancer**	7.8 (6.4-12.8)	11.2 (9.2-12.3)	20.1 (15.6-22)
**Hodgkin lymphoma**	1.9 (1-2.5)	4.6 (3.6-5.2)	3.8 (3-4.3)
**Kidney cancer**	3.7 (3.1-4.3)	44.4 (42.1-46.5)	30.6 (28.6-32.7)
**Larynx cancer**	7.7 (6.5-8.9)	18.3 (17.4-19.2)	21.2 (20-22.5)

**Leukemia**	13.3 (11.1-16.5)	46.3 (43-48.6)	38.2 (35.5-40.4)
**Lip and oral cavity cancer**	5.7 (4.9-6.5)	30.8 (29.1-32.4)	24.7 (22.9-26.5)
**Liver cancer**	96.9 (81-117.1)	10.1 (9.5-10.5)	17.6 (16.6-18.5)
**Malignant skin melanoma**	1.4 (1-2)	36.2 (26.3-46.6)	19.3 (15.4-26.9)
**Mesothelioma**	0.5 (0.4-0.8)	4.4 (4-4.8)	6.9 (6.1-7.8)
**Multiple myeloma**	3.3 (2.6-4.7)	22.1 (18.1-23.7)	16.8 (14.9-19.1)
**Nasopharynx cancer**	10.6 (8.9-12.5)	2.2 (2-2.3)	3.2 (2.9-3.6)
**Non-Hodgkin lymphoma**	6.3 (5.4-7.4)	57.1 (51.2-62.9)	36.7 (33-40.4)
**Non-melanoma skin cancer**	19.4 (15-25.1)	1903.2 (1463.7-2438)	197 (166.5-234.6)
**Other malignant neoplasms**	20 (17-23.7)	43.1 (39-47.1)	41.1 (34.6-44.7)
**Other pharynx cancer**	1.6 (1.3-1.8)	10 (9.4-10.6)	8 (7.5-8.5)
**Ovarian cancer**	3.8 (2.9-5.9)	27.6 (24.7-29.1)	27.1 (24.1-28.7)
**Pancreatic cancer**	14.9 (12.9-16.9)	46.2 (43.5-48.1)	43 (40.7-44.7)
**Prostate cancer**	20.8 (15.6-26.4)	309.3 (206.9-350)	131.4 (97.3-160.9)
**Stomach cancer**	178.7 (155.5-203.6)	41.5 (38.9-43.3)	81.2 (76.6-84.5)
**Testicular cancer**	0.3 (0.3-0.4)	1.4 (1.1-1.7)	1.6 (1.1-2.1)
**Thyroid cancer**	2.8 (2.3-3.6)	12.6 (11.6-13.6)	10.7 (9.7-11.8)
**Tracheal, bronchus, and lung cancer**	144.5 (124.4-165.4)	297 (285.4-305.3)	187.9 (181.6-193.1)
**Uterine cancer**	8.7 (6.6-11)	51.4 (47.9-54.3)	33.5 (31-36)

**Cause**	**ASIR in 2019 (95% UI)**		
	**China**	**United States**	**Western Europe**
**All malignant cancers**	1022.3 (825.1-1230.8)	5432.8 (4625.8-6376.3)	1631.2 (1328.8-1995.6)
**Bladder cancer**	24.9 (20.5-29.7)	45.7 (38-54.1)	78.4 (65.6-91.9)
**Brain and central nervous system cancer**	13.8 (10-16.9)	19.1 (13.5-23.5)	21.4 (13-27)
**Breast cancer**	59 (47.1-73)	197.9 (161.5-240.2)	163.7 (138.1-191.2)
**Cervical cancer**	13.7 (7.6-17.5)	10.1 (7.8-12.4)	10 (8.1-12)

**Colon and rectum cancer**	137.8 (116.8-160.5)	198 (167.7-230)	213.7 (182.2-245.4)
**Esophageal cancer**	72.5 (53.5-87.1)	21.2 (17.6-25.3)	23.1 (19.6-26.9)
**Gallbladder and biliary tract cancer**	10.3 (7.2-12.4)	8.8 (7.3-10.9)	12.9 (10.4-15.5)
**Hodgkin lymphoma**	1.3 (0.9-1.7)	4.1 (3.2-5.4)	3.9 (3.1-5)
**Kidney cancer**	10.3 (8.5-12.3)	51.7 (43.3-61)	40.9 (34.6-47.8)
**Larynx cancer**	10.7 (8.6-13)	14.8 (12.2-18)	14.4 (12.1-17.2)
**Leukemia**	18 (14.4-21.6)	45 (37.5-53.1)	54.4 (45.3-64)
**Lip and oral cavity cancer**	9.8 (8.1-11.8)	26.4 (22.1-31.1)	22.1 (18.4-25.9)
**Liver cancer**	40.7 (33.7-48.3)	24.8 (20-30.1)	26 (22-30.3)
**Malignant skin melanoma**	2.7 (1.6-3.5)	59.7 (40.3-78.2)	43 (25.6-52.9)
**Mesothelioma**	0.5 (0.4-0.7)	3.6 (3-4.3)	7.1 (6-8.3)
**Multiple myeloma**	4.3 (3.1-5.4)	25.2 (20.6-31.6)	22.1 (17.3-26.3)
**Nasopharynx cancer**	13.5 (10.7-16.8)	1.8 (1.5-2.2)	3.7 (2.9-4.5)
**Non-Hodgkin lymphoma**	18.1 (14.9-21.9)	53.8 (44.5-64)	46.8 (38.7-55.1)
**Non-melanoma skin cancer**	62.6 (49.1-78.1)	3824.3 (3320.4-4363.6)	203.9 (155-267.3)
**Other malignant neoplasms**	37.6 (29.7-45.2)	53 (44-62.3)	48 (39-56.8)
**Other pharynx cancer**	2.1 (1.7-2.5)	11.7 (9.5-14.1)	13.3 (10.9-16.1)
**Ovarian cancer**	7.4 (5-9.4)	21.5 (17-26.5)	21.2 (17.6-25.3)
**Pancreatic cancer**	28.3 (23.9-33)	54.2 (45.8-62.9)	52.4 (44.2-60.4)
**Prostate cancer**	43.7 (33.1-59.2)	298.5 (226.3-451.6)	200.4 (157.1-294.7)
**Stomach cancer**	147.2 (122.5-174.4)	28.1 (23.7-32.9)	46.8 (39.7-53.7)
**Testicular cancer**	1.1 (0.8-1.5)	1.7 (1.1-2.2)	1.7 (1.2-2.3)
**Thyroid cancer**	5.1 (4.1-6.1)	17.9 (14.9-21.1)	11.3 (9.5-13.2)
**Tracheal, bronchus, and lung cancer**	215.5 (179.9-253.2)	240.9 (205.4-279)	177.4 (152.2-203.2)
**Uterine cancer**	9.9 (7.5-14.2)	69.4 (56.2-84.6)	47.1 (39.4-55.3)
**Cause**		**AAPC (95% CI)**	

	China	United States	Western Europe
**All malignant cancers**	0.81 (0.65 to 0.98)	1.39 (1.3 to 1.48)	0.38 (0.3 to 0.47)
**Bladder cancer**	1.42 (1.19 to 1.66)	0.17 (-0.01 to 0.35)	-0.02 (-0.12 to 0.08)
**Brain and central nervous system cancer**	0.78 (0.59 to 0.97)	0.29 (0.16 to 0.42)	0.73 (0.57 to 0.89)
**Breast cancer**	2.72 (2.12 to 3.31)	-0.77 (-0.94 to -0.59)	0.16 (0.03 to 0.28)
**Cervical cancer**	0.16 (-0.23 to 0.54)	-0.71 (-0.99 to -0.44)	-1.28 (-1.45 to -1.1)
**Colon and rectum cancer**	3.13 (2.86 to 3.4)	-0.67 (-0.95 to -0.38)	0.19 (0.01 to 0.36)
**Esophageal cancer**	-1.34 (-1.56 to -1.12)	0.32 (0.26 to 0.38)	0.06 (0.01 to 0.12)
**Gallbladder and biliary tract cancer**	0.93 (0.78 to 1.08)	-0.87 (-1.06 to -0.68)	-1.52 (-1.62 to -1.41)
**Hodgkin lymphoma**	-1.24 (-1.53 to -0.94)	-0.33 (-0.51 to -0.16)	0.09 (-0.01 to 0.19)
**Kidney cancer**	3.55 (3.33 to 3.78)	0.45 (0.19 to 0.71)	1.01 (0.89 to 1.13)
**Larynx cancer**	1.13 (0.95 to 1.3)	-0.71 (-0.94 to -0.48)	-1.31 (-1.43 to -1.19)
**Leukemia**	1.02 (0.79 to 1.26)	-0.11 (-0.36 to 0.15)	1.23 (1.1 to 1.36)
**Lip and oral cavity cancer**	1.84 (1.68 to 2.01)	-0.48 (-0.6 to -0.36)	-0.39 (-0.54 to -0.23)
**Liver cancer**	-3 (-3.21 to -2.79)	3.14 (3.11 to 3.18)	1.36 (1.3 to 1.42)
**Malignant skin melanoma**	2.35 (2.1 to 2.61)	1.78 (1.53 to 2.03)	2.79 (2.61 to 2.96)
**Mesothelioma**	-0.01 (-0.22 to 0.2)	-0.7 (-0.86 to -0.55)	0.13 (0 to 0.26)
**Multiple myeloma**	0.85 (0.68 to 1.02)	0.45 (0.35 to 0.56)	0.96 (0.84 to 1.08)
**Nasopharynx cancer**	0.74 (0.52 to 0.97)	-0.63 (-0.76 to -0.5)	0.48 (0.38 to 0.58)
**Non-Hodgkin lymphoma**	3.67 (3.28 to 4.07)	-0.21 (-0.37 to -0.06)	0.87 (0.73 to 1.01)
**Non-melanoma skin cancer**	4.15 (3.92 to 4.38)	2.39 (2.26 to 2.52)	0.18 (0.09 to 0.26)
**Other malignant neoplasms**	2.17 (2.04 to 2.31)	0.74 (0.63 to 0.84)	0.54 (0.44 to 0.65)
**Other pharynx cancer**	0.97 (0.82 to 1.12)	0.64 (0.3 to 0.98)	1.78 (1.48 to 2.07)
**Ovarian cancer**	2.31 (2.09 to 2.53)	-0.85 (-1.1 to -0.59)	-0.96 (-1.04 to -0.88)
**Pancreatic cancer**	2.22 (1.95 to 2.49)	0.57 (0.28 to 0.86)	0.7 (0.56 to 0.84)
**Prostate cancer**	2.58 (2.35 to 2.8)	-0.11 (-0.22 to 0.01)	1.46 (1.34 to 1.59)

**Stomach cancer**	-0.69 (-0.9 to -0.48)	-1.34 (-1.6 to -1.08)	-1.9 (-2.06 to -1.73)
**Testicular cancer**	4.25 (3.78 to 4.73)	0.54 (0.37 to 0.71)	0.26 (0.01 to 0.5)
**Thyroid cancer**	2.04 (1.72 to 2.36)	1.23 (0.89 to 1.58)	0.15 (0.01 to 0.28)
**Tracheal, bronchus, and lung cancer**	1.33 (1.07 to 1.6)	-0.69 (-1.1 to -0.27)	-0.16 (-0.35 to 0.04)
**Uterine cancer**	0.38 (0.02 to 0.75)	0.97 (0.58 to 1.37)	1.17 (0.92 to 1.42)

Note: Estimates are for populations aged 55 years and above, both sexes combined. All malignant cancers are the sum of the following 29 cancers. Rates are reported per 100000 person-years. Cancer types are listed alphabetically. Other malignant neoplasms are cancers without a detailed GBD cause separately listed. UI=uncertainty interval. ASIR=age-standardized incidence rate. AAPC=average annual percentage change. CI = confidence interval. UI = uncertainty intervals.

Table S3 The death cases and ASDR of 29 cancers among China, United States and Western Europe in 1990 and 2019, and the AAPC of ASDR from 1990 to 2019

Cause	Deaths*100 in 1990 (95% UI)		
	China	United States	Western Europe
**All malignant neoplasms**	10255.9 (8495.6-12019.9)	4642.4 (4277.9-4849.5)	8532.2 (7949.3-8951.6)
**Bladder cancer**	148.2 (129.7-167)	116.3 (108.9-120.8)	355.2 (338.4-365.5)
**Brain and central nervous system cancer**	158.6 (129.5-212)	85.5 (73.7-103.1)	142.8 (127.3-185.7)
**Breast cancer**	236.3 (198.7-277.3)	375.5 (352.2-387.8)	710.6 (667.9-731.8)
**Cervical cancer**	163 (129.5-259.2)	39.8 (35.9-41.9)	98.4 (89.8-102.8)
**Colon and rectum cancer**	583 (514.6-653.1)	600.2 (563.5-620.7)	1212.7 (1148.3-1247.8)
**Esophageal cancer**	1494.3 (948.6-1736.8)	99.7 (95.9-102.7)	223.8 (215.7-228.7)
**Gallbladder and biliary tract cancer**	98.1 (80.9-161.9)	41.3 (33.9-43.7)	165.8 (130.3-174.6)
**Hodgkin lymphoma**	24.9 (13.2-32)	9.2 (7.5-10.2)	20.8 (16.9-23.1)
**Kidney cancer**	37.1 (32.1-42.6)	98.3 (93.7-101.8)	177.1 (169.3-182.4)
**Larynx cancer**	90.4 (77.5-103.3)	36.5 (35.2-37.4)	109.6 (106.4-112.3)
**Leukemia**	170.4 (146.2-204.4)	169.8 (159.7-175.7)	250.4 (237.4-257.3)
**Lip and oral cavity cancer**	51.5 (45.1-57.8)	50.7 (48.3-52.1)	97.3 (93.3-99.7)
**Liver cancer**	1417.3 (1212.5-1698.7)	55.3 (52.7-56.8)	180.9 (173.6-186)
**Malignant skin melanoma**	15.4 (11.2-23.1)	49 (36.8-64.3)	65.4 (54.7-91.7)
**Mesothelioma**	7.1 (5.5-10)	20.7 (19.3-22.2)	58.2 (53-64.4)
**Multiple myeloma**	42.2 (34.5-58.4)	94.8 (80.2-98.8)	126.7 (116.9-144.1)
**Nasopharynx cancer**	146.9 (126.6-168.6)	7.1 (6.8-7.3)	17.1 (16.5-17.7)
**Non-Hodgkin lymphoma**	88.6 (77.6-100)	181.2 (171.2-186.6)	214.3 (204.7-220.4)
**Non-melanoma skin cancer**	39.3 (34.4-44.1)	20.4 (18.9-21.1)	33.3 (30.1-34.7)
**Other malignant neoplasms**	234.8 (204.2-271.2)	97.2 (88.6-105.4)	254 (225.5-265.3)
**Other pharynx cancer**	20.5 (17.7-23.4)	16.5 (15.9-17.1)	48.5 (47-50.1)
**Ovarian cancer**	46.9 (35.8-73.6)	122.9 (109.9-128.3)	224.9 (204-233.5)

**Pancreatic cancer**	209.1 (183.3-236.3)	261.5 (246.6-269.7)	452 (430.6-463.2)
**Prostate cancer**	196.3 (152.8-238.5)	358.2 (253.8-401.3)	621.4 (475.3-743.6)
**Stomach cancer**	2410.9 (2111.8-2722.1)	170.3 (160.4-175.4)	738.3 (702.7-757.6)
**Testicular cancer**	2.2 (1.8-2.6)	1 (1-1.1)	3.3 (3.1-3.4)
**Thyroid cancer**	22.9 (19.8-30.1)	11 (10.1-11.4)	35.2 (32.6-36.5)
**Tracheal, bronchus, and lung cancer**	2028.8 (1764.5-2324.3)	1400 (1348.2-1429.9)	1808.5 (1757.8-1838.7)
**Uterine cancer**	70.9 (56-87.3)	52.6 (49.1-54.5)	85.5 (80.1-88.8)
**Cause**	**Deaths*100 in 2019 (95% UI)**		
	**China**	**United States**	**Western Europe**
**All malignant neoplasms**	22435.1 (18497.8-26498.8)	6967.4 (6336.1-7569)	11762.7 (10400.9-12815.6)
**Bladder cancer**	369.8 (313.9-433.7)	220.8 (200.2-236)	493.5 (440.3-532.4)
**Brain and central nervous system cancer**	416.4 (306.6-504.7)	159.9 (116.7-174)	239.4 (144.3-272.8)
**Breast cancer**	678 (549.5-824.5)	467.3 (428.5-494.7)	857 (757.9-914)
**Cervical cancer**	350.1 (205.4-446.1)	58.3 (48.4-62.3)	94.3 (80.8-102.7)
**Colon and rectum cancer**	2174.3 (1868.1-2512.2)	761.2 (701.4-795.4)	1636.4 (1468-1729.6)
**Esophageal cancer**	2296.9 (1770.8-2749.5)	197.5 (186.8-206.1)	320 (296.7-337)
**Gallbladder and biliary tract cancer**	302.8 (220.7-361.3)	49.9 (44.7-60.2)	163 (135.1-182)
**Hodgkin lymphoma**	18.9 (13.6-22.9)	10.9 (9.5-13.4)	18.3 (15.3-21.8)
**Kidney cancer**	186.5 (155-219.8)	180.4 (167.1-190.2)	320.3 (291.5-339.5)
**Larynx cancer**	175.2 (145.8-209)	45.2 (42.8-47.2)	87.6 (81.5-92.2)
**Leukemia**	340 (281.6-400.8)	279.4 (255.5-294.8)	409.7 (359.5-436.1)
**Lip and oral cavity cancer**	183.3 (154.2-218.8)	70.2 (65.8-73.2)	125.9 (115.6-132.2)
**Liver cancer**	1327.2 (1123.9-1565.1)	214.5 (190.5-235.1)	374.7 (345.3-399.4)
**Malignant skin melanoma**	36.1 (23.3-45.2)	100.1 (67.6-119.8)	132.6 (77.7-148.8)
**Mesothelioma**	19.1 (15.8-22.8)	30.8 (28.4-33)	98.4 (90.1-104)
**Multiple myeloma**	110.7 (84.4-135.3)	170.2 (152.6-194)	249.5 (202.7-268.4)

**Nasopharynx cancer**	191.1 (159.4-225.9)	8.8 (8.3-9.2)	15.6 (14.5-16.6)
**Non-Hodgkin lymphoma**	325.2 (276.2-379.5)	276.4 (254.2-291.9)	353.2 (316.2-376.8)
**Non-melanoma skin cancer**	136.9 (115.7-157.1)	42 (36.5-44.7)	63.2 (53.3-68)
**Other malignant neoplasms**	542.9 (434-639.8)	170.6 (149.5-183.5)	350.9 (293.3-380.8)
**Other pharynx cancer**	44.3 (35.9-53.1)	27 (25.6-28.4)	78.3 (72.4-84)
**Ovarian cancer**	212.5 (147.4-268.1)	174.8 (156.9-189.5)	276.2 (240.1-301.1)
**Pancreatic cancer**	990.2 (846.4-1146.4)	536.9 (499.8-563.7)	865.6 (780.7-923.6)
**Prostate cancer**	532.2 (420.5-696.7)	477.8 (408.1-697.8)	951.3 (787.2-1320.8)
**Stomach cancer**	3628 (3052.9-4225.4)	172.4 (158.9-180.6)	589.7 (529.4-626)
**Testicular cancer**	6.6 (5.2-8)	1.9 (1.7-2)	4 (3.6-4.4)
**Thyroid cancer**	59.1 (48.7-69)	22.5 (20.2-23.6)	39.8 (34.6-42.8)
**Tracheal, bronchus, and lung cancer**	6688.9 (5651.6-7824.3)	1944 (1821.1-2023.8)	2422.3 (2257.5-2517.3)
**Uterine cancer**	91.6 (71.3-133.6)	95.9 (88.9-101)	131.7 (115.8-140.4)
**Cause**	**ASDR in 1990 (95% UI)**		
	**China**	**United States**	**Western Europe**
**All malignant neoplasms**	790 (648-934.1)	826 (754.3-870.7)	836.7 (768.5-891.4)
**Bladder cancer**	13.9 (11.9-15.8)	20.1 (18.6-21.1)	34.2 (32.1-35.8)
**Brain and central nervous system cancer**	11.6 (9.2-15.7)	15.7 (13.1-19)	14.5 (12.4-18.7)
**Breast cancer**	17.9 (14.9-21.2)	68 (63.3-70.9)	71.2 (66.3-74.5)
**Cervical cancer**	12.2 (9.7-19.3)	7.3 (6.3-7.8)	9.8 (8.7-10.7)
**Colon and rectum cancer**	48.6 (42.5-55.2)	105.3 (98.3-109.7)	118.1 (110.6-123)
**Esophageal cancer**	114.3 (73.3-134.4)	18.1 (17.2-18.9)	22.3 (21.2-23.2)
**Gallbladder and biliary tract cancer**	8.3 (6.7-13.4)	7.2 (5.9-7.9)	16 (12.4-17.4)
**Hodgkin lymphoma**	1.8 (1-2.4)	1.7 (1.3-1.9)	2 (1.6-2.3)
**Kidney cancer**	3 (2.5-3.4)	17.7 (16.6-18.5)	17.4 (16.3-18.5)
**Larynx cancer**	6.9 (5.8-7.9)	6.7 (6.3-7)	11 (10.5-11.6)

**Leukemia**	12.8 (10.6-15.7)	29.8 (27.8-31.2)	24.4 (22.9-25.5)
**Lip and oral cavity cancer**	4.2 (3.6-4.8)	9.2 (8.7-9.6)	9.9 (9.3-10.4)
**Liver cancer**	103.1 (86.7-124)	9.9 (9.3-10.3)	17.7 (16.7-18.6)
**Malignant skin melanoma**	1.3 (0.9-1.9)	8.9 (6.5-11.6)	6.5 (5.2-9.3)
**Mesothelioma**	0.6 (0.4-0.8)	3.6 (3.3-4)	5.7 (5-6.4)
**Multiple myeloma**	3.1 (2.5-4.4)	16.7 (13.7-17.6)	12.3 (11-14)
**Nasopharynx cancer**	10.7 (9-12.7)	1.3 (1.3-1.4)	1.8 (1.6-1.9)
**Non-Hodgkin lymphoma**	6.9 (6-7.9)	32 (30.1-33.2)	20.9 (19.8-21.8)
**Non-melanoma skin cancer**	3.5 (3-4.1)	3.6 (3.3-3.8)	3.4 (3-3.6)
**Other malignant neoplasms**	18.6 (15.9-22)	17.3 (15.3-19.1)	25.1 (21.4-26.7)
**Other pharynx cancer**	1.6 (1.3-1.8)	3.1 (2.9-3.3)	5 (4.7-5.4)
**Ovarian cancer**	3.5 (2.7-5.6)	22 (19.5-23.3)	22.3 (19.8-23.6)
**Pancreatic cancer**	16.2 (14-18.5)	46.2 (43.3-48.1)	44.1 (41.5-45.9)
**Prostate cancer**	18.2 (13.7-23.4)	61 (41.7-69.1)	58.7 (43.6-71.9)
**Stomach cancer**	186.2 (161.9-211.5)	29.8 (27.9-31)	71.6 (67.3-74.4)
**Testicular cancer**	0.2 (0.1-0.2)	0.2 (0.2-0.2)	0.3 (0.3-0.4)
**Thyroid cancer**	1.9 (1.6-2.6)	1.9 (1.8-2.1)	3.4 (3.1-3.6)
**Tracheal, bronchus, and lung cancer**	153.5 (132.2-176.7)	252.6 (242.3-259.3)	178.7 (172.5-183.5)
**Uterine cancer**	5.3 (4.1-6.7)	9.2 (8.4-9.8)	8.4 (7.7-9)

**Cause**	**ASDR in 2019 (95% UI)**		
	**China**	**United States**	**Western Europe**
**All malignant neoplasms**	688.8 (561.2-817.5)	692.4 (621.9-766.4)	696.2 (611.2-775.4)
**Bladder cancer**	12.3 (10.4-14.5)	21.3 (19.1-23.2)	27 (23.9-29.8)
**Brain and central nervous system cancer**	12.4 (8.8-15.2)	16.3 (11.8-18.4)	15.8 (9.5-18.4)
**Breast cancer**	20.5 (16.5-25)	46.2 (41.6-49.8)	50.6 (44.6-55.3)
**Cervical cancer**	10.4 (6.2-13.2)	5.9 (4.8-6.5)	5.7 (4.9-6.5)

**Colon and rectum cancer**	68.8 (58.5-79.5)	74.7 (68.2-79.3)	92.8 (83.3-99.7)
**Esophageal cancer**	70.3 (52.5-84.2)	19.9 (18.4-21.3)	20 (18.1-21.7)
**Gallbladder and biliary tract cancer**	9.6 (6.9-11.5)	4.9 (4.3-6.1)	9.3 (7.7-10.9)
**Hodgkin lymphoma**	0.6 (0.4-0.7)	1.1 (0.9-1.4)	1.1 (0.9-1.4)
**Kidney cancer**	5.8 (4.8-6.9)	18 (16.2-19.6)	19 (16.8-21)
**Larynx cancer**	5.3 (4.3-6.3)	4.6 (4.2-4.9)	5.6 (5.1-6.1)
**Leukemia**	10.2 (8.3-12.3)	27.4 (24.8-29.5)	23.3 (20.3-25.4)
**Lip and oral cavity cancer**	5.6 (4.7-6.7)	7 (6.4-7.5)	7.9 (7.2-8.6)
**Liver cancer**	39.4 (33.1-46.4)	21.8 (18.9-24.4)	23 (20.6-25.1)
**Malignant skin melanoma**	1.2 (0.7-1.5)	10 (6.8-12.5)	8 (4.7-9.4)
**Mesothelioma**	0.6 (0.5-0.7)	3.1 (2.8-3.4)	6 (5.4-6.5)
**Multiple myeloma**	3.3 (2.4-4.1)	16.9 (14.8-20.2)	14.5 (11.6-16.3)
**Nasopharynx cancer**	5.6 (4.6-6.7)	0.9 (0.8-1)	1 (0.9-1.2)
**Non-Hodgkin lymphoma**	9.7 (8.2-11.5)	27.2 (24.8-29.1)	20.4 (18.3-22.1)
**Non-melanoma skin cancer**	4.6 (3.8-5.4)	4 (3.5-4.4)	3.3 (2.8-3.6)
**Other malignant neoplasms**	16.8 (13.3-19.9)	16.9 (14.5-18.7)	20.5 (17-22.8)
**Other pharynx cancer**	1.3 (1.1-1.6)	2.8 (2.5-3)	5.5 (4.7-6.3)
**Ovarian cancer**	6.2 (4.3-7.9)	17.5 (15.5-19.6)	16.9 (14.6-19)
**Pancreatic cancer**	30.2 (25.5-35.1)	53.6 (49.1-57.3)	51.7 (46.3-56.4)
**Prostate cancer**	17.7 (13.6-23.3)	46 (38.8-69)	50.9 (41.8-72.7)
**Stomach cancer**	111.6 (93.2-130.8)	17 (15.5-18.1)	33.8 (30.2-36.5)
**Testicular cancer**	0.2 (0.2-0.3)	0.2 (0.2-0.2)	0.2 (0.2-0.3)
**Thyroid cancer**	1.9 (1.5-2.3)	2.2 (2-2.4)	2.3 (2-2.6)
**Tracheal, bronchus, and lung cancer**	204.1 (170.8-240)	195.2 (181.9-205.7)	152.3 (140.9-161.4)
**Uterine cancer**	2.7 (2.1-4.1)	9.6 (8.6-10.4)	7.8 (6.8-8.6)
**Cause**		**AAPC (95% CI)**	

	China	United States	Western Europe
**All malignant neoplasms**	-0.5 (-0.65 to -0.35)	-0.62 (-0.77 to -0.46)	-0.64 (-0.7 to -0.59)
**Bladder cancer**	-0.45 (-0.67 to -0.22)	0.2 (0.18 to 0.22)	-0.84 (-0.9 to -0.79)
**Brain and central nervous system cancer**	0.23 (0.04 to 0.42)	0.12 (0.01 to 0.22)	0.29 (0.22 to 0.37)
**Breast cancer**	0.38 (0.17 to 0.6)	-1.37 (-1.46 to -1.27)	-1.17 (-1.28 to -1.07)
**Cervical cancer**	-0.6 (-0.96 to -0.24)	-0.74 (-0.92 to -0.55)	-1.83 (-1.96 to -1.71)
**Colon and rectum cancer**	1.18 (0.91 to 1.44)	-1.21 (-1.37 to -1.04)	-0.85 (-0.99 to -0.71)
**Esophageal cancer**	-1.73 (-1.86 to -1.59)	0.33 (0.23 to 0.42)	-0.38 (-0.44 to -0.32)
**Gallbladder and biliary tract cancer**	0.49 (0.34 to 0.63)	-1.31 (-1.38 to -1.23)	-1.86 (-1.96 to -1.76)
**Hodgkin lymphoma**	-4.05 (-4.27 to -3.83)	-1.4 (-1.52 to -1.27)	-2.06 (-2.16 to -1.96)
**Kidney cancer**	2.3 (2.06 to 2.54)	0.08 (-0.05 to 0.22)	0.28 (0.2 to 0.36)
**Larynx cancer**	-0.92 (-1.1 to -0.74)	-1.29 (-1.43 to -1.14)	-2.36 (-2.43 to -2.29)
**Leukemia**	-0.77 (-0.93 to -0.62)	-0.29 (-0.42 to -0.15)	-0.16 (-0.27 to -0.05)
**Lip and oral cavity cancer**	0.94 (0.79 to 1.1)	-0.93 (-1.1 to -0.75)	-0.77 (-0.89 to -0.65)
**Liver cancer**	-3.24 (-3.59 to -2.9)	2.77 (2.62 to 2.92)	0.89 (0.79 to 0.99)
**Malignant skin melanoma**	-0.36 (-0.46 to -0.25)	0.42 (0.25 to 0.58)	0.72 (0.6 to 0.84)
**Mesothelioma**	0.03 (-0.17 to 0.22)	-0.55 (-0.66 to -0.43)	0.17 (0.07 to 0.27)
**Multiple myeloma**	0.13 (0.01 to 0.25)	0.05 (-0.02 to 0.12)	0.57 (0.5 to 0.65)
**Nasopharynx cancer**	-2.27 (-2.48 to -2.07)	-1.34 (-1.48 to -1.2)	-1.82 (-1.94 to -1.7)
**Non-Hodgkin lymphoma**	1.15 (0.88 to 1.42)	-0.56 (-0.74 to -0.38)	-0.09 (-0.18 to 0)
**Non-melanoma skin cancer**	0.87 (0.69 to 1.05)	0.34 (0.18 to 0.5)	-0.11 (-0.2 to -0.02)
**Other malignant neoplasms**	-0.38 (-0.52 to -0.24)	-0.07 (-0.13 to -0.01)	-0.69 (-0.81 to -0.58)
**Other pharynx cancer**	-0.61 (-0.78 to -0.45)	-0.36 (-0.55 to -0.17)	0.28 (0.14 to 0.42)
**Ovarian cancer**	1.92 (1.73 to 2.12)	-0.77 (-0.95 to -0.59)	-0.96 (-1.11 to -0.8)
**Pancreatic cancer**	2.14 (1.86 to 2.43)	0.53 (0.31 to 0.75)	0.53 (0.4 to 0.66)
**Prostate cancer**	-0.12 (-0.26 to 0.02)	-0.96 (-1.05 to -0.88)	-0.5 (-0.55 to -0.44)

**Stomach cancer**	-1.79 (-1.96 to -1.62)	-1.91 (-2.1 to -1.72)	-2.57 (-2.72 to -2.43)
**Testicular cancer**	0.58 (0.21 to 0.95)	0.03 (-0.08 to 0.13)	-1.03 (-1.14 to -0.92)
**Thyroid cancer**	-0.1 (-0.31 to 0.11)	0.48 (0.31 to 0.65)	-1.33 (-1.5 to -1.15)
**Tracheal, bronchus, and lung cancer**	0.94 (0.69 to 1.19)	-0.87 (-1.05 to -0.69)	-0.54 (-0.68 to -0.41)
**Uterine cancer**	-2.26 (-2.62 to -1.89)	0.11 (-0.02 to 0.23)	-0.24 (-0.3 to -0.18)

Note: Estimates are for populations aged 55 years and above, both sexes combined. All malignant cancers are the sum of the following 29 cancers. Rates are reported per 100000 person-years. Cancer types are listed alphabetically. Other malignant neoplasms are cancers without a detailed GBD cause separately listed. UI=uncertainty interval. ASDR=age-standardized death rate. AAPC=average annual percentage change. CI = confidence interval. UI = uncertainty intervals.

Table S4 The DALYs and DALY rate of 29 cancers among men and women over 55+ years old in China in 1990 and 2019, and percentage change in DALYs, annual rate of change in DALY rate from 1990 to 2019.

Cause	DALYs*1000 in 1990 (95% UI)		DALYs*1000 in 2019 (95% UI)		Percentage change in DALYs (95% UI)	
	Men	Women	Men	Women	Men	Women
**Bladder cancer**	202.2 (169.2-235.5)	86.9 (71.9-104)	534.3 (435.2-648.3)	136.7 (110.3-166.9)	164.2 (103.6 to 241.7)	57.3 (17.3 to 110)
**Brain and central nervous system cancer**	210.8 (147.5-307.5)	169.5 (126.5-227.1)	504.5 (308.6-674.7)	404.8 (287.5-526.4)	139.3 (45.6 to 255.9)	138.8 (39.4 to 250.4)
**Breast cancer**	6.6 (5.2-8)	586.4 (494.9-688.1)	54.3 (41.8-67.9)	1575 (1294.3-1904.2)	727.9 (490.4 to 1035.6)	168.6 (108.5 to 250)
**Cervical cancer**	0 (0-0)	384.6 (303.3-616.5)	0 (0-0)	804.4 (461.3-1026.7)	0 (0 to 0)	109.2 (-9.7 to 201.8)
**Colon and rectum cancer**	678.7 (562.8-799.5)	593.1 (500.1-695.5)	2775.6 (2249.7-3401.8)	1610.9 (1325.3-1942.6)	309 (211.4 to 431.7)	171.6 (112.3 to 250.3)
**Esophageal cancer**	2214.1 (1386.6-2699.4)	1139.8 (611.6-1358.2)	3615.2 (2757.4-4512.1)	1018.3 (708.7-1279.9)	63.3 (22.4 to 134.6)	-10.7 (-31.7 to 30)
**Gallbladder and biliary tract cancer**	91.6 (71.9-161.9)	115.4 (88.6-203.5)	301.1 (215.2-382.2)	285.7 (176.8-364.5)	228.8 (57.8 to 386.3)	147.6 (2.1 to 268.3)
**Hodgkin lymphoma**	35.8 (17.3-48)	22.1 (10-30.3)	27.7 (18.7-36.9)	14.5 (9.4-18.7)	-22.5 (-47.9 to 49.2)	-34.4 (-53.4 to 16)
**Kidney cancer**	47.7	36.1	269.9	116.7	465.8	223

	(38.7-59.1)	(30.3-42.2)	(210.2-336.3)	(94.4-141.9)	(298.8 to 698.5)	(151.2 to 318.5)
**Larynx cancer**	163.4<br>(133.1-192.5)	46.1<br>(38.9-53.6)	319.7<br>(257.7-392.3)	60.9<br>(49.5-73.5)	95.7<br>(47.6 to 164.2)	32<br>(3.6 to 69.2)
**Leukemia**	215.9<br>(170-278.4)	189.9<br>(154.7-233.4)	432<br>(318.3-548.6)	327.4<br>(248.5-407.5)	100<br>(41.9 to 179.2)	72.4<br>(17.1 to 133.4)
**Lip and oral cavity cancer**	73.2<br>(59.4-87.2)	42.7<br>(35.5-50.7)	305.3<br>(243.5-382.8)	81.9<br>(66.5-99)	317.4<br>(202.8 to 472.7)	92<br>(46.8 to 150.6)
**Liver cancer**	2310.6<br>(1842.6-2844.9)	1085.6<br>(878.5-1342)	2096.7<br>(1644.7-2604.8)	818.4<br>(653.5-1000.2)	-9.3<br>(-34 to 26.3)	-24.6<br>(-46.2 to 3.7)
**Malignant skin melanoma**	17<br>(10.6-24.6)	17.6<br>(11.3-29.2)	35.2<br>(20.7-49.7)	40.2<br>(21.1-54.7)	106.6<br>(36.2 to 192.4)	129<br>(7.6 to 253)
**Mesothelioma**	6.6<br>(5.1-9.9)	9.6<br>(6.5-15.4)	22.8<br>(17.6-29.5)	18.3<br>(11.8-23.7)	244.2<br>(109.7 to 387.9)	91.7<br>(-1.1 to 219)
**Multiple myeloma**	46.8<br>(35-70.7)	51.1<br>(38.1-74.2)	138.7<br>(90.3-184.4)	103.9<br>(75.1-135.1)	196.2<br>(55.7 to 330.5)	103.4<br>(9.5 to 179.5)
**Nasopharynx cancer**	235.8<br>(191.4-283.9)	124.5<br>(99.8-150.3)	337.5<br>(266.8-417.6)	118.5<br>(94.1-144.9)	43.2<br>(5.9 to 95)	-4.8<br>(-28.8 to 29.3)
**Non-Hodgkin lymphoma**	118.3<br>(95.9-143.2)	84.5<br>(70.7-98.7)	480.6<br>(382.4-595.8)	247.8<br>(201.6-301.2)	306.3<br>(199.4 to 455.2)	193.3<br>(129.5 to 275.6)
**Non-melanoma skin cancer**	44.3<br>(36.7-51.8)	35.2<br>(29.3-41.9)	131<br>(106.3-159.6)	113.7<br>(86.8-140.7)	195.6<br>(125.5 to 289.4)	223.2<br>(129.2 to 329.7)
**Other malignant neoplasms**	284.5<br>(224.7-346.3)	242.8<br>(198.3-293.4)	682.5<br>(506.1-855.1)	458.2<br>(360.5-566.4)	139.9<br>(67 to 232.7)	88.7<br>(36.7 to 151.6)
**Other pharynx cancer**	34.7<br>(28.7-40.8)	12.2<br>(10-14.4)	80<br>(62.2-99.6)	16.8<br>(13.5-20.4)	130.8<br>(67 to 212.7)	37.4<br>(2.5 to 82.6)

**Ovarian cancer**	0 (0-0)	113 (85.9-176.7)	0 (0-0)	490.1 (338.3-620)	0 (0 to 0)	333.8 (121.9 to 546.1)
**Pancreatic cancer**	276.2 (224-335.7)	198.4 (165.8-231.5)	1208.5 (954-1498.9)	819.9 (665.1-998.7)	337.6 (215.7 to 514.9)	313.3 (216.5 to 437.9)
**Prostate cancer**	369.4 (282.8-445.2)	0 (0-0)	948.9 (753.9-1248)	0 (0-0)	156.9 (96.6 to 247.4)	0 (0 to 0)
**Stomach cancer**	3579.7 (2931.5-4239.2)	1824.6 (1528.7-2128.4)	5293.6 (4203.2-6488.4)	2010.4 (1635.5-2468.6)	47.9 (11.2 to 96.7)	10.2 (-14.3 to 44.2)
**Testicular cancer**	4.7 (3.9-5.7)	0 (0-0)	14.2 (11.4-17.2)	0 (0-0)	200.7 (124.6 to 301.5)	0 (0 to 0)
**Thyroid cancer**	18.4 (14.9-23.2)	31.5 (25.8-42.9)	64.8 (49.7-80.1)	53.1 (43-65.1)	251.5 (140.7 to 380.1)	68.3 (23.9 to 123)
**Tracheal, bronchus, and lung cancer**	3268.7 (2671.2-3927.8)	1351 (1122-1585.3)	9479.7 (7467.5-11725.2)	3964.8 (3220.9-4834.6)	190 (109.4 to 292.8)	193.5 (126.4 to 282.5)
**Uterine cancer**	0 (0-0)	169.8 (132-209.5)	0 (0-0)	217.2 (172.2-314)	0 (0 to 0)	27.9 (-3.5 to 82.6)

**Cause**	**DALY rate in 1990 (95% UI)**		**DALY rate in 2019 (95% UI)**		**Annual rate of change in DALY rate (95% UI)**	
	**Men**	**Women**	**Men**	**Women**	**Men**	**Women**
**Bladder cancer**	168.1 (140.6-195.7)	63.8 (52.9-76.4)	169.3 (137.8-205.4)	38.8 (31.3-47.3)	0.01 (-0.22 to 0.3)	-0.39 (-0.55 to -0.19)
**Brain and central nervous system cancer**	175.2 (122.6-255.5)	124.6 (93-166.9)	159.8 (97.8-213.7)	114.8 (81.5-149.3)	-0.09 (-0.45 to 0.36)	-0.08 (-0.46 to 0.35)
**Breast cancer**	5.5	430.9	17.2	446.7	2.16	0.04

	(4.4-6.7)	(363.7-505.7)	(13.3-21.5)	(367-540)	(1.25 to 3.33)	(-0.2 to 0.35)
**Cervical cancer**	0<br>(0-0)	282.6<br>(222.9-453.1)	0<br>(0-0)	228.1<br>(130.8-291.2)	0<br>(0 to 0)	-0.19<br>(-0.65 to 0.16)
**Colon and rectum cancer**	564.1<br>(467.8-664.5)	435.9<br>(367.6-511.2)	879.2<br>(712.6-1077.6)	456.8<br>(375.8-550.9)	0.56<br>(0.19 to 1.03)	0.05<br>(-0.18 to 0.35)
**Esophageal cancer**	1840.2<br>(1152.4-2243.6)	837.7<br>(449.5-998.2)	1145.2<br>(873.4-1429.3)	288.8<br>(201-363)	-0.38<br>(-0.53 to -0.11)	-0.66<br>(-0.74 to -0.5)
**Gallbladder and biliary tract cancer**	76.1<br>(59.7-134.6)	84.8<br>(65.1-149.6)	95.4<br>(68.2-121.1)	81<br>(50.1-103.4)	0.25<br>(-0.4 to 0.85)	-0.04<br>(-0.61 to 0.42)
**Hodgkin lymphoma**	29.7<br>(14.4-39.9)	16.2<br>(7.3-22.2)	8.8<br>(5.9-11.7)	4.1<br>(2.7-5.3)	-0.7<br>(-0.8 to -0.43)	-0.75<br>(-0.82 to -0.55)
**Kidney cancer**	39.7<br>(32.1-49.1)	26.5<br>(22.3-31)	85.5<br>(66.6-106.5)	33.1<br>(26.8-40.2)	1.16<br>(0.52 to 2.04)	0.25<br>(-0.03 to 0.61)
**Larynx cancer**	135.8<br>(110.6-160)	33.9<br>(28.6-39.4)	101.3<br>(81.6-124.3)	17.3<br>(14-20.8)	-0.25<br>(-0.44 to 0.01)	-0.49<br>(-0.6 to -0.35)
**Leukemia**	179.5<br>(141.3-231.4)	139.6<br>(113.7-171.5)	136.8<br>(100.8-173.8)	92.8<br>(70.5-115.6)	-0.24<br>(-0.46 to 0.06)	-0.33<br>(-0.55 to -0.1)
**Lip and oral cavity cancer**	60.8<br>(49.4-72.5)	31.4<br>(26.1-37.3)	96.7<br>(77.1-121.3)	23.2<br>(18.9-28.1)	0.59<br>(0.15 to 1.18)	-0.26<br>(-0.43 to -0.03)
**Liver cancer**	1920.4<br>(1531.5-2364.5)	797.9<br>(645.6-986.2)	664.2<br>(521-825.1)	232.1<br>(185.3-283.6)	-0.65<br>(-0.75 to -0.52)	-0.71<br>(-0.79 to -0.6)
**Malignant skin melanoma**	14.2<br>(8.8-20.5)	12.9<br>(8.3-21.5)	11.1<br>(6.5-15.7)	11.4<br>(6-15.5)	-0.21<br>(-0.48 to 0.11)	-0.12<br>(-0.58 to 0.36)
**Mesothelioma**	5.5	7	7.2	5.2	0.31	-0.26

	(4.2-8.2)	(4.8-11.3)	(5.6-9.3)	(3.3-6.7)	(-0.2 to 0.86)	(-0.62 to 0.23)
**Multiple myeloma**	38.9<br>(29.1-58.7)	37.5<br>(28-54.5)	43.9<br>(28.6-58.4)	29.5<br>(21.3-38.3)	0.13<br>(-0.41 to 0.64)	-0.22<br>(-0.58 to 0.08)
**Nasopharynx cancer**	195.9<br>(159.1-236)	91.5<br>(73.4-110.4)	106.9<br>(84.5-132.3)	33.6<br>(26.7-41.1)	-0.45<br>(-0.6 to -0.26)	-0.63<br>(-0.73 to -0.5)
**Non-Hodgkin lymphoma**	98.3<br>(79.7-119)	62.1<br>(52-72.5)	152.2<br>(121.1-188.7)	70.3<br>(57.2-85.4)	0.55<br>(0.14 to 1.12)	0.13<br>(-0.11 to 0.45)
**Non-melanoma skin cancer**	36.8<br>(30.5-43.1)	25.9<br>(21.5-30.8)	41.5<br>(33.7-50.5)	32.3<br>(24.6-39.9)	0.13<br>(-0.14 to 0.48)	0.25<br>(-0.12 to 0.66)
**Other malignant neoplasms**	236.5<br>(186.8-287.8)	178.5<br>(145.8-215.6)	216.2<br>(160.3-270.9)	129.9<br>(102.2-160.6)	-0.09<br>(-0.36 to 0.27)	-0.27<br>(-0.47 to -0.03)
**Other pharynx cancer**	28.8<br>(23.9-33.9)	9<br>(7.3-10.6)	25.3<br>(19.7-31.6)	4.8<br>(3.8-5.8)	-0.12<br>(-0.36 to 0.19)	-0.47<br>(-0.6 to -0.3)
**Ovarian cancer**	0<br>(0-0)	83<br>(63.1-129.9)	0<br>(0-0)	139<br>(95.9-175.8)	0<br>(0 to 0)	0.67<br>(-0.14 to 1.49)
**Pancreatic cancer**	229.5<br>(186.2-279)	145.8<br>(121.9-170.2)	382.8<br>(302.2-474.8)	232.5<br>(188.6-283.2)	0.67<br>(0.2 to 1.34)	0.59<br>(0.22 to 1.08)
**Prostate cancer**	307<br>(235-370.1)	0<br>(0-0)	300.6<br>(238.8-395.3)	0<br>(0-0)	-0.02<br>(-0.25 to 0.32)	0<br>(0 to 0)
**Stomach cancer**	2975.2<br>(2436.5-3523.3)	1341<br>(1123.5-1564.2)	1676.8<br>(1331.4-2055.3)	570.1<br>(463.8-700.1)	-0.44<br>(-0.58 to -0.25)	-0.57<br>(-0.67 to -0.44)
**Testicular cancer**	3.9<br>(3.2-4.7)	0<br>(0-0)	4.5<br>(3.6-5.5)	0<br>(0-0)	0.15<br>(-0.14 to 0.53)	0<br>(0 to 0)
**Thyroid cancer**	15.3<br>(12.4-19.3)	23.2<br>(18.9-31.5)	20.5<br>(15.7-25.4)	15.1<br>(12.2-18.5)	0.34<br>(-0.08 to 0.83)	-0.35<br>(-0.52 to -0.14)

**Tracheal, bronchus, and lung cancer**	2716.8 (2220.1-3264.5)	992.9 (824.6-1165.1)	3002.8 (2365.4-3714.1)	1124.4 (913.4-1371)	0.11 (-0.2 to 0.5)	0.13 (-0.13 to 0.48)
**Uterine cancer**	0 (0-0)	124.8 (97-154)	0 (0-0)	61.6 (48.8-89)	0 (0 to 0)	-0.51 (-0.63 to -0.3)

Note: Estimates are for populations aged 55 years and above among men and women in China. Rates are reported per 100000 person-years. Cancer types are listed alphabetically. Other malignant neoplasms are cancers without a detailed GBD cause separately listed. DALYs=disability-adjusted life-years. UI=uncertainty interval.

Table S5 The death cases and death rate of 29 cancers among men and women over 55+ years old in China in 1990 and 2019, and percentage change in death cases, annual rate of change in death rate from 1990 to 2019

Cause	Deaths*100 in 1990 (95% UI)		Deaths*100 in 2019 (95% UI)		Percentage change in deaths (95% UI)	
	Men	Women	Men	Women	Men	Women
**Bladder cancer**	102.5 (86.2-118.1)	45.7 (38.1-54.5)	290 (236.5-351.7)	79.7 (64.4-97.6)	182.9 (119.1 to 264.1)	74.6 (31 to 131.5)
**Brain and central nervous system cancer**	86.1 (61.1-125.7)	72.5 (55.7-98.6)	226.9 (136.8-297.2)	189.5 (132.2-244.5)	163.4 (58.9 to 280.9)	161.6 (49.3 to 272.5)
**Breast cancer**	2.7 (2.2-3.3)	233.6 (196.2-274.7)	22.2 (17.1-27.6)	655.8 (527.7-803.4)	717.4 (485.6 to 1025.8)	180.7 (118.6 to 264.7)
**Cervical cancer**	0 (0-0)	163 (129.5-259.2)	0 (0-0)	350.1 (205.4-446.1)	0 (0 to 0)	114.8 (-2.7 to 207.6)
**Colon and rectum cancer**	303 (252.5-353.7)	279.9 (236.3-327.9)	1340.4 (1084.5-1632.4)	834 (677.7-1004.9)	342.3 (239 to 472.1)	197.9 (134.9 to 280.9)
**Esophageal cancer**	953 (598-1149.3)	541.3 (302.2-640.2)	1729.8 (1305.8-2140.7)	567.1 (380.3-715.1)	81.5 (37.5 to 155.9)	4.8 (-19 to 44.4)
**Gallbladder and biliary tract cancer**	42.2 (33.5-73.3)	55.9 (43.4-98.8)	152 (110.9-191.2)	150.8 (92.5-192.1)	259.9 (76.8 to 424.1)	169.8 (13.1 to 296.2)
**Hodgkin lymphoma**	14.9 (7.2-19.9)	10 (4.6-13.5)	12.1 (7.9-16)	6.8 (4.3-8.8)	-18.7 (-45.1 to 56.6)	-32.2 (-51.7 to 18.1)
**Kidney cancer**	20.6 (16.7-25.2)	16.5 (14-19.1)	127.9 (99.8-158.3)	58.6 (47.1-71.2)	521.4 (346.9 to 771.1)	254.1 (176.2 to 358.6)

**Larynx cancer**	69.1 (56.8-80.9)	21.3 (18-24.6)	144.4 (115.6-177)	30.8 (24.8-37)	109 (58.1 to 178.9)	44.8 (13.9 to 84.7)
**Leukemia**	90.2 (71.7-116.4)	80.2 (65.5-100.3)	193.9 (142.6-246.7)	146.1 (111.4-182.3)	114.9 (50.6 to 194.5)	82.2 (22.1 to 146.5)
**Lip and oral cavity cancer**	31.6 (25.8-37.4)	19.9 (16.7-23.5)	141.6 (114.3-175.1)	41.7 (33.8-50.3)	347.9 (227.5 to 513.9)	109.7 (61.6 to 171.7)
**Liver cancer**	931.5 (749.8-1140.5)	485.7 (398.8-597.7)	922.9 (733.9-1141.3)	404.3 (325.6-493)	-0.9 (-27.5 to 35.9)	-16.8 (-40.1 to 12.8)
**Malignant skin melanoma**	7.2 (4.5-10.4)	8.3 (5.4-13.8)	15.6 (9.5-22.3)	20.5 (11.1-27.6)	118.2 (49.6 to 203.7)	147.2 (18.2 to 272.8)
**Mesothelioma**	2.8 (2.2-4.2)	4.3 (3-6.9)	10.2 (7.9-13.1)	8.9 (5.7-11.5)	260.8 (123.8 to 409.1)	106.9 (8.2 to 239.4)
**Multiple myeloma**	19.6 (14.9-30)	22.6 (17-32.7)	62.6 (40.6-83.5)	48.1 (34.6-62.6)	219.6 (66.9 to 360.8)	112.7 (16.7 to 188.8)
**Nasopharynx cancer**	93.7 (76.7-111.8)	53.2 (42.7-64)	139 (109-171.5)	52.2 (41.2-64.4)	48.4 (11.5 to 101.4)	-1.9 (-26.6 to 33.2)
**Non-Hodgkin lymphoma**	49.9 (40.8-59.8)	38.6 (32.6-45.1)	209.5 (167.1-260)	115.8 (93.8-140.7)	319.5 (210.4 to 469.5)	199.8 (134.9 to 284.1)
**Non-melanoma skin cancer**	21.7 (18-25.2)	17.6 (14.8-20.6)	71.2 (58.2-85.9)	65.7 (51-80.1)	228.1 (153.2 to 328.4)	273.8 (167 to 387.5)
**Other malignant neoplasms**	123.6 (98.2-149.7)	111.2 (91.7-134.8)	317.7 (231.2-398.3)	225.2 (177.8-279.5)	157.1 (76.6 to 253.1)	102.6 (47 to 168.6)
**Other pharynx cancer**	15 (12.5-17.6)	5.5 (4.6-6.5)	35.9 (28.2-44.6)	8.4 (6.7-10.2)	140.4 (75.6 to 223.1)	51.7 (15.1 to 100.6)
**Ovarian cancer**	0	46.9	0	212.5	0	352.6

	(0-0)	(35.8-73.6)	(0-0)	(147.4-268.1)	(0 to 0)	(135.4 to 572.4)
**Pancreatic cancer**	118.3 (97.3-142.7)	90.8 (76.7-106)	572.6 (455.8-703.6)	417.6 (338.1-506)	384 (254.1 to 567.7)	359.7 (255.6 to 492.9)
**Prostate cancer**	196.3 (152.8-238.5)	0 (0-0)	532.2 (420.5-696.7)	0 (0-0)	171.1 (107.7 to 254.2)	0 (0 to 0)
**Stomach cancer**	1549.2 (1275.3-1825.5)	861.6 (724.9-999.7)	2552.8 (2045.9-3106.2)	1075.2 (879.7-1312.1)	64.8 (25.2 to 116.8)	24.8 (-1.7 to 60.9)
**Testicular cancer**	2.2 (1.8-2.6)	0 (0-0)	6.6 (5.2-8)	0 (0-0)	205.4 (128.3 to 304.6)	0 (0 to 0)
**Thyroid cancer**	8.3 (6.8-10.5)	14.6 (12-20)	33.5 (25.4-41.4)	25.7 (20.4-31.7)	302.5 (167.2 to 446.2)	75.5 (28.3 to 131.4)
**Tracheal, bronchus, and lung cancer**	1412.2 (1164.5-1688.9)	616.7 (516.8-723.5)	4632.9 (3679.4-5703.5)	2056 (1667-2466.8)	228.1 (139.3 to 341.7)	233.4 (160.7 to 326.2)
**Uterine cancer**	0 (0-0)	70.9 (56-87.3)	0 (0-0)	91.6 (71.3-133.6)	0 (0 to 0)	29.1 (-2.8 to 83.5)

**Cause**	**Death rate in 1990 (95% UI)**		**Death rate in 2019 (95% UI)**		**Annual rate of change in death rate (95% UI)**	
	**Men**	**Women**	**Men**	**Women**	**Men**	**Women**
**Bladder cancer**	14.4 (12.1-16.6)	6.2 (5.2-7.4)	17 (13.8-20.6)	4.4 (3.6-5.4)	0.18 (-0.09 to 0.51)	-0.29 (-0.47 to -0.06)
**Brain and central nervous system cancer**	12.1 (8.6-17.7)	9.9 (7.6-13.4)	13.3 (8-17.4)	10.5 (7.3-13.5)	0.09 (-0.34 to 0.58)	0.06 (-0.39 to 0.51)
**Breast cancer**	0.4 (0.3-0.5)	31.8 (26.7-37.4)	1.3 (1-1.6)	36.3 (29.2-44.4)	2.4 (1.43 to 3.68)	0.14 (-0.11 to 0.48)

**Cervical cancer**	0 (0-0)	22.2 (17.6-35.3)	0 (0-0)	19.4 (11.4-24.7)	0 (0 to 0)	-0.13 (-0.6 to 0.25)
**Colon and rectum cancer**	42.7 (35.6-49.8)	38.1 (32.1-44.6)	78.4 (63.5-95.5)	46.1 (37.5-55.6)	0.84 (0.41 to 1.38)	0.21 (-0.05 to 0.55)
**Esophageal cancer**	134.2 (84.2-161.9)	73.6 (41.1-87.1)	101.2 (76.4-125.3)	31.4 (21-39.5)	-0.25 (-0.43 to 0.06)	-0.57 (-0.67 to -0.41)
**Gallbladder and biliary tract cancer**	5.9 (4.7-10.3)	7.6 (5.9-13.4)	8.9 (6.5-11.2)	8.3 (5.1-10.6)	0.5 (-0.27 to 1.18)	0.1 (-0.54 to 0.61)
**Hodgkin lymphoma**	2.1 (1-2.8)	1.4 (0.6-1.8)	0.7 (0.5-0.9)	0.4 (0.2-0.5)	-0.66 (-0.77 to -0.35)	-0.72 (-0.8 to -0.52)
**Kidney cancer**	2.9 (2.3-3.6)	2.2 (1.9-2.6)	7.5 (5.8-9.3)	3.2 (2.6-3.9)	1.58 (0.86 to 2.62)	0.44 (0.12 to 0.86)
**Larynx cancer**	9.7 (8-11.4)	2.9 (2.5-3.3)	8.5 (6.8-10.4)	1.7 (1.4-2)	-0.13 (-0.34 to 0.16)	-0.41 (-0.54 to -0.25)
**Leukemia**	12.7 (10.1-16.4)	10.9 (8.9-13.6)	11.3 (8.3-14.4)	8.1 (6.2-10.1)	-0.11 (-0.37 to 0.22)	-0.26 (-0.5 to 0)
**Lip and oral cavity cancer**	4.5 (3.6-5.3)	2.7 (2.3-3.2)	8.3 (6.7-10.2)	2.3 (1.9-2.8)	0.86 (0.36 to 1.55)	-0.15 (-0.34 to 0.1)
**Liver cancer**	131.2 (105.6-160.6)	66.1 (54.2-81.3)	54 (42.9-66.8)	22.4 (18-27.3)	-0.59 (-0.7 to -0.44)	-0.66 (-0.76 to -0.54)
**Malignant skin melanoma**	1 (0.6-1.5)	1.1 (0.7-1.9)	0.9 (0.6-1.3)	1.1 (0.6-1.5)	-0.09 (-0.38 to 0.26)	0 (-0.52 to 0.52)
**Mesothelioma**	0.4 (0.3-0.6)	0.6 (0.4-0.9)	0.6 (0.5-0.8)	0.5 (0.3-0.6)	0.5 (-0.07 to 1.11)	-0.16 (-0.56 to 0.38)
**Multiple myeloma**	2.8 (2.1-4.2)	3.1 (2.3-4.4)	3.7 (2.4-4.9)	2.7 (1.9-3.5)	0.33 (-0.31 to 0.91)	-0.14 (-0.53 to 0.17)

**Nasopharynx cancer**	13.2<br>(10.8-15.7)	7.2<br>(5.8-8.7)	8.1<br>(6.4-10)	2.9<br>(2.3-3.6)	-0.38<br>(-0.54 to -0.16)	-0.6<br>(-0.7 to -0.46)
**Non-Hodgkin lymphoma**	7<br>(5.7-8.4)	5.3<br>(4.4-6.1)	12.3<br>(9.8-15.2)	6.4<br>(5.2-7.8)	0.74<br>(0.29 to 1.37)	0.22<br>(-0.05 to 0.56)
**Non-melanoma skin cancer**	3.1<br>(2.5-3.6)	2.4<br>(2-2.8)	4.2<br>(3.4-5)	3.6<br>(2.8-4.4)	0.36<br>(0.05 to 0.78)	0.52<br>(0.09 to 0.98)
**Other malignant neoplasms**	17.4<br>(13.8-21.1)	15.1<br>(12.5-18.3)	18.6<br>(13.5-23.3)	12.5<br>(9.8-15.5)	0.07<br>(-0.27 to 0.47)	-0.18<br>(-0.4 to 0.09)
**Other pharynx cancer**	2.1<br>(1.8-2.5)	0.8<br>(0.6-0.9)	2.1<br>(1.6-2.6)	0.5<br>(0.4-0.6)	0<br>(-0.27 to 0.34)	-0.38<br>(-0.53 to -0.18)
**Ovarian cancer**	0<br>(0-0)	6.4<br>(4.9-10)	0<br>(0-0)	11.7<br>(8.2-14.8)	0<br>(0 to 0)	0.84<br>(-0.04 to 1.73)
**Pancreatic cancer**	16.7<br>(13.7-20.1)	12.4<br>(10.4-14.4)	33.5<br>(26.7-41.2)	23.1<br>(18.7-28)	1.01<br>(0.47 to 1.77)	0.87<br>(0.45 to 1.41)
**Prostate cancer**	27.6<br>(21.5-33.6)	0<br>(0-0)	31.1<br>(24.6-40.8)	0<br>(0-0)	0.13<br>(-0.14 to 0.47)	0<br>(0 to 0)
**Stomach cancer**	218.2<br>(179.6-257.1)	117.2<br>(98.6-136)	149.4<br>(119.7-181.8)	59.5<br>(48.6-72.5)	-0.32<br>(-0.48 to -0.1)	-0.49<br>(-0.6 to -0.35)
**Testicular cancer**	0.3<br>(0.2-0.4)	0<br>(0-0)	0.4<br>(0.3-0.5)	0<br>(0-0)	0.27<br>(-0.05 to 0.68)	0<br>(0 to 0)
**Thyroid cancer**	1.2<br>(1-1.5)	2<br>(1.6-2.7)	2<br>(1.5-2.4)	1.4<br>(1.1-1.8)	0.67<br>(0.11 to 1.27)	-0.29<br>(-0.48 to -0.06)
**Tracheal, bronchus, and lung cancer**	198.9<br>(164-237.9)	83.9<br>(70.3-98.4)	271.1<br>(215.3-333.7)	113.7<br>(92.2-136.4)	0.36<br>(-0.01 to 0.83)	0.36<br>(0.06 to 0.73)
**Uterine cancer**	0<br>(0-0)	9.6<br>(7.6-11.9)	0<br>(0-0)	5.1<br>(3.9-7.4)	0<br>(0 to 0)	-0.48<br>(-0.6 to -0.25)

Note: Estimates are for populations aged 55 years and above among men and women in China. Rates are reported per 100000 person-years. Cancer types are listed alphabetically. Other malignant neoplasms are cancers without a detailed GBD cause separately listed. UI=uncertainty interval.

Table S6 The incident cases and incident rate of 29 cancers among men and women over 55+ years old in China in 1990 and 2019, and percentage change in incident cases, annual rate of change in incidence rate from 1990 to 2019

Cause	Incident cases*1000 in 1990 (95% UI)		Incident cases*1000 in 2019 (95% UI)		Percentage change in incident cases (95% UI)	
	Men	Women	Men	Women	Men	Women
**Bladder cancer**	13.9 (11.7-16.3)	5.4 (4.5-6.4)	67.1 (53.7-81.6)	14.3 (11.3-17.8)	382.2 (272.8 to 535.4)	165.6 (95.4 to 259.2)
**Brain and central nervous system cancer**	8.4 (5.9-12.5)	7.1 (5.4-9.8)	23.8 (14.9-31.5)	23 (16.7-29.2)	182.3 (65.4 to 307.2)	221.7 (80.9 to 365)
**Breast cancer**	0.4 (0.3-0.4)	37.2 (31.2-43.7)	5.2 (3.9-6.6)	203.3 (163-252.6)	1336 (921.1 to 1910.1)	447 (317.8 to 622)
**Cervical cancer**	0 (0-0)	18.3 (14.6-29.9)	0 (0-0)	48.2 (26.7-61.1)	0 (0 to 0)	163 (13.3 to 282.5)
**Colon and rectum cancer**	38.2 (32-44.9)	34.3 (28.9-39.8)	290.7 (232.6-358.8)	173.5 (141.1-212.6)	660.7 (481.9 to 904)	406.3 (295.1 to 552.3)
**Esophageal cancer**	91.7 (60-110.5)	51.8 (28.5-61.6)	177.6 (131.2-219.9)	64.9 (41.5-82.1)	93.6 (47.2 to 171.1)	25.4 (-4.3 to 75.7)
**Gallbladder and biliary tract cancer**	4.2 (3.3-7.3)	5.5 (4.3-9.9)	16.5 (11-20.6)	16.7 (10-21.8)	290.8 (82.4 to 489)	203 (21.1 to 347.7)
**Hodgkin lymphoma**	1.6 (0.7-2.1)	1 (0.5-1.4)	2.8 (1.8-3.7)	1.5 (1-2)	78.8 (21.1 to 215.3)	50.8 (5.8 to 157.8)
**Kidney cancer**	2.8 (2.2-3.5)	2.2 (1.8-2.5)	24.6 (19.2-30.9)	10.6 (8.6-13.2)	775.1 (526.9 to 1130.7)	388.2 (276 to 531.9)
**Larynx cancer**	8.2 (6.8-9.7)	2.4 (2-2.8)	31.6 (24.9-39.3)	5.5 (4.4-6.7)	283.4 (188.5 to 419)	131.3 (76.9 to 203.3)
**Leukemia**	9.8	8.7	34.2	28.4	249.2	224.9

	(7.7-12.7)	(7.2-10.7)	(25.6-43.6)	(21.3-36.3)	(145.8 to 386.9)	(117.1 to 343.5)
**Lip and oral cavity cancer**	4.4<br>(3.6-5.2)	3.1<br>(2.6-3.7)	25.1<br>(19.7-31.1)	8.5<br>(6.9-10.4)	467.1<br>(316.2 to 690.3)	172.2<br>(108.8 to 252.1)
**Liver cancer**	91.4<br>(73.3-113.3)	45.8<br>(37.7-56.5)	99.6<br>(79.7-123.3)	40.2<br>(32.3-49.1)	9<br>(-20.5 to 51.2)	-12.3<br>(-36.6 to 18.7)
**Malignant skin melanoma**	0.8<br>(0.5-1.2)	0.9<br>(0.6-1.5)	4.1<br>(2.4-6)	4.8<br>(2.5-6.5)	399.4<br>(220.5 to 614.6)	422.9<br>(142.9 to 677.2)
**Mesothelioma**	0.3<br>(0.2-0.4)	0.4<br>(0.3-0.7)	1<br>(0.8-1.3)	0.8<br>(0.5-1.1)	246<br>(108.6 to 396.7)	102<br>(4 to 227.7)
**Multiple myeloma**	2.2<br>(1.7-3.3)	2.4<br>(1.9-3.6)	8.6<br>(5.8-11.6)	6.1<br>(4.4-8)	294.7<br>(100.6 to 467.4)	152.7<br>(35.5 to 253.5)
**Nasopharynx cancer**	9.7<br>(7.9-11.5)	5.4<br>(4.4-6.4)	35<br>(27.1-44.5)	12.4<br>(9.7-15.5)	259.9<br>(165.5 to 391.9)	131.2<br>(72 to 221.6)
**Non-Hodgkin lymphoma**	4.8<br>(3.9-5.8)	3.6<br>(3.1-4.3)	39.8<br>(31.5-49.7)	21.8<br>(17.4-27)	728.6<br>(522.4 to 1022.6)	497.7<br>(351.3 to 679.2)
**Non-melanoma skin cancer**	12.6<br>(10.3-15.3)	11.2<br>(9-13.9)	124.1<br>(105.1-145.5)	88.5<br>(73.4-105.3)	886.7<br>(818.4 to 977.4)	692.7<br>(644.4 to 763.1)
**Other malignant neoplasms**	14.8<br>(11.7-18.2)	11.7<br>(9.7-14)	81<br>(60.5-101.5)	45<br>(35.9-55.8)	445.6<br>(280.3 to 658.6)	283.9<br>(180.7 to 421.1)
**Other pharynx cancer**	1.5<br>(1.3-1.8)	0.6<br>(0.5-0.7)	5.3<br>(4.2-6.7)	1.8<br>(1.4-2.2)	252.2<br>(160 to 372.5)	201.6<br>(124.7 to 301.6)
**Ovarian cancer**	0<br>(0-0)	5.2<br>(4-8.2)	0<br>(0-0)	25.9<br>(17.7-32.5)	0<br>(0 to 0)	394.9<br>(154.1 to 624.5)
**Pancreatic cancer**	11.4<br>(9.3-13.7)	8.6<br>(7.2-10)	55.3<br>(44.1-67.7)	39.3<br>(31.7-48.2)	385.4<br>(259.7 to 565.4)	359.1<br>(257.3 to 486.1)
**Prostate cancer**	24.9	0	145.5	0	483.9	0

	(19-30)	(0-0)	(112.5-193.1)	(0-0)	(335.6 to 704.4)	(0 to 0)
**Stomach cancer**	157.5<br>(131.5-184.9)	83.5<br>(70.7-96.8)	364.3<br>(290.3-446.6)	132.1<br>(107.6-161.7)	131.3<br>(75.3 to 204.5)	58.2<br>(22.3 to 101.4)
**Testicular cancer**	0.4<br>(0.4-0.5)	0<br>(0-0)	3.9<br>(2.9-4.9)	0<br>(0-0)	783.9<br>(544.3 to 1101.9)	0<br>(0 to 0)
**Thyroid cancer**	1.1<br>(0.9-1.4)	2.6<br>(2.2-3.5)	7.3<br>(5.5-9.2)	10.3<br>(8-13.2)	554.9<br>(339.5 to 812.8)	287.8<br>(183.4 to 434)
**Tracheal, bronchus, and lung cancer**	138.4<br>(113.2-164.7)	59.3<br>(49.8-69.7)	502.2<br>(395.4-615.3)	219.6<br>(177.4-267.7)	262.9<br>(169.2 to 396.2)	270.4<br>(185.3 to 380.7)
**Uterine cancer**	0<br>(0-0)	12.6<br>(9.9-15.4)	0<br>(0-0)	35.3<br>(27.4-51.3)	0<br>(0 to 0)	180<br>(104.8 to 307)

**Cause**	**Incidence rate in 1990 (95% UI)**		**Incidence rate in 2019 (95% UI)**		**Annual rate of change (95% UI)**	
	**Men**	**Women**	**Men**	**Women**	**Men**	**Women**
**Bladder cancer**	19.6<br>(16.5-22.9)	7.3<br>(6.1-8.7)	39.3<br>(31.4-47.7)	7.9<br>(6.3-9.9)	1<br>(0.55 to 1.64)	0.08<br>(-0.21 to 0.46)
**Brain and central nervous system cancer**	11.9<br>(8.3-17.6)	9.7<br>(7.3-13.3)	13.9<br>(8.7-18.4)	12.7<br>(9.2-16.2)	0.17<br>(-0.31 to 0.69)	0.31<br>(-0.26 to 0.89)
**Breast cancer**	0.5<br>(0.4-0.6)	50.6<br>(42.4-59.4)	3<br>(2.3-3.9)	112.4<br>(90.1-139.7)	4.97<br>(3.24 to 7.35)	1.22<br>(0.7 to 1.93)
**Cervical cancer**	0<br>(0-0)	24.9<br>(19.9-40.7)	0<br>(0-0)	26.6<br>(14.8-33.8)	0<br>(0 to 0)	0.07<br>(-0.54 to 0.55)
**Colon and rectum cancer**	53.8<br>(45.1-63.2)	46.6<br>(39.3-54.1)	170.1<br>(136.1-209.9)	96<br>(78-117.6)	2.16<br>(1.42 to 3.17)	1.06<br>(0.61 to 1.65)
**Esophageal cancer**	129.2<br>(84.5-155.7)	70.5<br>(38.8-83.8)	103.9<br>(76.8-128.7)	35.9<br>(22.9-45.4)	-0.2<br>(-0.39 to 0.13)	-0.49<br>(-0.61 to -0.29)

**Gallbladder and biliary tract cancer**	5.9<br>(4.6-10.2)	7.5<br>(5.9-13.5)	9.6<br>(6.5-12.1)	9.2<br>(5.5-12.1)	0.62<br>(-0.24 to 1.45)	0.23<br>(-0.51 to 0.82)
**Hodgkin lymphoma**	2.2<br>(1-3)	1.4<br>(0.6-1.9)	1.7<br>(1-2.2)	0.9<br>(0.5-1.1)	-0.26<br>(-0.5 to 0.31)	-0.39<br>(-0.57 to 0.05)
**Kidney cancer**	4<br>(3.2-4.9)	3<br>(2.5-3.4)	14.4<br>(11.2-18.1)	5.9<br>(4.7-7.3)	2.64<br>(1.6 to 4.11)	0.98<br>(0.53 to 1.57)
**Larynx cancer**	11.6<br>(9.5-13.7)	3.2<br>(2.7-3.8)	18.5<br>(14.5-23)	3.1<br>(2.4-3.7)	0.59<br>(0.2 to 1.16)	-0.06<br>(-0.28 to 0.23)
**Leukemia**	13.8<br>(10.8-17.9)	11.9<br>(9.7-14.5)	20<br>(15-25.5)	15.7<br>(11.8-20.1)	0.45<br>(0.02 to 1.02)	0.32<br>(-0.12 to 0.8)
**Lip and oral cavity cancer**	6.2<br>(5.1-7.4)	4.2<br>(3.6-5)	14.7<br>(11.6-18.2)	4.7<br>(3.8-5.7)	1.36<br>(0.73 to 2.28)	0.11<br>(-0.15 to 0.43)
**Liver cancer**	128.7<br>(103.3-159.6)	62.4<br>(51.3-76.9)	58.3<br>(46.6-72.1)	22.2<br>(17.9-27.1)	-0.55<br>(-0.67 to -0.37)	-0.64<br>(-0.74 to -0.52)
**Malignant skin melanoma**	1.2<br>(0.7-1.7)	1.2<br>(0.8-2)	2.4<br>(1.4-3.5)	2.6<br>(1.4-3.6)	1.07<br>(0.33 to 1.97)	1.13<br>(-0.01 to 2.16)
**Mesothelioma**	0.4<br>(0.3-0.6)	0.6<br>(0.4-0.9)	0.6<br>(0.5-0.7)	0.5<br>(0.3-0.6)	0.44<br>(-0.13 to 1.06)	-0.18<br>(-0.58 to 0.33)
**Multiple myeloma**	3.1<br>(2.3-4.6)	3.3<br>(2.5-4.9)	5.1<br>(3.4-6.8)	3.4<br>(2.4-4.4)	0.64<br>(-0.17 to 1.36)	0.03<br>(-0.45 to 0.44)
**Nasopharynx cancer**	13.7<br>(11.1-16.2)	7.3<br>(5.9-8.7)	20.5<br>(15.9-26)	6.8<br>(5.4-8.6)	0.5<br>(0.1 to 1.04)	-0.06<br>(-0.3 to 0.31)
**Non-Hodgkin lymphoma**	6.8<br>(5.5-8.1)	5<br>(4.2-5.8)	23.3<br>(18.4-29.1)	12<br>(9.6-14.9)	2.44<br>(1.59 to 3.66)	1.43<br>(0.83 to 2.17)
**Non-melanoma skin cancer**	17.7<br>(14.5-21.6)	15.2<br>(12.3-18.9)	72.6<br>(61.5-85.1)	49<br>(40.6-58.2)	3.1<br>(2.82 to 3.48)	2.22<br>(2.03 to 2.51)

**Other malignant neoplasms**	20.9<br>(16.5-25.7)	15.9<br>(13.2-19.1)	47.4<br>(35.4-59.4)	24.9<br>(19.8-30.9)	1.27<br>(0.58 to 2.15)	0.56<br>(0.14 to 1.12)
**Other pharynx cancer**	2.1<br>(1.8-2.5)	0.8<br>(0.7-0.9)	3.1<br>(2.5-3.9)	1<br>(0.8-1.2)	0.46<br>(0.08 to 0.96)	0.23<br>(-0.09 to 0.63)
**Ovarian cancer**	0<br>(0-0)	7.1<br>(5.5-11.2)	0<br>(0-0)	14.3<br>(9.8-18)	0<br>(0 to 0)	1.01<br>(0.03 to 1.95)
**Pancreatic cancer**	16.1<br>(13.1-19.2)	11.6<br>(9.9-13.5)	32.4<br>(25.8-39.6)	21.7<br>(17.5-26.6)	1.02<br>(0.49 to 1.76)	0.87<br>(0.45 to 1.38)
**Prostate cancer**	35.1<br>(26.8-42.3)	0<br>(0-0)	85.1<br>(65.8-113)	0<br>(0-0)	1.43<br>(0.81 to 2.34)	0<br>(0 to 0)
**Stomach cancer**	221.9<br>(185.2-260.5)	113.6<br>(96.2-131.7)	213.2<br>(169.9-261.4)	73.1<br>(59.5-89.4)	-0.04<br>(-0.27 to 0.26)	-0.36<br>(-0.5 to -0.18)
**Testicular cancer**	0.6<br>(0.5-0.8)	0<br>(0-0)	2.3<br>(1.7-2.9)	0<br>(0-0)	2.67<br>(1.68 to 3.99)	0<br>(0 to 0)
**Thyroid cancer**	1.6<br>(1.3-2)	3.6<br>(2.9-4.8)	4.3<br>(3.2-5.4)	5.7<br>(4.4-7.3)	1.72<br>(0.83 to 2.79)	0.58<br>(0.15 to 1.17)
**Tracheal, bronchus, and lung cancer**	194.9<br>(159.4-232)	80.7<br>(67.7-94.8)	293.9<br>(231.4-360.1)	121.5<br>(98.1-148)	0.51<br>(0.12 to 1.06)	0.51<br>(0.16 to 0.95)
**Uterine cancer**	0<br>(0-0)	17.2<br>(13.5-21)	0<br>(0-0)	19.5<br>(15.2-28.4)	0<br>(0 to 0)	0.14<br>(-0.17 to 0.65)

Note: Estimates are for populations aged 55 years and above among men and women in China. Rates are reported per 100000 person-years. Cancer types are listed alphabetically. Other malignant neoplasms are cancers without a detailed GBD cause separately listed. UI=uncertainty interval.

Table S7 The DALYs and DALY rate in the risks for 20 cancers among men in 1990 and 2019, and percentage change in DALYs, annual rate of change in DALY rate from 1990 to 2019

Risks	DALYs in 1990 (95% UI)	DALYs in 2019 (95% UI)	Percentage change in DALYs (95% UI)	DALY rate in 1990 (95% UI)	DALY rate in 2019 (95% UI)	Annual rate of change in DALY rate (95% UI)
**Bladder cancer**						
High fasting plasma glucose	12785 (1999-30191)	40055 (6568-92991)	213.3 (145.4 to 303.9)	10.63 (1.66-25.09)	12.69 (2.08-29.46)	0.19 (-0.06 to 0.54)
Smoking	122824 (94629-150764)	311877 (231366-400449)	153.9 (94.4 to 232.9)	102.08 (78.65-125.3)	98.79 (73.29-126.85)	-0.03 (-0.26 to 0.27)
**Breast cancer**						
Alcohol use	860 (625-1139)	7726 (5174-10433)	798.6 (509.8 to 1165.9)	0.71 (0.52-0.95)	2.45 (1.64-3.3)	2.42 (1.32 to 3.82)
Diet high in red meat	197 (65-298)	2478 (1130-3746)	1157.8 (688.6 to 2219)	0.16 (0.05-0.25)	0.78 (0.36-1.19)	3.79 (2.01 to 7.84)
Secondhand smoke	125 (29-241)	950 (215-1802)	658.7 (385.6 to 1038.3)	0.1 (0.02-0.2)	0.3 (0.07-0.57)	1.89 (0.85 to 3.34)
**Colon and rectum cancer**						
Alcohol use	81231 (58152-107365)	384828 (261759-533541)	373.7 (247.1 to 560.3)	67.51 (48.33-89.23)	121.9 (82.91-169)	0.81 (0.32 to 1.52)
Diet high in processed meat	4747 (2258-7768)	31742 (6869-59647)	568.7 (158.9 to 919.2)	3.95 (1.88-6.46)	10.05 (2.18-18.89)	1.55 (-0.01 to 2.88)
Diet high in red meat	23488 (3147-58861)	168658 (37465-335190)	618 (338 to 2185.7)	19.52 (2.62-48.92)	53.42 (11.87-106.17)	1.74 (0.67 to 7.71)
Diet low in calcium	147725 (111107-196012)	440453 (292864-620833)	198.2 (113.8 to 311.4)	122.78 (92.34-162.91)	139.52 (92.77-196.66)	0.14 (-0.19 to 0.57)

Diet low in fiber	13848 (5007-28646)	21819 (8815-51405)	57.6 (-12.6 to 176.8)	11.51 (4.16-23.81)	6.91 (2.79-16.28)	-0.4 (-0.67 to 0.05)
Diet low in milk	132517 (88231-179082)	535640 (346608-745316)	304.2 (207.7 to 430.1)	110.14 (73.33-148.84)	169.67 (109.79-236.09)	0.54 (0.17 to 1.02)
Diet low in whole grains	107838 (39969-146808)	447158 (165154-626933)	314.7 (214.4 to 441.2)	89.63 (33.22-122.02)	141.64 (52.31-198.59)	0.58 (0.2 to 1.06)
High body-mass index	21741 (4540-52179)	195138 (73968-368477)	797.5 (500.2 to 1804)	18.07 (3.77-43.37)	61.81 (23.43-116.72)	2.42 (1.29 to 6.26)
High fasting plasma glucose	41691 (7177-100147)	204594 (36016-497947)	390.7 (277.9 to 545.3)	34.65 (5.97-83.24)	64.81 (11.41-157.73)	0.87 (0.44 to 1.46)
Low physical activity	21333 (4739-45421)	80062 (16765-174347)	275.3 (167.1 to 422)	17.73 (3.94-37.75)	25.36 (5.31-55.23)	0.43 (0.02 to 0.99)
Smoking	174956 (111017-240698)	705341 (457502-1019230)	303.2 (200.3 to 442.4)	145.41 (92.27-200.05)	223.42 (144.92-322.85)	0.54 (0.14 to 1.07)
**Esophageal cancer**						
Alcohol use	591396 (363251-809072)	1092482 (756484-1498491)	84.7 (33.2 to 170.9)	491.53 (301.91-672.45)	346.05 (239.62-474.66)	-0.3 (-0.49 to 0.03)
Chewing tobacco	15395 (6999-27039)	27042 (12652-46830)	75.6 (3.3 to 205.6)	12.8 (5.82-22.47)	8.57 (4.01-14.83)	-0.33 (-0.61 to 0.16)
Diet low in fruits	373685 (109071-695450)	264672 (38755-732741)	-29.2 (-79.7 to 40.1)	310.58 (90.65-578.01)	83.84 (12.28-232.1)	-0.73 (-0.92 to -0.47)
Diet low in vegetables	147774 (7113-319811)	13100 (6386-30768)	-91.1 (-95.7 to 1.6)	122.82 (5.91-265.81)	4.15 (2.02-9.75)	-0.97 (-0.98 to -0.61)
High body-mass index	142288 (13645-418724)	517607 (76076-1192708)	263.8 (130.4 to 766.1)	118.26 (11.34-348.02)	163.96 (24.1-377.8)	0.39 (-0.12 to 2.3)
Smoking	1336236	2172540	62.6	1110.59	688.17	-0.38

	(824908-1634936)	(1638397-2754679)	(22.2 to 132.8)	(685.61-1358.85)	(518.98-872.57)	(-0.53 to -0.11)
**Gallbladder and biliary tract cancer**						
High body-mass index	2619 (289-7900)	19075 (3144-44654)	628.4 (252.7 to 1610.4)	2.18 (0.24-6.57)	6.04 (1-14.14)	1.78 (0.34 to 5.52)
Kidney cancer						
High body-mass index	2099 (423-5240)	26265 (9897-49318)	1151.1 (679.3 to 2726.2)	1.74 (0.35-4.35)	8.32 (3.14-15.62)	3.77 (1.97 to 9.77)
Occupational exposure to trichloroethylene	41 (9-79)	234 (49-454)	469.2 (289.6 to 706.3)	0.03 (0.01-0.07)	0.07 (0.02-0.14)	1.17 (0.48 to 2.07)
Smoking	14495 (9362-20061)	82663 (55482-113955)	470.3 (296.1 to 722.1)	12.05 (7.78-16.67)	26.18 (17.57-36.1)	1.17 (0.51 to 2.13)
**Larynx cancer**						
Alcohol use	39170 (21689-57211)	87820 (50459-127306)	124.2 (61.6 to 221.3)	32.56 (18.03-47.55)	27.82 (15.98-40.33)	-0.15 (-0.38 to 0.22)
Occupational exposure to asbestos	991 (453-1871)	3180 (1448-5387)	221 (71.3 to 427.3)	0.82 (0.38-1.56)	1.01 (0.46-1.71)	0.22 (-0.35 to 1.01)
Occupational exposure to sulfuric acid	6879 (2733-12964)	12432 (5078-24436)	80.7 (32.6 to 145.7)	5.72 (2.27-10.78)	3.94 (1.61-7.74)	-0.31 (-0.49 to -0.06)
Smoking	140658 (113773-167707)	277080 (217037-342169)	97 (48.1 to 164.1)	116.91 (94.56-139.39)	87.77 (68.75-108.39)	-0.25 (-0.44 to 0.01)
**Leukemia**						
High body-mass index	3478 (658-8943)	15869 (5369-32183)	356.3 (194.2 to 907.9)	2.89 (0.55-7.43)	5.03 (1.7-10.19)	0.74 (0.12 to 2.84)
Occupational exposure to benzene	662 (191-1162)	1392 (393-2457)	110.1 (50 to 194.6)	0.55 (0.16-0.97)	0.44 (0.12-0.78)	-0.2 (-0.43 to 0.12)

Occupational exposure to formaldehyde	327 (202-493)	678 (406-1014)	107.2 (42.5 to 198.1)	0.27 (0.17-0.41)	0.21 (0.13-0.32)	-0.21 (-0.46 to 0.14)
Smoking	97485 (58452-141406)	196096 (117789-282874)	101.2 (35.6 to 186.8)	81.02 (48.58-117.53)	62.12 (37.31-89.6)	-0.23 (-0.48 to 0.09)
**Lip and oral cavity cancer**						
Alcohol use	32961 (24583-42658)	153305 (112965-202117)	365.1 (229.8 to 558.4)	27.4 (20.43-35.45)	48.56 (35.78-64.02)	0.77 (0.26 to 1.51)
Chewing tobacco	657 (278-1214)	2963 (1322-5463)	351 (157.4 to 670.5)	0.55 (0.23-1.01)	0.94 (0.42-1.73)	0.72 (-0.02 to 1.94)
Smoking	44029 (34406-54095)	182540 (139677-231574)	314.6 (201.2 to 474.2)	36.59 (28.6-44.96)	57.82 (44.24-73.35)	0.58 (0.15 to 1.19)
**Liver cancer**						
Alcohol use	253402 (162663-379907)	262888 (176336-380940)	3.7 (-24.7 to 47.7)	210.61 (135.19-315.75)	83.27 (55.86-120.67)	-0.6 (-0.71 to -0.44)
Drug use	247259 (187197-320162)	251142 (184994-321942)	1.6 (-26.5 to 40.9)	205.5 (155.59-266.1)	79.55 (58.6-101.98)	-0.61 (-0.72 to -0.46)
High body-mass index	112579 (18242-304688)	227927 (65438-500744)	102.5 (30 to 378.7)	93.57 (15.16-253.24)	72.2 (20.73-158.62)	-0.23 (-0.5 to 0.82)
High fasting plasma glucose	9213 (1738-23433)	11538 (2331-28976)	25.2 (-6.5 to 74)	7.66 (1.44-19.48)	3.65 (0.74-9.18)	-0.52 (-0.64 to -0.34)
Smoking	658948 (341393-997762)	605053 (318983-907545)	-8.2 (-35.3 to 33.1)	547.67 (283.74-829.27)	191.66 (101.04-287.47)	-0.65 (-0.75 to -0.49)
**Mesothelioma**						
Occupational exposure to asbestos	5212 (3629-8485)	19522 (14901-25730)	274.6 (109.2 to 467.3)	4.33 (3.02-7.05)	6.18 (4.72-8.15)	0.43 (-0.2 to 1.16)
**Multiple myeloma**						

High body-mass index	785 (109-2409)	5250 (1179-12774)	568.7 (247.8 to 1460)	0.65 (0.09-2)	1.66 (0.37-4.05)	1.55 (0.33 to 4.95)
**Nasopharynx cancer**						
Alcohol use	110939 (81222-144576)	173953 (125976-225424)	56.8 (13.9 to 115.2)	92.21 (67.51-120.16)	55.1 (39.9-71.41)	-0.4 (-0.57 to -0.18)
Occupational exposure to formaldehyde	1050 (369-2090)	1555 (646-2846)	48 (0.3 to 131.6)	0.87 (0.31-1.74)	0.49 (0.2-0.9)	-0.44 (-0.62 to -0.12)
Smoking	100824 (69968-134298)	148473 (100701-203861)	47.3 (7.5 to 101.9)	83.8 (58.15-111.62)	47.03 (31.9-64.57)	-0.44 (-0.59 to -0.23)
**Non-Hodgkin lymphoma**						
High body-mass index	2002 (331-5568)	18459 (5351-41482)	822.2 (515.5 to 1906.1)	1.66 (0.28-4.63)	5.85 (1.69-13.14)	2.51 (1.35 to 6.65)
Other pharynx cancer						
Alcohol use	15930 (11758-20481)	40742 (28741-53770)	155.8 (79.3 to 257.5)	13.24 (9.77-17.02)	12.91 (9.1-17.03)	-0.03 (-0.32 to 0.36)
Smoking	24756 (20227-29911)	56918 (43444-72058)	129.9 (65.5 to 212.9)	20.58 (16.81-24.86)	18.03 (13.76-22.83)	-0.12 (-0.37 to 0.19)
**Pancreatic cancer**						
High body-mass index	3814 (-11-12755)	37427 (-168-103303)	881.4 (553.6 to 2039.1)	3.17 (-0.01-10.6)	11.86 (-0.05-32.72)	2.74 (1.49 to 7.15)
High fasting plasma glucose	16362 (2865-40678)	86759 (14902-212154)	430.3 (277.5 to 643.6)	13.6 (2.38-33.81)	27.48 (4.72-67.2)	1.02 (0.44 to 1.83)
Smoking	91458 (70475-115296)	408066 (304888-529984)	346.2 (217 to 521.6)	76.01 (58.57-95.83)	129.26 (96.58-167.88)	0.7 (0.21 to 1.37)
**Prostate cancer**						
Smoking	38000	92885	144.4	31.58	29.42	-0.07

	(17933-59320)	(43248-149606)	(80.2 to 246.7)	(14.9-49.3)	(13.7-47.39)	(-0.31 to 0.32)
**Stomach cancer**						
Diet high in sodium	323860 (7020-1200356)	472380 (10125-1762868)	45.9 (7.6 to 92.4)	269.17 (5.83-997.66)	149.63 (3.21-558.41)	-0.44 (-0.59 to -0.27)
Smoking	1090487 (812207-1389365)	1667273 (1233666-2126949)	52.9 (13.7 to 107.5)	906.34 (675.05-1154.75)	528.13 (390.78-673.73)	-0.42 (-0.57 to -0.21)
**Thyroid cancer**						
High body-mass index	717 (97-2082)	5479 (1332-12724)	664 (380.3 to 1636.9)	0.6 (0.08-1.73)	1.74 (0.42-4.03)	1.91 (0.83 to 5.62)
**Tracheal, bronchus, and lung cancer**						
Ambient particulate matter pollution	381671 (167324-658312)	2180066 (1473769-3001692)	471.2 (211.5 to 1133.5)	317.22 (139.07-547.14)	690.56 (466.83-950.82)	1.18 (0.19 to 3.7)
Diet low in fruits	169386 (54712-270628)	335828 (86434-561374)	98.3 (20.6 to 199.4)	140.78 (45.47-224.93)	106.38 (27.38-177.82)	-0.24 (-0.54 to 0.14)
High fasting plasma glucose	192980 (30451-475096)	679282 (112979-1668784)	252 (150.1 to 383.7)	160.39 (25.31-394.87)	215.17 (35.79-528.6)	0.34 (-0.05 to 0.84)
Household air pollution from solid fuels	690623 (410995-1021762)	416438 (181330-791662)	-39.7 (-70.4 to 6.9)	574 (341.59-849.22)	131.91 (57.44-250.77)	-0.77 (-0.89 to -0.59)
Occupational exposure to arsenic	19466 (6908-32716)	51070 (17669-86895)	162.4 (82.7 to 265.2)	16.18 (5.74-27.19)	16.18 (5.6-27.52)	0 (-0.3 to 0.39)
Occupational exposure to asbestos	61913 (31524-111419)	286422 (152526-479704)	362.6 (163.3 to 670.7)	51.46 (26.2-92.6)	90.73 (48.31-151.95)	0.76 (0 to 1.94)
Occupational exposure to beryllium	847 (553-1184)	2053 (1364-2916)	142.2 (68.7 to 241.3)	0.7 (0.46-0.98)	0.65 (0.43-0.92)	-0.08 (-0.36 to 0.3)

Occupational exposure to cadmium	1907 (1275-2619)	4991 (3315-6768)	161.8 (84.1 to 269.3)	1.58 (1.06-2.18)	1.58 (1.05-2.14)	0 (-0.3 to 0.41)
Occupational exposure to chromium	3648 (2758-4769)	10177 (7339-13809)	179 (94.3 to 294.4)	3.03 (2.29-3.96)	3.22 (2.32-4.37)	0.06 (-0.26 to 0.5)
Occupational exposure to diesel engine exhaust	43884 (31291-57907)	127417 (92744-170193)	190.3 (107.9 to 313.1)	36.47 (26.01-48.13)	40.36 (29.38-53.91)	0.11 (-0.21 to 0.57)
Occupational exposure to nickel	22101 (4462-50543)	53200 (11065-123474)	140.7 (69 to 237.7)	18.37 (3.71-42.01)	16.85 (3.51-39.11)	-0.08 (-0.36 to 0.29)
Occupational exposure to polycyclic aromatic hydrocarbons	12723 (8783-17092)	35165 (24148-48804)	176.4 (94.7 to 290.9)	10.57 (7.3-14.21)	11.14 (7.65-15.46)	0.05 (-0.26 to 0.49)
Occupational exposure to silica	114187 (51492-184253)	277535 (121372-453653)	143.1 (71.3 to 240.7)	94.9 (42.8-153.14)	87.91 (38.45-143.7)	-0.07 (-0.35 to 0.3)
Residential radon	122988 (22311-259016)	354507 (65690-758704)	188.2 (104.3 to 298.5)	102.22 (18.54-215.28)	112.29 (20.81-240.33)	0.1 (-0.22 to 0.52)
Secondhand smoke	200864 (114960-311402)	588279 (333552-919162)	192.9 (101.3 to 337)	166.94 (95.55-258.82)	186.34 (105.66-291.15)	0.12 (-0.23 to 0.67)
Smoking	2696387 (2205582-3230714)	7948174 (6222560-9896890)	194.8 (111 to 300.6)	2241.05 (1833.13-2685.15)	2517.66 (1971.06-3134.94)	0.12 (-0.2 to 0.53)

Note: Estimates are for populations aged 55 years and above among men in China. Rates are reported per 100000 person-years. Cancer types and risks are listed alphabetically. DALY=disability-adjusted life-year, UI=uncertainty interval.

Table S8 The DALYs and DALY rate in the risks for 22 cancers among women in 1990 and 2019, and percentage change in DALYs, annual rate of change in DALY rate from 1990 to 2019

Risks	DALYs in 1990 (95% UI)	DALYs in 2019 (95% UI)	Percentage change in DALYs (95% UI)	DALY rate in 1990 (95% UI)	DALY rate in 2019 (95% UI)	Annual rate of change in DALY rate (95% UI)
**Bladder cancer**						
High fasting plasma glucose	5860 (1037-14513)	9806 (1662-23194)	67.4 (25 to 125.5)	4.31 (0.76-10.67)	2.78 (0.47-6.58)	-0.35 (-0.52 to -0.13)
Smoking	13736 (8280-19353)	17326 (10620-25194)	26.1 (-13.5 to 85.2)	10.1 (6.09-14.22)	4.91 (3.01-7.14)	-0.51 (-0.67 to -0.29)
**Breast cancer**						
Alcohol use	12246 (8470-16684)	37690 (25098-51911)	207.8 (103.5 to 381.2)	9 (6.22-12.26)	10.69 (7.12-14.72)	0.19 (-0.21 to 0.86)
Diet high in red meat	17642 (6066-26732)	72606 (32899-105424)	311.6 (182.3 to 627.9)	12.97 (4.46-19.65)	20.59 (9.33-29.9)	0.59 (0.09 to 1.81)
High body-mass index	45198 (8115-109612)	251768 (71756-507947)	457 (281.1 to 980.7)	33.22 (5.96-80.56)	71.4 (20.35-144.05)	1.15 (0.47 to 3.17)
High fasting plasma glucose	35926 (6796-82776)	105013 (19529-245563)	192.3 (128.8 to 282.6)	26.4 (4.99-60.84)	29.78 (5.54-69.64)	0.13 (-0.12 to 0.48)
Low physical activity	5676 (2664-10469)	14486 (7094-26678)	155.2 (91.1 to 243.5)	4.17 (1.96-7.69)	4.11 (2.01-7.57)	-0.02 (-0.26 to 0.33)
Secondhand smoke	24421 (5783-42570)	57265 (14064-101864)	134.5 (79.6 to 209.9)	17.95 (4.25-31.29)	16.24 (3.99-28.89)	-0.1 (-0.31 to 0.2)
Smoking	6482 (3846-10226)	21890 (14521-31417)	237.7 (121.4 to 426.6)	4.76 (2.83-7.52)	6.21 (4.12-8.91)	0.3 (-0.15 to 1.03)
**Cervical cancer**						

Smoking	58307 (25478-112250)	94995 (36983-178480)	62.9 (-30.4 to 160.1)	42.85 (18.72-82.5)	26.94 (10.49-50.61)	-0.37 (-0.73 to 0)
Unsafe sex	384588 (303304-616502)	804412 (461315-1026731)	109.2 (-9.7 to 201.8)	282.65 (222.91-453.09)	228.12 (130.82-291.17)	-0.19 (-0.65 to 0.16)
**Colon and rectum cancer**						
Alcohol use	9633 (6104-13856)	32560 (20341-48407)	238 (116.5 to 430.2)	7.08 (4.49-10.18)	9.23 (5.77-13.73)	0.3 (-0.16 to 1.05)
Diet high in processed meat	4447 (1842-7577)	21071 (3785-39718)	373.8 (76.7 to 604.5)	3.27 (1.35-5.57)	5.98 (1.07-11.26)	0.83 (-0.32 to 1.72)
Diet high in red meat	20061 (2485-49370)	95722 (25060-189500)	377.2 (201.3 to 1615.5)	14.74 (1.83-36.28)	27.15 (7.11-53.74)	0.84 (0.16 to 5.62)
Diet low in calcium	120042 (89928-154963)	230631 (145366-338790)	92.1 (40.7 to 159.5)	88.22 (66.09-113.89)	65.4 (41.22-96.08)	-0.26 (-0.46 to 0)
Diet low in fiber	13498 (4824-27058)	14991 (5716-36734)	11.1 (-37.1 to 84)	9.92 (3.55-19.89)	4.25 (1.62-10.42)	-0.57 (-0.76 to -0.29)
Diet low in milk	115537 (77358-155871)	309776 (201432-419041)	168.1 (111.3 to 246.9)	84.91 (56.85-114.56)	87.85 (57.12-118.84)	0.03 (-0.18 to 0.34)
Diet low in whole grains	92154 (34163-125925)	253611 (94050-354702)	175.2 (114.1 to 255.9)	67.73 (25.11-92.55)	71.92 (26.67-100.59)	0.06 (-0.17 to 0.37)
High body-mass index	8267 (1831-21529)	46479 (16035-100750)	462.3 (297.7 to 955.4)	6.08 (1.35-15.82)	13.18 (4.55-28.57)	1.17 (0.53 to 3.07)
High fasting plasma glucose	39887 (7361-96438)	115159 (20783-275751)	188.7 (126.7 to 274.1)	29.31 (5.41-70.88)	32.66 (5.89-78.2)	0.11 (-0.13 to 0.44)
Low physical activity	28485 (7496-55781)	74070 (20115-149763)	160 (91.6 to 257.4)	20.93 (5.51-41)	21.01 (5.7-42.47)	0 (-0.26 to 0.38)

Smoking	14337 (5822-23320)	49544 (25306-75593)	245.6 (131.6 to 436.9)	10.54 (4.28-17.14)	14.05 (7.18-21.44)	0.33 (-0.11 to 1.07)
**Esophageal cancer**						
Alcohol use	46714 (23965-74697)	53375 (28557-83085)	14.3 (-26.7 to 90.7)	34.33 (17.61-54.9)	15.14 (8.1-23.56)	-0.56 (-0.72 to -0.26)
Chewing tobacco	3258 (1276-6035)	2848 (1301-5421)	-12.6 (-54 to 67.4)	2.39 (0.94-4.44)	0.81 (0.37-1.54)	-0.66 (-0.82 to -0.35)
Diet low in fruits	200682 (61444-376873)	70558 (9637-192153)	-64.8 (-90.6 to -30.3)	147.49 (45.16-276.98)	20.01 (2.73-54.49)	-0.86 (-0.96 to -0.73)
Diet low in vegetables	73780 (3702-164171)	3781 (1780-8758)	-94.9 (-97.3 to -43.5)	54.22 (2.72-120.65)	1.07 (0.5-2.48)	-0.98 (-0.99 to -0.78)
High body-mass index	84085 (3491-264583)	151147 (7650-377110)	79.8 (22.7 to 268.5)	61.8 (2.57-194.45)	42.86 (2.17-106.94)	-0.31 (-0.53 to 0.42)
Smoking	119549 (66781-163972)	100317 (68281-136958)	-16.1 (-43.6 to 31.9)	87.86 (49.08-120.51)	28.45 (19.36-38.84)	-0.68 (-0.78 to -0.49)
**Gallbladder and biliary tract cancer**						
High body-mass index	8484 (2015-21546)	43353 (16688-84713)	411 (107.3 to 1017.7)	6.24 (1.48-15.84)	12.29 (4.73-24.02)	0.97 (-0.2 to 3.31)
**Kidney cancer**						
High body-mass index	2561 (650-5701)	17222 (7325-31363)	572.5 (365.7 to 1219)	1.88 (0.48-4.19)	4.88 (2.08-8.89)	1.59 (0.8 to 4.09)
Occupational exposure to trichloroethylene	30 (6-58)	116 (24-216)	289.3 (194 to 420)	0.02 (0-0.04)	0.03 (0.01-0.06)	0.5 (0.13 to 1.01)
Smoking	1035 (510-1723)	4758 (2744-7095)	359.6 (209.7 to 618.9)	0.76 (0.37-1.27)	1.35 (0.78-2.01)	0.77 (0.19 to 1.77)
**Larynx cancer**						

Alcohol use	1449 (507-2614)	2467 (1056-4162)	70.3 (7.9 to 203.8)	1.06 (0.37-1.92)	0.7 (0.3-1.18)	-0.34 (-0.58 to 0.17)
Occupational exposure to asbestos	502 (205-1000)	642 (257-1120)	27.8 (-42.3 to 135.7)	0.37 (0.15-0.74)	0.18 (0.07-0.32)	-0.51 (-0.78 to -0.09)
Occupational exposure to sulfuric acid	1903 (753-3539)	2563 (1058-4954)	34.7 (2.9 to 78.5)	1.4 (0.55-2.6)	0.73 (0.3-1.4)	-0.48 (-0.6 to -0.31)
Smoking	14957 (10429-19654)	17728 (11692-24381)	18.5 (-14.8 to 66.2)	10.99 (7.66-14.44)	5.03 (3.32-6.91)	-0.54 (-0.67 to -0.36)
**Leukemia**						
High body-mass index	5855 (1095-15482)	21390 (6365-45642)	265.3 (142.9 to 643)	4.3 (0.8-11.38)	6.07 (1.81-12.94)	0.41 (-0.06 to 1.87)
Occupational exposure to benzene	472 (138-811)	1135 (307-2017)	140.5 (61.6 to 230.6)	0.35 (0.1-0.6)	0.32 (0.09-0.57)	-0.07 (-0.38 to 0.28)
Occupational exposure to formaldehyde	240 (148-357)	500 (306-755)	108.3 (39.5 to 198.7)	0.18 (0.11-0.26)	0.14 (0.09-0.21)	-0.2 (-0.46 to 0.15)
Smoking	9569 (3479-18881)	20455 (9210-37230)	113.8 (29.8 to 265.6)	7.03 (2.56-13.88)	5.8 (2.61-10.56)	-0.18 (-0.5 to 0.41)
**Lip and oral cavity cancer**						
Alcohol use	3505 (2080-5193)	8713 (5341-13251)	148.6 (56.8 to 295.2)	2.58 (1.53-3.82)	2.47 (1.51-3.76)	-0.04 (-0.39 to 0.52)
Chewing tobacco	548 (272-982)	1029 (529-1781)	87.7 (3.2 to 234.9)	0.4 (0.2-0.72)	0.29 (0.15-0.51)	-0.28 (-0.6 to 0.29)
Smoking	3569 (2163-5365)	8470 (5732-11808)	137.3 (61.8 to 254.7)	2.62 (1.59-3.94)	2.4 (1.63-3.35)	-0.08 (-0.38 to 0.37)
**Liver cancer**						
Alcohol use	102115	90505	-11.4	75.05	25.67	-0.66

	(69886-144015)	(61938-124562)	(-36.3 to 22.1)	(51.36-105.84)	(17.56-35.32)	(-0.75 to -0.53)
Drug use	201174	195955	-2.6	147.85	55.57	-0.62
	(118494-306314)	(129599-275936)	(-34.3 to 48.2)	(87.09-225.12)	(36.75-78.25)	(-0.75 to -0.43)
High body-mass index	39829	62438	56.8	29.27	17.71	-0.4
	(4207-117330)	(9252-154761)	(3.1 to 223.4)	(3.09-86.23)	(2.62-43.89)	(-0.6 to 0.25)
High fasting plasma glucose	11363	10165	-10.5	8.35	2.88	-0.65
	(1989-27673)	(1897-24237)	(-35.3 to 24.1)	(1.46-20.34)	(0.54-6.87)	(-0.75 to -0.52)
Smoking	28565	29088	1.8	20.99	8.25	-0.61
	(10394-52062)	(12551-47282)	(-36.8 to 73.5)	(7.64-38.26)	(3.56-13.41)	(-0.76 to -0.33)
**Mesothelioma**						
Occupational exposure to asbestos	8672	16179	86.6	6.37	4.59	-0.28
	(5674-14415)	(9492-21486)	(-10.9 to 223.6)	(4.17-10.59)	(2.69-6.09)	(-0.66 to 0.25)
**Multiple myeloma**						
High body-mass index	1098	4809	337.9	0.81	1.36	0.69
	(189-2997)	(1260-11097)	(141.6 to 797.4)	(0.14-2.2)	(0.36-3.15)	(-0.07 to 2.46)
**Nasopharynx cancer**						
Alcohol use	12761	14925	17	9.38	4.23	-0.55
	(6842-20026)	(8593-22416)	(-26.1 to 87.1)	(5.03-14.72)	(2.44-6.36)	(-0.71 to -0.28)
Occupational exposure to formaldehyde	440	526	19.6	0.32	0.15	-0.54
	(170-835)	(214-955)	(-14.2 to 77.8)	(0.12-0.61)	(0.06-0.27)	(-0.67 to -0.31)
Smoking	4829	5358	11	3.55	1.52	-0.57
	(2574-7857)	(3042-8253)	(-29.1 to 80.3)	(1.89-5.77)	(0.86-2.34)	(-0.73 to -0.3)
**Non-Hodgkin lymphoma**						
High body-mass index	1376	8595	524.8	1.01	2.44	1.41
	(178-3922)	(1308-20602)	(333 to 1104.1)	(0.13-2.88)	(0.37-5.84)	(0.67 to 3.65)
Other pharynx cancer						

Alcohol use	1075 (596-1634)	1807 (1042-2729)	68.1 (5.3 to 174.9)	0.79 (0.44-1.2)	0.51 (0.3-0.77)	-0.35 (-0.59 to 0.06)
Smoking	1603 (1039-2343)	2662 (1885-3640)	66 (11.7 to 156.3)	1.18 (0.76-1.72)	0.75 (0.53-1.03)	-0.36 (-0.57 to -0.01)
**Ovarian cancer**						
High body-mass index	1081 (-27-3414)	9968 (-234-26312)	821.8 (381 to 1828.6)	0.79 (-0.02-2.51)	2.83 (-0.07-7.46)	2.56 (0.86 to 6.44)
High fasting plasma glucose	7168 (1271-19123)	33244 (5664-83014)	363.8 (138.3 to 582.1)	5.27 (0.93-14.05)	9.43 (1.61-23.54)	0.79 (-0.08 to 1.63)
Occupational exposure to asbestos	2067 (855-4785)	7236 (2514-13153)	250.1 (7.5 to 619.5)	1.52 (0.63-3.52)	2.05 (0.71-3.73)	0.35 (-0.59 to 1.78)
**Pancreatic cancer**						
High body-mass index	4368 (850-10629)	37794 (10790-80752)	765.2 (499.8 to 1545.5)	3.21 (0.62-7.81)	10.72 (3.06-22.9)	2.34 (1.31 to 5.35)
High fasting plasma glucose	12969 (2040-31223)	57573 (8932-140458)	343.9 (243.7 to 471.5)	9.53 (1.5-22.95)	16.33 (2.53-39.83)	0.71 (0.33 to 1.21)
Smoking	13135 (8676-18774)	69574 (49681-92557)	429.7 (260.4 to 706.4)	9.65 (6.38-13.8)	19.73 (14.09-26.25)	1.04 (0.39 to 2.11)
**Stomach cancer**						
Diet high in sodium	161143 (3681-602200)	176037 (3905-682994)	9.2 (-18.1 to 39.6)	118.43 (2.71-442.58)	49.92 (1.11-193.69)	-0.58 (-0.68 to -0.46)
Smoking	47672 (28311-71711)	67339 (45486-94007)	41.3 (-6.3 to 113.9)	35.04 (20.81-52.7)	19.1 (12.9-26.66)	-0.45 (-0.64 to -0.17)
**Thyroid cancer**						
High body-mass index	991 (232-2388)	3504 (1381-7012)	253.5 (138.8 to 592.9)	0.73 (0.17-1.75)	0.99 (0.39-1.99)	0.36 (-0.08 to 1.67)

**Tracheal, bronchus, and lung cancer**						
Ambient particulate matter pollution	123445 (56467-217452)	866752 (611231-1168817)	602.1 (292.4 to 1383.3)	90.72 (41.5-159.81)	245.8 (173.34-331.46)	1.71 (0.51 to 4.72)
Diet low in fruits	71235 (23790-112519)	134380 (37082-211423)	88.6 (15.4 to 180.5)	52.35 (17.48-82.69)	38.11 (10.52-59.96)	-0.27 (-0.55 to 0.08)
High fasting plasma glucose	88422 (17042-221322)	278294 (51642-686327)	214.7 (143.4 to 312)	64.98 (12.52-162.66)	78.92 (14.64-194.63)	0.21 (-0.06 to 0.59)
Household air pollution from solid fuels	331155 (215263-470664)	244149 (115569-425021)	-26.3 (-61 to 24)	243.38 (158.2-345.91)	69.24 (32.77-120.53)	-0.72 (-0.85 to -0.52)
Occupational exposure to arsenic	8269 (2991-14033)	25632 (9155-44118)	210 (133.1 to 322.6)	6.08 (2.2-10.31)	7.27 (2.6-12.51)	0.2 (-0.1 to 0.63)
Occupational exposure to asbestos	29838 (16235-53298)	80780 (40922-126625)	170.7 (22.6 to 385.9)	21.93 (11.93-39.17)	22.91 (11.61-35.91)	0.04 (-0.53 to 0.87)
Occupational exposure to beryllium	355 (239-481)	1093 (738-1499)	208.2 (130.6 to 317.9)	0.26 (0.18-0.35)	0.31 (0.21-0.42)	0.19 (-0.11 to 0.61)
Occupational exposure to cadmium	802 (553-1087)	2516 (1729-3515)	213.7 (132.8 to 332)	0.59 (0.41-0.8)	0.71 (0.49-1)	0.21 (-0.1 to 0.67)
Occupational exposure to chromium	1482 (1122-1902)	4903 (3651-6512)	230.9 (145.6 to 348.6)	1.09 (0.82-1.4)	1.39 (1.04-1.85)	0.28 (-0.05 to 0.73)
Occupational exposure to diesel engine exhaust	13650 (10009-17704)	47894 (35253-63046)	250.9 (161.2 to 375.1)	10.03 (7.36-13.01)	13.58 (10-17.88)	0.35 (0.01 to 0.83)
Occupational exposure to nickel	8713 (1903-19243)	25087 (4808-56305)	187.9 (115.8 to 289.6)	6.4 (1.4-14.14)	7.11 (1.36-15.97)	0.11 (-0.17 to 0.5)

Occupational exposure to polycyclic aromatic hydrocarbons	5115 (3594-6842)	16807 (11924-23126)	228.6 (145.8 to 353.1)	3.76 (2.64-5.03)	4.77 (3.38-6.56)	0.27 (-0.05 to 0.75)
Occupational exposure to silica	41574 (18930-65591)	110057 (45819-180259)	164.7 (97.1 to 256.6)	30.55 (13.91-48.21)	31.21 (12.99-51.12)	0.02 (-0.24 to 0.38)
Residential radon	50695 (9148-107729)	153562 (25780-325826)	202.9 (133.5 to 297.3)	37.26 (6.72-79.17)	43.55 (7.31-92.4)	0.17 (-0.1 to 0.53)
Secondhand smoke	166692 (96027-250025)	458641 (275227-696690)	175.1 (109.6 to 260.9)	122.51 (70.57-183.75)	130.07 (78.05-197.57)	0.06 (-0.19 to 0.39)
Smoking	264340 (194150-346156)	1029794 (813087-1296152)	289.6 (175.3 to 455.9)	194.27 (142.69-254.4)	292.04 (230.58-367.57)	0.5 (0.06 to 1.14)
**Uterine cancer**						
High body-mass index	20354 (5453-43134)	54702 (24465-98887)	168.8 (76.6 to 442.3)	14.96 (4.01-31.7)	15.51 (6.94-28.04)	0.04 (-0.32 to 1.09)

Note: Estimates are for populations aged 55 years and above among women in China. Rates are reported per 100000 person-years. Cancer types and risks are listed alphabetically. DALY=disability-adjusted life-year, UI=uncertainty interval.

Figure S1 The changing trend of incidence rate (A) and the rank of incident cases (B) for 29 cancers in China from 1990 to 2019

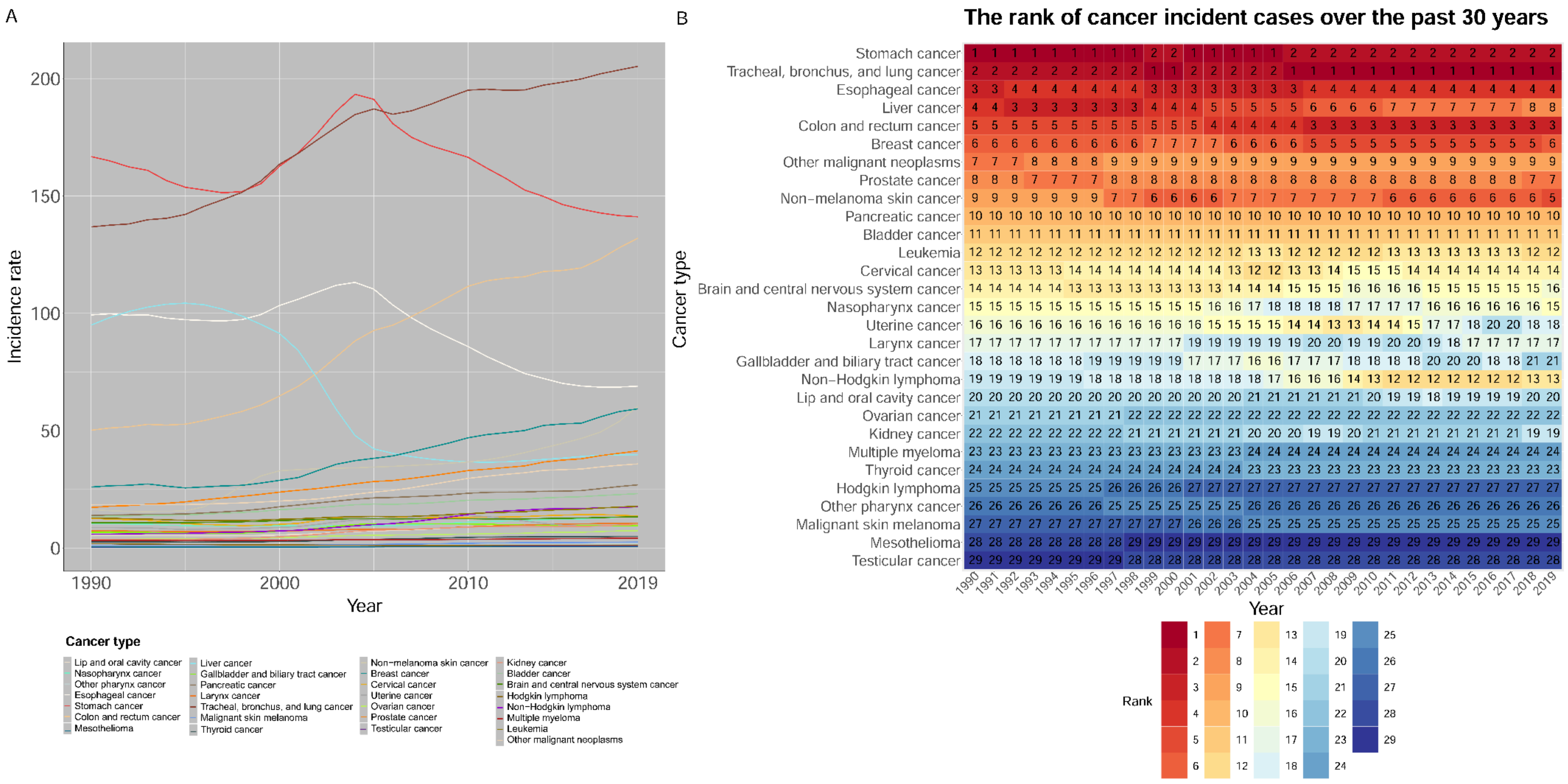

Note: Estimates are for populations aged 55 years and above, both sexes in China. Rates are reported per 100000 person-years.

Figure S2 Incident cases, deaths and DALYs (A, B, C) of 29 cancers in 4 age groups (55−64 years, 65−74 years, 75−84 years, 85+ years) and the proportional incident cases, deaths, DALYs (D, E, F) in the four age groups in China in 2019

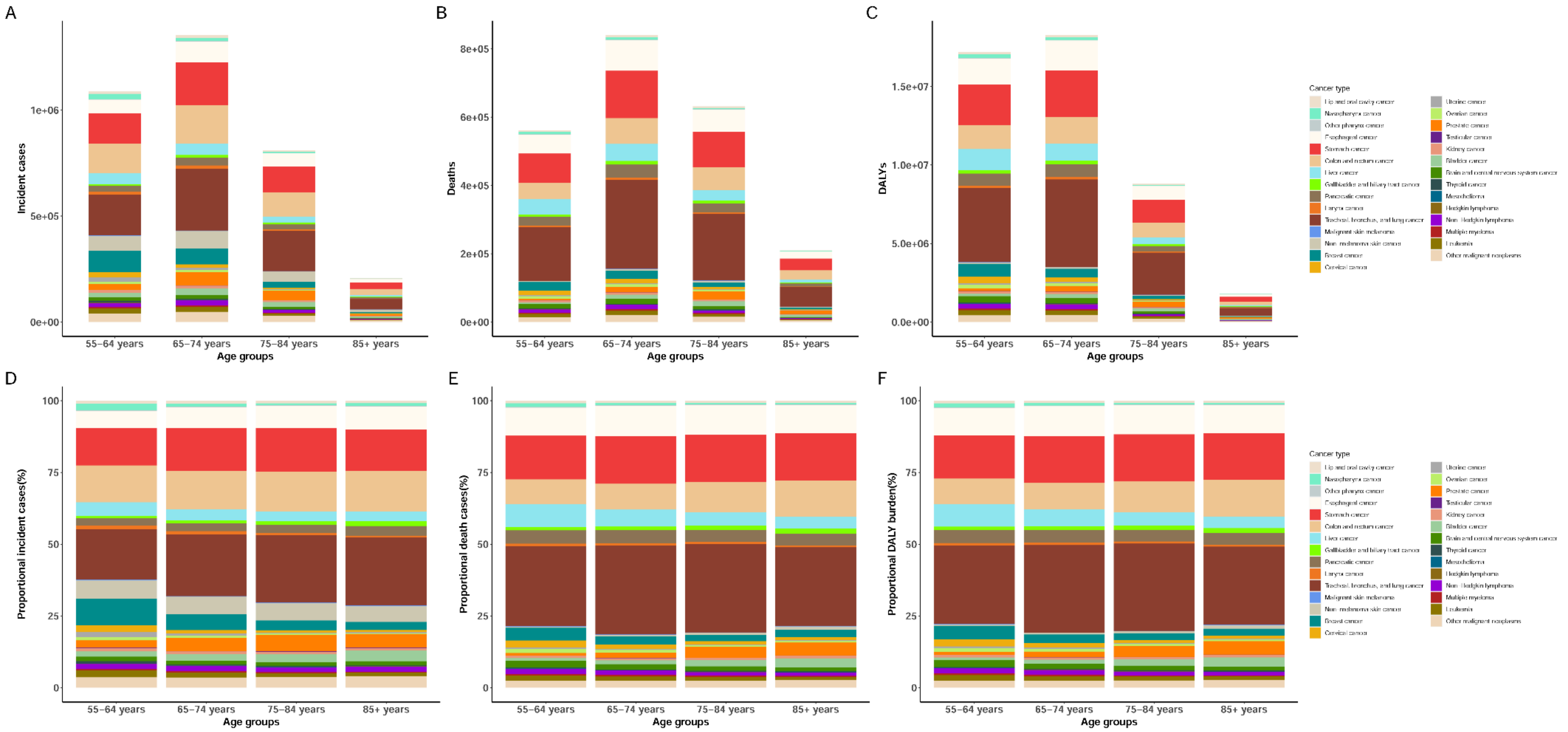

Note: Estimates are for populations aged 55 years and above, both sexes in China. DALY = disability-adjusted life-year.

Figure S3 Incident cases, DALYs (A, B) and the proportional incident cases, DALYs (C, D) of 29 cancers among men and women in China in 2019

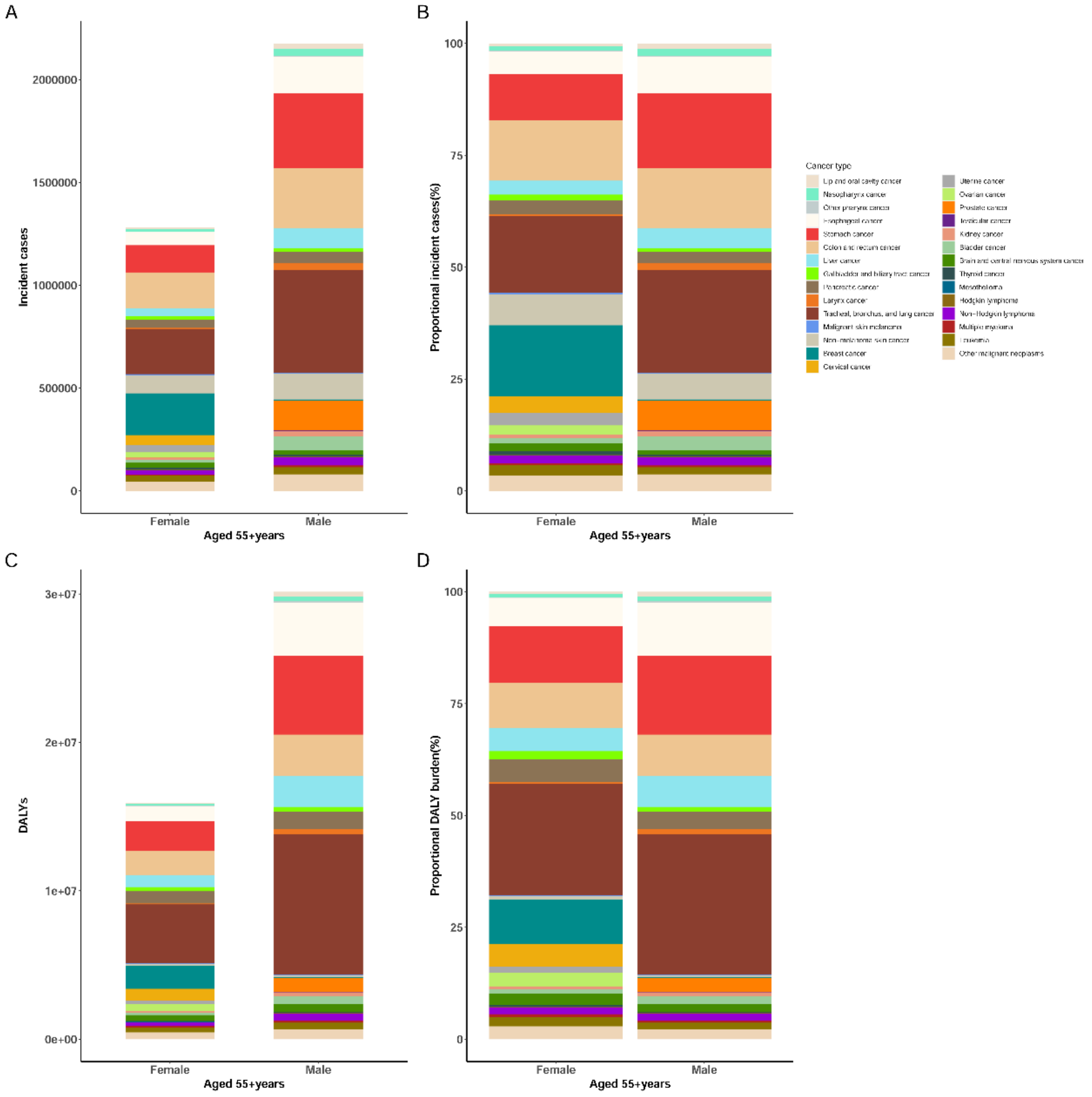

Note: Estimates are for populations aged 55 years and above among men and women in China. DALY=disability-adjusted life-year.

Figure S4 Ratio of men to women incidence rate (A) and ratio of men to women DALY rate (B) of 24 cancers (the cancers can affect both men and women) in China from 1990 to 2019

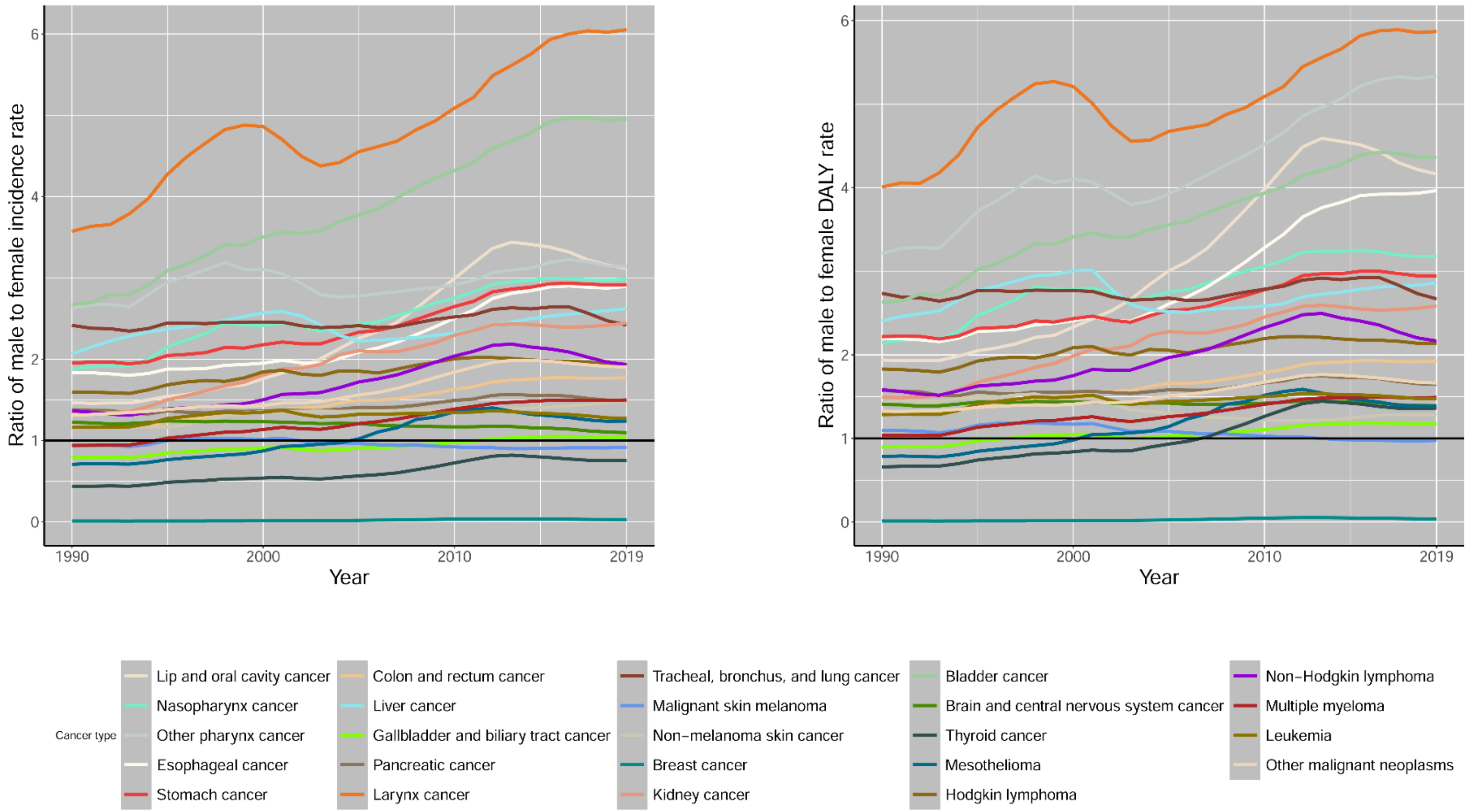

Note: Estimates are for populations aged 55 years and above. DALY=disability-adjusted life-year.

Figure S5 Incident cases (A), death cases (C), DALYs (E) and the proportional incident cases (B), proportional deaths (D), proportional DALYs (F) of 29 cancers among China, United States, Western Europe in 2019

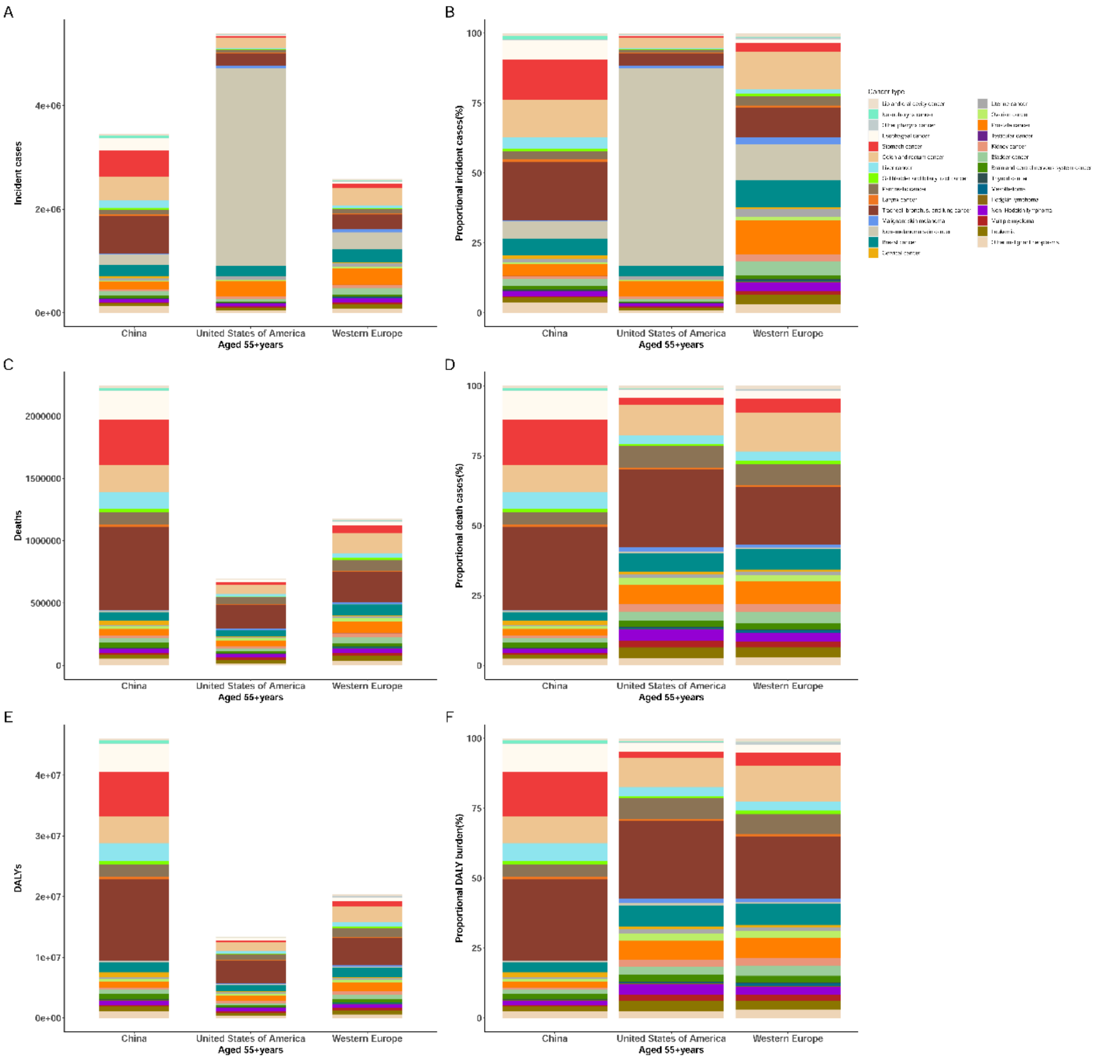

Note: Estimates are for populations aged 55 years and above. DALY=disability-adjusted life-year.

Figure S6 The change trend of ASIR in 29 cancers among China, United States, Western Europe from 1990 to 2019

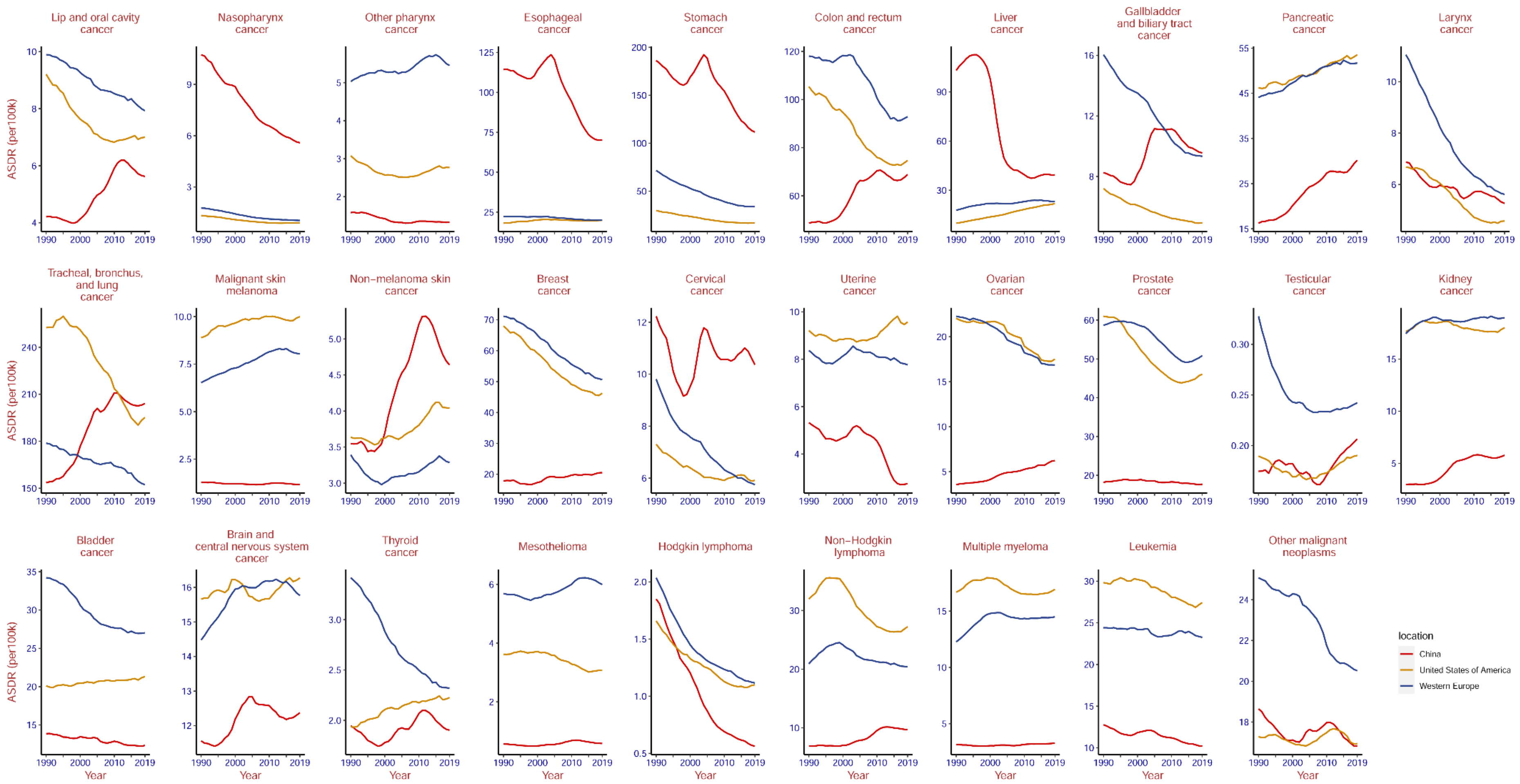

Note: Estimates are for populations aged 55 years and above. Rates are reported per 100000 person-years. ASIR=age-standardized incidence rate.

Figure S7 The change trend of ASDR in 29 cancers among China, United States, Western Europe from 1990 to 2019

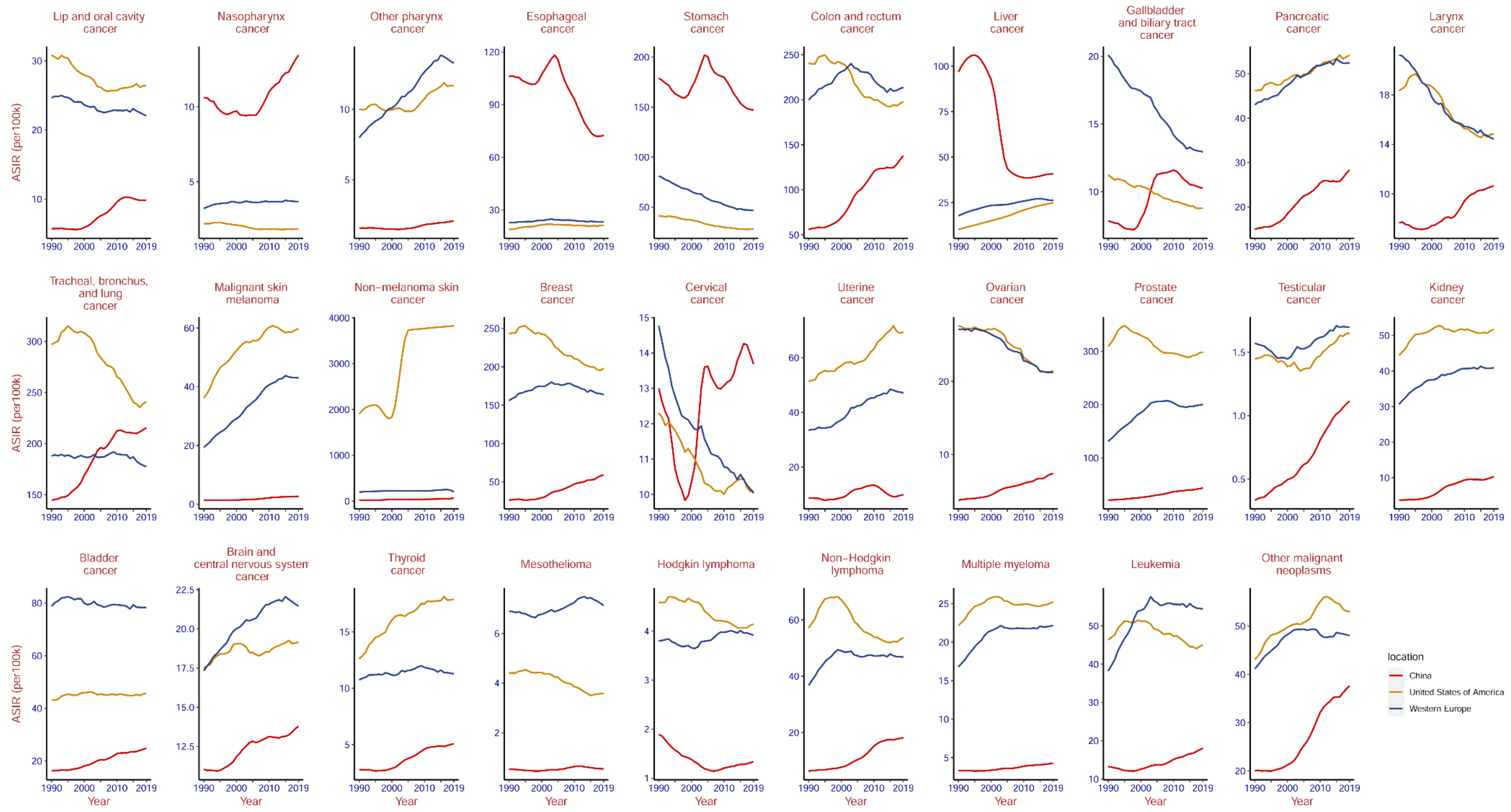

Note: Estimates are for populations aged 55 years and above. Rates are reported per 100000 person-years. ASDR=age-standardized death rate.
